# Selective Chlorination of Toluene and Halobenzenes Using Modified BaSO_4_-Supported Catalysts: A Sustainable Approach with (NH_4_)_2_S_2_O_8_ and H_2_O_2_ as Oxidants

**DOI:** 10.3390/nano16171120

**Published:** 2026-09-06

**Authors:** Sidra Chaudhary, Sumaira Jamal, Mohsin Alam, Yuan Gao, Muhammad Faisal Altaf, Junsheng Bai, Yang Sun

**Affiliations:** 1 Department of Applied Chemistry, School of Chemistry, Xi’an Jiaotong University, No. 28, Xianning West Road, Xi’an 710049, China; sidra-ch576@stu.xjtu.edu.cn (S.C.); sumairajamal980@stu.xjtu.edu.cn (S.J.); mohsinalam57@outlook.com (M.A.); 2 Shaanxi Fuhua Chemical Co., Ltd., 2nd Industrial Road East, Zhuangli Industrial Zone, Fuping County, Weinan 711711, China; gaoyuan@fuhua-chem.com (Y.G.); m.faisalaltafch@gmail.com (M.F.A.); 13571376365@163.com (J.B.); 3 Xi’an Biomass Green Catalysis and Advanced Valorization International Science and Technology Cooperation Base, No. 28, Xianning West Road, Xi’an 710049, China

**Keywords:** modified barium sulfate, catalytic chlorination, ammonium persulfate, ammonium hydrogen sulfate, ammonium chloride, chlorobenzene

## Abstract

In this study, nine barium sulfate (BaSO_4_)-supported heterogeneous catalysts (C1–C9) were synthesized via three modification strategies: stearic acid coating (C1–C3), physical doping (C4, C6, and C8), and sol-gel processing with calcination (C5, C7, and C9). Their comprehensive characterization revealed that sol-gel-synthesized C7 exhibited the most favorable surface properties, including well-dispersed Al–O–Si species, tetrahedrally coordinated Al^3+^, and abundant Brønsted acid sites. Their catalytic performance was evaluated in the chlorination of toluene, fluorobenzene, bromobenzene, and iodobenzene, using hydrochloric acid (HCl) as the chlorine source and either hydrogen peroxide (H_2_O_2_) or ammonium persulfate ((NH_4_)_2_S_2_O_8_) as the oxidant. C7 achieved complete toluene conversion (100%) at 60 °C under optimized conditions and exhibited high conversions of fluorobenzene (55%), bromobenzene (76%), and iodobenzene (46%). Notably, ammonium persulfate enabled a unique in situ halogen exchange pathway, yielding chlorobenzene as the exclusive product from bromobenzene and iodobenzene. XRD and XPS analysis of crystalline by-products confirmed the formation of NH_4_HSO_4_, BaSO_4_, and NH_4_Cl, providing evidence for the persulfate-driven radical mechanism. Iodine detection in upper-layer crystals confirmed iodobenzene products, while the absence of chlorine signals in the upper layer confirmed separation of organic and inorganic species. The detection of barium sulfate peaks confirms that the catalyst support retains its structural integrity under harsh reaction conditions, demonstrating chemical stability and reusability potential. Collectively, these findings establish a clear structure–activity relationship and demonstrate that the synergy between modified BaSO_4_ surfaces and persulfate-generated radicals provides an efficient, sustainable platform for aromatic chlorination, offering significant potential for pharmaceutical, agrochemical, and fine chemical manufacturing applications.

## 1. Introduction

Halogenated organic compounds constitute one of the most fundamental and versatile classes of molecules in synthetic chemistry, pharmaceutical research, and materials engineering [1]. Halogenation frequently serves as the initial step in the synthesis of natural products. The strategic incorporation of halogen atoms into organic frameworks plays a critical role in modern synthetic chemistry, enabling precise modulation of physicochemical properties and biological activities. The pervasive presence of halogenated compounds in natural systems, pharmaceuticals, agrochemicals, and functional materials underscores their essential status in contemporary science and technology [2]. Nature has long recognized the utility of halogenation, as evidenced by the discovery of over 4700 naturally occurring organohalogenated molecules. These natural products display a remarkable diversity of biological activities, including anticancer, antibiotic, antifungal, and antiviral effects, highlighting the significant influence of halogen substituents on molecular recognition and biological function [3].

The influence of halogen atoms on biological activity is demonstrated by clinically significant antibiotics, such as vancomycin, where the presence of chlorine substituents is essential for maintaining the active conformation and antibacterial activity against methicillin-resistant Staphylococcus aureus (MRSA) [4]. The therapeutic efficacy of rebeccamycin, an indolocarbazole natural product, also depends on its chlorine atoms, as deschloro derivatives lack antimicrobial activity [5]. These examples illustrate that halogens are not merely passive substituents but actively participate in defining the biological profile of natural products.

Beyond the scope of natural products, halogenated compounds also constitute a significant proportion of FDA-approved pharmaceuticals, with chlorine appearing in more than 200 approved drugs that play pivotal roles in treating respiratory, antiretroviral, cardiovascular, and other life-threatening diseases [6]. The 2021 FDA approvals included 14 halogenated drugs, with 8 containing fluorine and 4 containing chlorine, underscoring the continued importance of halogen incorporation in drug discovery [7].

The introduction of halogen atoms into organic molecules has historically relied on various halogenating agents, ranging from elemental halogens to more sophisticated reagents. Molecular chlorine (Cl_2_) and bromine (Br_2_) have been the traditional sources of halogenation, but their toxicity, corrosiveness, and handling difficulties have urged the development of a safer alternative [8]. The theoretical limit for chlorine utilization in direct chlorination with Cl_2_ is only 50%, with the remaining chlorine being converted to hydrogen chloride (HCl) as an undesirable by-product that often requires energy-intensive recycling or disposal as industrial waste [9]. To address such types of limitations, various chlorinating agents have been developed, including N-halosuccinimides (NCS, NBS, and NIS), sulfuryl chloride (SO_2_Cl_2_), phosphorus halides, and trichloroisocyanuric acid (TCCA) [10]. However, most of them often require activation through Lewis or Brønsted acids, metal catalysts, or photochemical conditions to achieve satisfactory reactivity with less activated substrates [11]. The development of catalytic systems enabling chlorination using inexpensive inorganic chloride salts, such as NaCl, KCl, NH_4_Cl, MgCl_2_, and CaCl_2_, has gained considerable attention as a more sustainable and atom-economical approach [12]. Among various chlorine sources, hydrochloric acid (HCl) stands out as a particularly attractive and environmentally benign option. Unlike other chlorinating agents, HCl is abundantly available as an industrial by-product waste stream, making it highly cost-effective and safer to handle and eliminating the need for dedicated chlorine production [13].

The oxidation of chloride sources (such as HCl, NaCl, or KCl) to generate active chlorinating species is a fundamental strategy in oxidative chlorination, offering the advantage of 100% halogen atom economy when combined with appropriate terminal oxidants [14]. Hydrogen peroxide (H_2_O_2_) has emerged as one of the most attractive oxidants for this purpose, as it produces only water as a by-product and aligns with green chemistry principles [14,15]. The H_2_O_2_/HCl system has been extensively studied for chlorination reactions, generating hypochlorous acid (HOCl) and molecular chlorine in situ through the oxidation of chloride [16]. However, H_2_O_2_-mediated chlorination suffers from limitations, including the thermal decomposition of H_2_O_2_ at elevated temperatures, the competitive reduction of active chlorine species, and the requirement for acidic conditions to achieve a reasonable rate [17]. The H_2_O_2_/HCl system has been successfully applied for the chlorination of various substrates, including arenes, alkenes, and ketones, though selectivity remains a significant challenge, particularly for less activated substrates [15,16].

Other oxidants have been explored for oxidative halogenation, including Oxone (2KHSO_2_·KHSO_4_·K_2_SO_4_), sodium persulfate (Na_2_S_2_O_8_), and molecular oxygen. The Oxone/KCl system has shown results for the chlorination of alkyl aromatics, with solvent-dependent regioselectivity enabling either *α*- or aromatic chlorination [18]. Most recently, ammonium persulfate ((NH_4_)_2_S_2_O_8_) has attracted interest as a powerful oxidant capable of generating sulfate radical anions (SO_4_^−^**∙**) through thermal or catalytic activation [19]. The high oxidation potential of sulfate radicals enables the oxidation of chloride sources even at elevated temperatures where H_2_O_2_ decomposition would be problematic [20]. The use of ammonium persulfate offers the additional advantage of producing ammonium hydrogen sulfate (NH_4_HSO_4_) as a recoverable by-product, facilitating catalyst recycling and waste minimization [13,21].

A wide array of catalysts has been developed to facilitate halogenation reactions, ranging from homogeneous transition metal complexes to heterogeneous solid acid catalysts and nanomaterials. Palladium-based catalysts have been extensively explored for directed C–H halogenation, enabling ortho-selective functionalization of benzyl nitriles, Weinreb amides, and anilides through cyclopalladation intermediates [22]. Manganese porphyrins have demonstrated remarkable catalytic activity for aliphatic C–H chlorination, operating through a mechanism involving oxomanganese(V) intermediates and a radical rebound pathway [23]. The sterically bulky Mn(TMP)Cl catalyst exhibited exceptional selectivity for methylene C–H bonds over weaker methine positions, providing access to chlorinated products that would be difficult to obtain through traditional radical chlorination [23]. Iron-based catalysts have similarly shown promise for C–H chlorination, with FeCl_3_ enabling visible-light-induced chlorination of C–H bonds using MgCl_2_ as the chlorine source [24].

Sulfated metal oxides represent another important class of heterogeneous catalysts for halogenation. Sulfated tin oxide (STO) has demonstrated exceptional selectivity (>96%) for methane chlorination to methyl chloride, achieving remarkable performance even at high methane conversion [20]. The strong Lewis acid sites on STO, generated by the interaction of Sn with surface sulfate groups, facilitate heterolytic cleavage of Cl_2_ and promote electrophilic chlorination through an ionic mechanism rather than the nonselective radical pathway typical of thermal chlorination [20]. Silica-supported sulfate catalysts prepared by incorporating SO_3_H groups onto silica surfaces have proven effective for various acid-catalyzed reactions, including Baeyer–Villiger oxidations and esterifications [24]. The immobilization of sulfate groups onto silica offers the advantages of high surface area, strong acidity, and recyclability, making these materials attractive for heterogeneous catalysis [24].

Barium sulfate (BaSO_4_), though primarily known as a filler and contrast agent, has recently emerged as a promising catalyst support due to its low cost, high stability, high density (4.2–4.48 g/cm^3^), moderate hardness, non-magnetic and non-toxic, insoluble in water, and highly resistant to acids, especially to HCl [25,26]. In recent years, researchers have searched for many new and alternative minerals, including limestone [27,28]. The surface modification of BaSO_4_ particles with various functional groups has been extensively investigated to improve their compatibility with polymer matrices and enhance their catalytic performance [27,29]. Stearic acid modification effectively converts hydrophilic BaSO_4_ surfaces to hydrophobic ones, improving dispersion in organic media and enabling the formation of nanocomposites with enhanced mechanical properties [27]. The incorporation of silica and alumina components onto BaSO_4_ surfaces through sol-gel processing can generate composite materials with superior thermal stability, acidity, and catalytic activity [30]. Despite these advances, the application of modified BaSO_4_ as a catalyst support for chlorination reactions remains largely unexplored [25,31].

The reaction conditions for chlorination, i.e., temperature, solvent, catalyst, and oxidant, significantly affect efficiency and selectivity. Photochlorination of toluene requires careful control to balance ring versus side-chain chlorination, with industrial processes operating above 70 °C at significant energy cost [32]. Among various solvents, water stands out as the most environmentally benign and cost-effective medium for chlorination [33,34], and aqueous media offer distinct advantages, i.e., non-toxic, non-flammable, and compatible with green chemistry principles. Furthermore, micellar systems in water have achieved remarkable ortho/para-selectivity ratios up to 12 for phenol chlorination [35].

Toluene and its derivatives are particularly important substrates for chlorination due to their industrial relevance and the competing pathways available for ring versus side-chain chlorination [13,32]. The chlorination of toluene can follow either electrophilic aromatic substitution to yield ortho- and para-chlorotoluene or radical chain chlorination to produce benzyl chloride, or benzyl alcohol, with the dominant pathway determined by reaction conditions and catalyst choice [34,36,37]. Among chlorination mechanisms, electrophilic aromatic substitution (EAS) and ammonium-persulfate-initiated radical pathways are the most superior. EAS provides reliable regioselectivity for activated arenes via σ-complex intermediates [36,38]. Halogenated benzenes, such as fluorobenzene, bromobenzene, and iodobenzene, also serve as important substrates for chlorination, though their deactivated nature makes electrophilic substitution more challenging [22].

Despite these advances, the development of a simple, efficient, and sustainable catalytic system for aromatic chlorination using abundant and inexpensive materials remains a significant challenge [39]. Many reported methods rely on noble metal catalysts, harsh reaction conditions, specialized solvents, or photochemical setups that limit their practical applicability [40]. Furthermore, the use of inorganic chloride salts as chlorine sources, while attractive from an atom economy perspective, often requires strong chemical oxidants or complex activation protocols [41]. There remains a clear need for heterogeneous catalysts that can efficiently activate HCl, an abundant industrial waste product, under mild conditions while providing high selectivity [42,43].

In summary, the catalytic systems developed in this work, i.e., modified barium sulfate (BaSO_4_) catalysts, serve as heterogeneous supports providing surface acidic sites (Al–OH and Si–OH) that facilitate the activation of HCl and the formation of electrophilic chlorine species, with (NH_4_)_2_S_2_O_8_ acting as the radical initiator and with H_2_O_2_ serving as the green oxidant. The synergistic interaction among these three components, i.e., catalyst, oxidant, and chlorine source, is expected to yield significant enhancement in aromatic chlorination performance. The sol-gel-synthesized catalysts C5 and C7 demonstrated superior activity due to their high surface area, porous network, and abundant acidic sites, achieving complete conversion of toluene and efficient chlorination of fluorobenzene, bromobenzene, and iodobenzene. Notably, the ammonium persulfate system enabled a unique in situ halogen exchange pathway with bromobenzene and iodobenzene, yielding chlorobenzene as a distinctive product alongside electrophilic aromatic substitution products. The identification of crystalline by-products (NH_4_HSO_4_ and NH_4_Cl) from the persulfate system provides direct evidence for the radical mechanism and confirms the structural integrity of the BaSO_4_ catalyst. In addition, detailed characterization of catalysts has been conducted to elucidate the structure–activity relationships underlying the catalytic performance. This work holds considerable scientific and practical value for advancing sustainable and cost-effective chlorination methodologies suited for pharmaceutical, agrochemical, and fine chemical manufacturing applications.

## 2. Materials and Methods

### 2.1. Materials

Ultrafine barium sulfate powder (BaSO_4_, 99%) was provided by Shaanxi Fuhua Chemical Co., Ltd. (Weinan, China). Stearic acid (CH_3_(CH_2_)_16_COOH, SA, 99%), anhydrous sodium sulfate (Na_2_SO_4_, 99%), silica powder (silica, SiO_2_, 300 mesh 99.5%), *α*-alumina powder (*α*-Al_2_O_3_, 99%), aluminum hydroxide (Al(OH)_3_, 99%), tetraethyl orthosilicate (Si(OCH_2_CH_3_)_4_, TEOS, 98%), ammonium persulfate ((NH_4_)_2_S_2_O_8_, 98.5%), hydrogen peroxide aqueous solution (H_2_O_2_, 30 wt.%), and hydrochloric acid (HCl, 37 wt.%) were purchased from Shanghai Aladdin Biochemical Technology Co., Ltd. (Shanghai, China). Toluene (99.5%), fluorobenzene (99%), bromobenzene (99%), iodobenzene (98%), dichloromethane (CH_2_Cl_2_, 99.5%), and anhydrous magnesium sulfate (MgSO_4_, 98%) were purchased from Shanghai Macklin Biochemical Technology Co., Ltd. (Shanghai, China). Distilled water was prepared by the laboratory Milli-Q pure water system.

### 2.2. Instruments

A YJKS high-energy ball milling machine (Foshan Tenghao Instrument Technology Co., Ltd., Foshan, China) with a two-vessel configuration was used for mechanical treatment, operating at 220 V and 370 W, and charged with 18 mm-diameter zirconia (ZrO_2_) grinding media.

X-ray photoelectron spectroscopy (XPS) spectra were recorded on a Kratos Axis Ultra DLD spectrometer (Kratos Co., Manchester, UK) using monochromatic Al K*α* (1486.6 eV, 150 W). Binding energies were referenced to C 1s (284.8 eV). Data processing was done with CasaXPS using a Gaussian–Lorentzian function (70:30) after Shirley background subtraction.

X-ray diffraction (XRD) was performed on a Philips X’Pert Pro diffractometer (PANalytical) using Cu K*α* radiation (*λ* = 1.5418 Å, 40 kV, 40 mA) over 2*θ* = 10–80° at 0.05° s^−1^. Phase identification was carried out with MDI Jade software (version 9) using ICDD/JCPDS reference data. Fourier transform infrared (FT-IR) spectra were collected on a Bruker VERTEX 70 spectrometer (Bruker, Berlin, Germany) using the ATR technique in the 400–4000 cm^−1^ range.

Scanning electron microscopy (SEM) imaging and energy-dispersive X-ray spectroscopy (EDS) elemental analysis were performed on a Carl Zeiss GeminiSEM 500 field emission scanning electron microscope (Carl Zeiss, Shanghai, China) at accelerating voltages of 1.0–15.0 kV, equipped with an Oxford Instruments Ultim Max EDS detector (Oxford Instruments, Abingdon, UK).

Ultraviolet-visible (UV-Vis) absorption spectra were acquired on a PerkinElmer Lambda 950 double-beam spectrophotometer (PerkinElmer, Waltham, MA, USA) in the spectral range of 250–800 nm. The catalyst samples were dispersed in distilled water at a concentration of 2.5 mg mL^−1^, and the baseline correction was performed using distilled water as the reference.

Aqueous particle size and aqueous zeta potential were measured using a Malvern Zetasizer Nano ZSE (Malvern Panalytical, Malvern, UK) at 25 °C. Samples were prepared by dispersing the catalysts in deionized water (0.1 mg mL^−1^) with ultrasonication for 10 min prior to analysis.

Thermogravimetric analysis (TGA) and derivative thermogravimetry (DTG) were performed on a METTLER TOLEDO instrument (Mettler-Toledo International Inc., Greifensee, Switzerland) under atmosphere of air (50 mL min^−1^) over a temperature range of 30–800 °C at a heating rate of 10 °C min^−1^. Differential scanning calorimetry (DSC) was carried out separately on a METTLER TOLEDO DSC 3 instrument (Mettler-Toledo International Inc., Greifensee, Switzerland) over the temperature range of 30–300 °C at a heating rate of 10 °C min^−1^.

Gas chromatography–mass spectrometry (GC-MS) analysis was conducted using the Shimadzu GC-MS-QP2010 Plus system, equipped with an Rxi-5ms capillary column (30 m × 0.25 mm × 0.25 μm). The injection port temperature was 250 °C, with split injection (split ratio 26:1), and the carrier gas was helium (1.0 mL min^−1^). The ion source and interface temperatures were maintained at 200 °C and 250 °C, respectively, in the electron impact ionization (EI, 70 eV) mode.

### 2.3. Preparation of Catalysts

**Figure 1:**
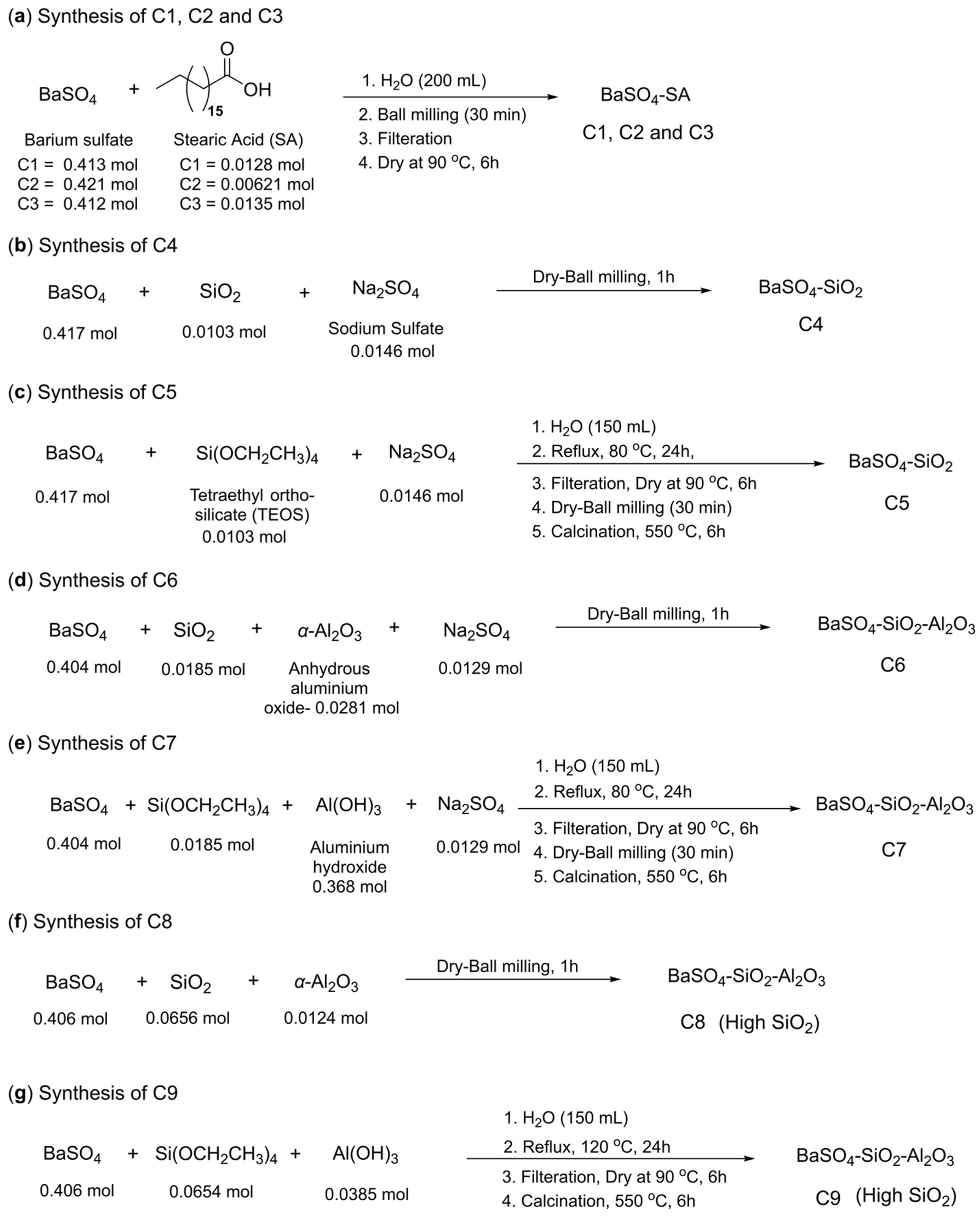
Synthesis of catalysts.

The flow chart of admixture preparation is presented in Figure 1.

The synthesis of catalysts C1–C9 was carried out using three distinct modification approaches: organic coating with stearic acid (C1–C3), physical doping with inorganic additives (C4, C6, and C8), and sol-gel processing with subsequent calcination (C5, C7, and C9). The flow chart of catalyst preparation is presented in Figure 1.

#### 2.3.1. Stearic Acid Coating (C1–C3)

For preparation of catalyst C1, ultrafine BaSO_4_ powder (96.35 g, 0.413 mol) and stearic acid (3.65 g, 0.0128 mol) were added to a planetary ball milling jar along with 200 mL of deionized water and grinding beads. The mixture was ball-milled for 30 min, followed by vacuum filtration, thorough washing with deionized water, and drying in an oven at 90 °C for 6 h.

For C2, BaSO_4_ powder (98.26 g, 0.421 mol) and stearic acid (1.74 g, 0.00612 mol) were processed following the same procedure as C1.

For C3, BaSO_4_ powder (96.17 g, 0.412 mol) and stearic acid (3.83 g, 0.0135 mol) were processed following the same procedure as C1.

#### 2.3.2. Physical Doping (C4, C6, and C8)

For preparation of catalyst C4, BaSO_4_ (97.30 g, 0.417 mol), anhydrous Na_2_SO_4_ (2.08 g, 0.0146 mol), and silica powder (0.62 g, 0.0103 mol) were dry-milled for 1 h. After vacuum filtration, the resulting solid was washed with distilled water and subsequently dried in an oven at 90 °C for 6 h, affording solid C4.

For C6, BaSO_4_ (94.19 g, 0.404 mol), *α*-Al_2_O_3_ (2.87 g, 0.0281 mol), SiO_2_ (1.11 g, 0.0185 mol), and Na_2_SO_4_ (1.83 g, 0.0129 mol) were dry-milled for 1 h. The post-treatment procedure for C6 is identical to that for C4.

For C8, BaSO_4_ (94.80 g, 0.406 mol), *α*-Al_2_O_3_ (1.26 g, 0.0124 mol), and SiO_2_ (3.94 g, 0.0656 mol) were dry-milled for 1 h. The post-treatment procedure for C8 is identical to that for C4.

#### 2.3.3. Sol-Gel Processing with Calcination (C5, C7, and C9)

For preparation of C5, BaSO_4_ (97.30 g, 0.417 mol), Na_2_SO_4_ (2.08 g, 0.0146 mol), and TEOS (2.15 g, 0.0103 mol, equimolar to 0.62 g of SiO_2_) were dispersed in 150 mL of deionized water. The mixture was refluxed with stirring at 80 °C for 24 h in a round-bottomed flask. The resulting solid was recovered by filtration, dried, ball-milled, and calcined at 550 °C in a muffle furnace for 6 h.

For C7, BaSO_4_ (94.19 g, 0.404 mol), Al(OH)_3_ (2.87 g, 0.0368 mol), TEOS (3.85 g, 0.0185 mol, equimolar to 1.11 g of SiO_2_), and Na_2_SO_4_ (1.83 g, 0.0129 mol) were refluxed in 150 mL of deionized water for 24 h. The resulting solid was dried, ball-milled, and calcined at 550 °C for 6 h.

For C9, BaSO_4_ (94.80 g, 0.406 mol), TEOS (13.63 g, 0.0654 mol), and Al(OH)_3_ (3.00 g, 0.0385 mol) were refluxed with stirring in 150 mL of deionized water at 80 °C for 24 h. The product was filtered, washed, dried at 90 °C for 6 h, and calcined in a muffle furnace at 550 °C for 6 h.

### 2.4. Catalytic Chlorination Reactions

The synthesized catalyst (C1–C9, 10~50 mg) and relevant substrate (i.e., toluene, fluorobenzene, bromobenzene, or iodobenzene, 2.0 mmol) and HCl (aqueous solution, 37 wt.%, 8 mmol pure HCl) were added in a 250 mL three-necked round-bottom flask equipped with a condenser, dropping funnel, and an oil bath. Under magnetic stirring, the oxidant H_2_O_2_ or (NH_4_)_2_S_2_O_8_ (0.5~2.5 mmol, as shown in Tables 1–4) was added drop by drop through a constant pressure dropping funnel. Then, we raised the temperature of the reaction (up to 60~120 °C, depending on the specific reaction conditions), continued magnetic stirring, and allowed the reaction mixture to react at this temperature for 6 h. After the reaction was completed, the mixture was cooled down to room temperature and treated by the solvent extraction method with CH_2_Cl_2_ solvent to separate the chlorinated product (organic layer) from the inorganic layer (which includes catalyst, unreacted oxidant, water solvent, etc.), and it was washed three times with this solvent to get maximum separation of product. Then, to remove the moisture content from the organic layer, the organic layer was stirred with 0.5 g of anhydrous MgSO_4_ for half an hour then filtered with an organic syringe filter. The obtained organic filtrate was analyzed by GC-MS to determine the chlorination conversion rate, product yield, and unreacted substrate yield. GC-MS analysis employs the area normalization method, and neither external nor internal standards are used.

In the iodobenzene reaction, as the chlorinated iodobenzene product is solid due to its high molecular weight, an interesting phenomenon was observed. Several days before complete drying, two types of crystals were observed forming in the upper and lower layers of the organic phase. These crystal products were collected and analyzed by XRD and XPS.

## 3. Results and Discussion

### 3.1. Synthesis of Catalysts

The nine modified barium sulfate catalysts (C1–C9; Figure 1) were synthesized through three distinct approaches: organic surface coating with stearic acid (C1–C3), physical doping with inorganic additives (C4, C6, and C8), and sol-gel processing with subsequent calcination (C5, C7, and C9). These strategies were designed to systematically investigate the influence of surface modification and compositional variation on catalytic performance in aromatic chlorination reactions. For the stearic-acid-coated catalysts (C1–C3), stearic acid was selected as an organic modifier to convert the inherently hydrophilic BaSO_4_ surface to a hydrophobic one, improving particle dispersion and compatibility with organic substrates in the reaction medium [27,29]. The carboxylate head group of stearic acid is expected to interact with the positively charged Ba^2+^ sites on the BaSO_4_ surface through electrostatic interactions or coordination bonding, while the long hydrophobic alkyl chain extends outward, creating an organic layer. The varying stearic acid loadings (1.74–3.83 g) were employed to investigate the optimal surface coverage for catalytic activity. Indeed, during the synthesis of catalysts C1–C3, the drying step at 90 °C ensures both the removal of water and the stability of the chemical composition of stearic acid (Figure 1a). In the physically doped catalysts (C4, C6, and C8), inorganic additives (SiO_2_, *α*-Al_2_O_3_, and Na_2_SO_4_) were incorporated through simple dry-ball milling without any chemical treatment, allowing for the introduction of additional acidic sites (Si–OH and Al–OH) onto the BaSO_4_ surface, which are known to facilitate electrophilic chlorination through polarization of chlorine species [20,24]. For the sol-gel-processed catalysts (C5, C7, and C9), the approach was employed to achieve more intimate mixing and chemical bonding between the BaSO_4_ support and the inorganic dopants. Hydrolysis and condensation of TEOS in the presence of BaSO_4_ generate a silica network that coats the particle surface [24]. Similarly, Al(OH)_3_ was incorporated either directly (C7 and C9) or as a precursor (C5 through TEOS only), with subsequent calcination at 550 °C converting it to amorphous or nanocrystalline alumina species. This approach is expected to generate a porous, high-surface-area catalytic material with abundant surface hydroxyl groups (Si–OH and Al–OH) that serve as both Lewis and Brønsted acid sites [25], with the calcination step also ensuring thermal stability of the catalysts under the reaction conditions.

### 3.2. Characterizations of Catalysts

**Figure 2:**
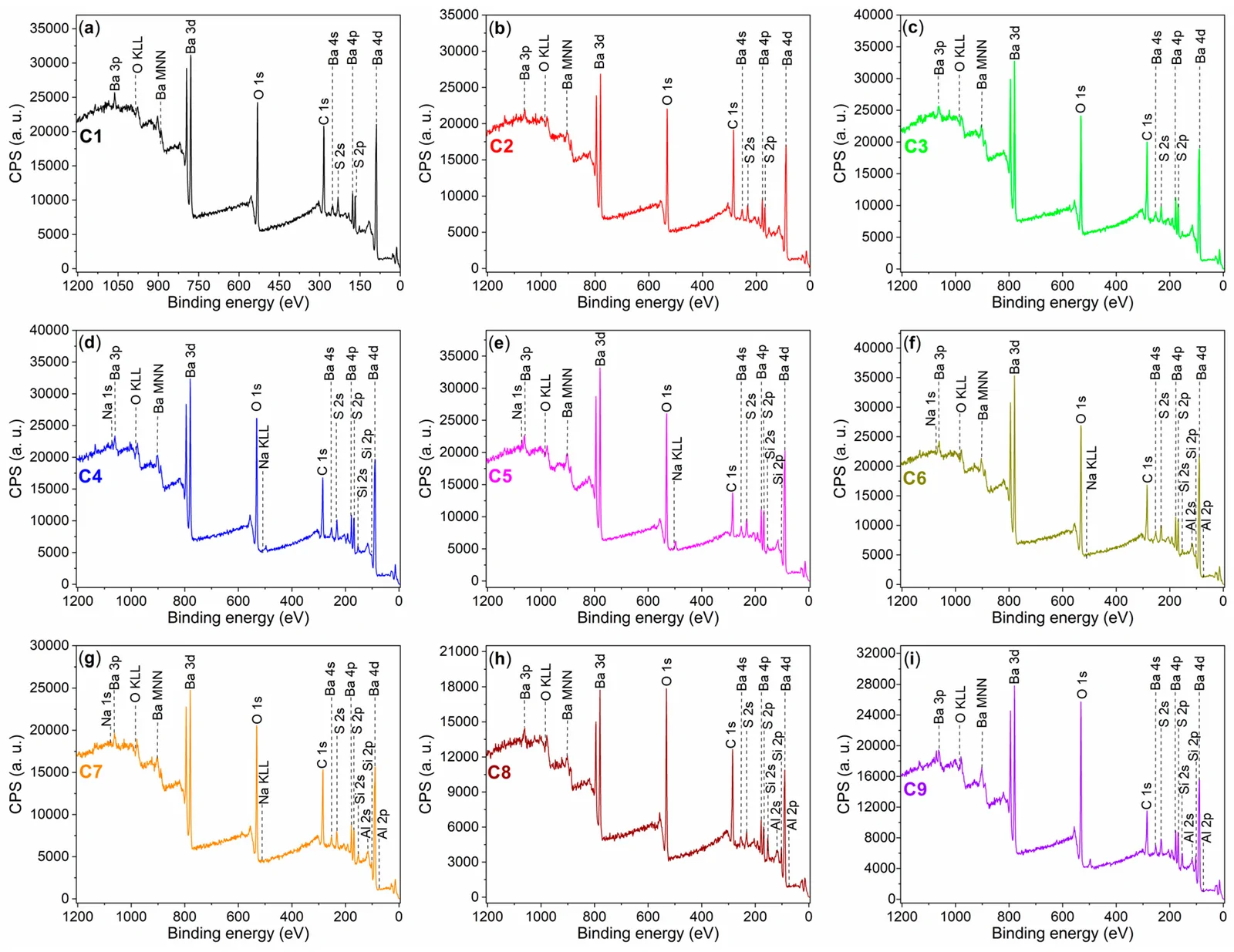
XPS survey scans of synthesized catalysts: (**a**) C1; (**b**) C2; (**c**) C3; (**d**) C4; (**e**) C5; (**f**) C6; (**g**) C7; (**h**) C8; (**i**) C9.

Binding energies and atomic compositions on surfaces of synthesized catalysts C1–C9 were investigated within a detection depth of 0–10 nm by X-ray photoelectron spectroscopy (XPS; Figure 2; Table S1, Figures S1–S7, Supplementary Material). The full-spectrum scan in Figure 2 confirmed the presence of Ba, S, O, and C in all samples, as well as Si, Al, and Na corresponding to the synthetic modifiers. Detailed binding energies and atomic percentages are summarized in Table S1.

The O 1s spectra (Figure S1) were split into two components for all catalysts. The lower binding energy (BE) component (530.8–531.8 eV) is assigned to lattice oxygen in the BaSO_4_ framework [44], while the higher BE component (532.0–533.3 eV) corresponds to surface hydroxyl groups, Si–O–Si, Al–O–Si, and C–O species [45]. For C7 (the best-performing catalyst), peaks were observed at 531.4 eV and 532.8 eV (Figure S1g). The higher BE component at 532.8 eV is attributed to a combination of Si–O–Si bridging oxygen, Al–O–Si interfacial oxygen, and surface hydroxyl groups. The presence of this well-defined Al–O–Si component is significant, as it indicates successful incorporation of aluminum into the silica network, creating strong Brønsted acid sites that are crucial for electrophilic chlorination activity. C5 (the second-best catalyst) exhibited peaks at 531.2 eV and 532.2 eV (Figure S1e), with the higher BE component corresponding to Si–O–Si and Si–OH groups from TEOS hydrolysis. The O 1s binding energy of C7 (532.8 eV) is higher than that of C5 (532.2 eV), suggesting stronger acid site generation in C7 due to Al incorporation.

The Ba 3d spectra (Figure S2) displayed the characteristic spin-orbit doublet for all catalysts. For C7, peaks were observed at 779.7 eV (Ba 3d_5/2_) and 795.0 eV (Ba 3d_3/2_; Figure S2g), with a spin-orbit splitting of 15.3 eV, characteristic of Ba^2+^ in the barium sulfate lattice [46]. The Ba 3d_5/2_ binding energy across all catalysts (779.6–780.0 eV) confirms that barium remains exclusively in the +2 oxidation state within the stable barite structure [47].

The S 2p spectra (Figure S3) exhibit doublet peaks at 164.5–165.5 eV (S 2p_3/2_) and 165.8–166.9 eV (S 2p_1/2_), characteristic of S^6+^ in SO_4_^2−^ of BaSO_4_ [48]. Consistent peak positions across all catalysts confirm sulfate integrity, with no sulfite, sulfide, or elemental sulfur detected. For C7, peaks were observed at 165.2 eV (S 2p_3/2_) and 166.9 eV (S 2p_1/2_; Figure S3g). The S 2p_3/2_ binding energy of 165.2 eV is consistent with sulfur in the tetrahedral sulfate environment of BaSO_4_ [49].

The C 1s spectra (Figure S4) were deconvoluted into three components: aliphatic C–C/C–H (284.4–284.8 eV), C–O (285.3–286.3 eV), and carboxylate O–C=O (288.4–288.8 eV). For C7, peaks were observed at 284.4 eV, 285.8 eV, and 288.4 eV (Figure S4e). The presence of carbon in C7 confirms residual organic species from TEOS precursor after calcination. C1–C3 exhibited higher carbon content (55.09–57.34 at%) due to stearic acid coating [50].

The Si 2p spectra (Figure S5) for Si-containing catalysts (C4–C9) exhibited one or two components. For C7, two peaks were observed at 101.6 eV and 102.7 eV (Figure S5d). The lower BE component at 101.6 eV is assigned to silicon in the SiO_2_ network [51], while the component at 102.7 eV corresponds to silicon in the Al–O–Si framework. This well-defined Al–O–Si component with lower binding energy (102.7 eV) compared to C6 (103.0 eV) suggests that sol-gel processing of C7 produced a more homogeneous aluminosilicate phase [51]. This is critical because Al–O–Si linkages create strong Brønsted acid sites when aluminum is incorporated into the silica framework. C5 showed a single peak at 102.2 eV, attributed to silicon in the cross-linked SiO_2_ network [44]. The absence of the higher BE component confirms that C5 contains no aluminum, consistent with its synthesis protocol (TEOS only). The Al–O–Si component observed only in C7 explains its superior catalytic performance compared to C5.

The Al 2p spectra (Figure S6) for Al-containing catalysts (C6–C8) exhibited single peaks. C7 showed a peak at 73.7 eV (Figure S6b), assigned to Al^3+^ in tetrahedral coordination within the aluminosilicate framework [52]. This binding energy is significantly lower than C6 (74.1 eV, octahedral Al^3+^ in *α-*Al_2_O_3_), confirming that aluminum is successfully incorporated into the silica network in a tetrahedral environment. The formation of tetrahedrally coordinated Al^3+^ generates strong Brønsted acid sites, as the Al^3+^ substitution creates a charge imbalance compensated by protons (Si–OH–Al). The presence of these strong acid sites in C7 is the key factor contributing to its superior catalytic performance in electrophilic chlorination. The Na 1s spectra (Figure S7) for C4 and C5 exhibited single peaks at 1071.3 eV, assigned to Na^+^ in Na_2_SO_4_.

XPS analysis confirms the successful surface modification of BaSO_4_ through different synthetic strategies. The key finding is that C7 exhibits unique surface chemistry, i.e., a well-defined Al–O–Si component in the Si 2p spectrum (101.6 eV and 102.7 eV), Al 2p at 73.7 eV indicating tetrahedrally coordinated Al^3+^ in the silica framework, and O 1s at 532.8 eV confirming Al–O–Si interfacial oxygen. These features generate strong Brønsted acid sites (Si–OH–Al) that are responsible for C7’s superior catalytic activity in electrophilic chlorination reactions. In contrast, C5 (without Al) only exhibits a single Si 2p peak at 102.2 eV and lacks acid sites, explaining its lower catalytic performance compared to C7.

**Figure 3:**
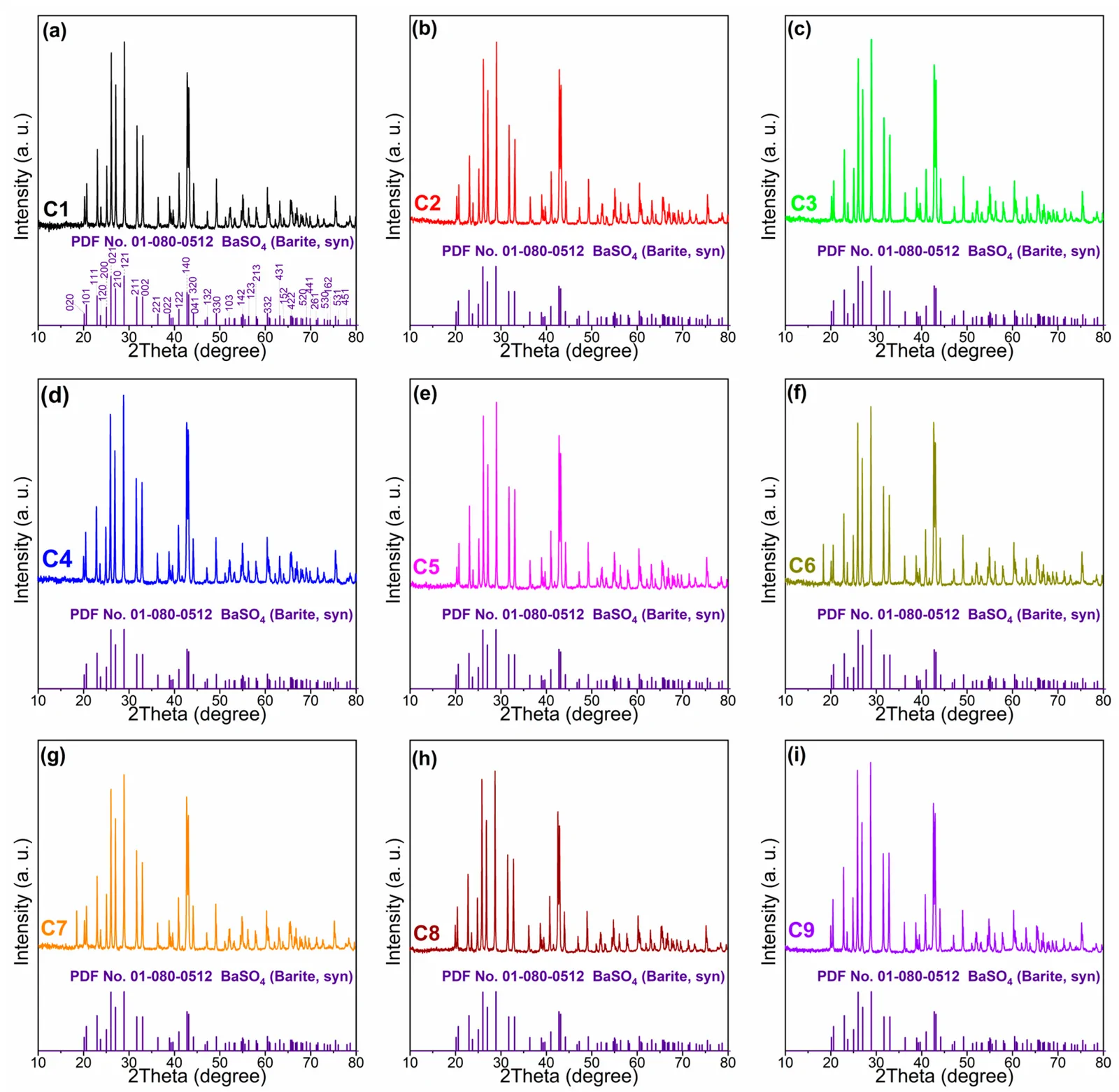
Wide-angle (2*θ* = 10–80°) XRD spectra of synthesized catalysts: (**a**) C1; (**b**) C2; (**c**) C3; (**d**) C4; (**e**) C5; (**f**) C6; (**g**) C7; (**h**) C8; (**i**) C9.

The crystal structure of the catalyst was analyzed by wide-angle XRD (Figure 3). All nine catalysts exhibited sharp and strong diffraction peaks matching those of the synthetic barite BaSO_4_ (JCPDS PDF No. 01-080-0512). The main diffraction planes are marked as (101), (111), (210), (121), (211), (121), (002), (1140), and (320) [39]. It is worth noting that no independent crystal diffraction peaks corresponding to SiO_2_ (such as quartz or cristobalite), *α*-Al_2_O_3_, Al(OH)_3_, or Na_2_SO_4_ were detected in the spectra of any sample [30]. This phenomenon where only barite phase is observed can be attributed to two factors. Firstly, the dopants (SiO_2_, Al_2_O_3_, and stearic acid) exist in a low-mass fraction and are distributed in the form of highly dispersed sub-nano amorphous layers or surface clusters, lacking long-range crystalline orderliness. Secondly, in the sol-gel-synthesized and calcined catalysts (C5, C7, and C9), the hydrolysis of TEOS and Al(OH)_3_ generates an amorphous, highly cross-linked silica-alumina surface coating layer, which coats the surface of BaSO_4_ particles without forming bulk crystalline oxide aggregates.

**Figure 4:**
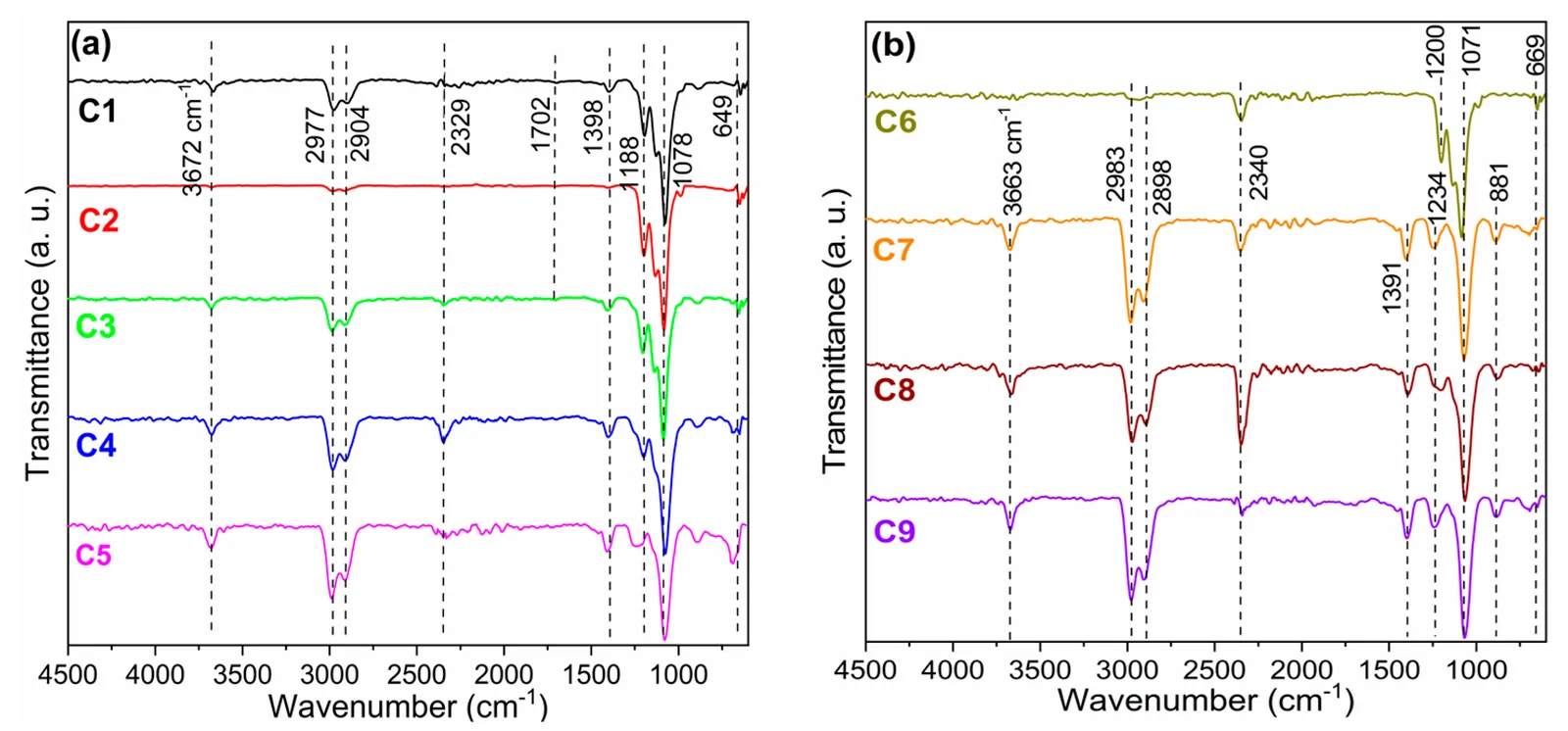
FT-IR spectra of synthesized catalysts: (**a**) C1, C2, C3, C4, C5; (**b**) C6, C7, C8, C9.

FT-IR spectroscopy (Figure 4) confirmed the surface functionalization of all catalysts, with all samples exhibiting characteristic SO_4_^−^ stretching and bending vibrations of BaSO_4_ at 1188, 1078, 983, 635, and 610 cm^−1^, along with surface O–H stretching at 3672 cm^−1^ [39]. C1–C3 (stearic-acid-coated) displayed sharp aliphatic C–H vibrations at 2977 and 2904 cm^−1^, C–H bending at 1398 cm^−1^, and CO_2_ absorption at 2329 cm^−1^, along with C=O stretching of carboxyl group at 1702 cm^−1^ [53], confirming stearic acid surface bonding, while C4–C9 showed enhanced Si–O–Si stretching at 1080–1100 cm^−1^ and Si–O bending at 795 cm^−1^, confirming silica incorporation. For C6–C9, Al–O vibrations appeared at 460–580 cm^−1^, with Si–O–Si and Al–O–Si stretching at 1234 and 881 cm^−1^, along with C–H peaks at 2983 and 2898 cm^−1^. Notably, C7 exhibited clear Al–O–Si stretching at 881 cm^−1^ (Figure 4b), confirming successful aluminum incorporation into the silica network, which generates Brønsted acid sites (Si–OH–Al) essential for electrophilic chlorination activity, whereas C5 showed only Si–O–Si vibrations without Al–O–Si features, consistent with its aluminum-free composition.

**Figure 5:**
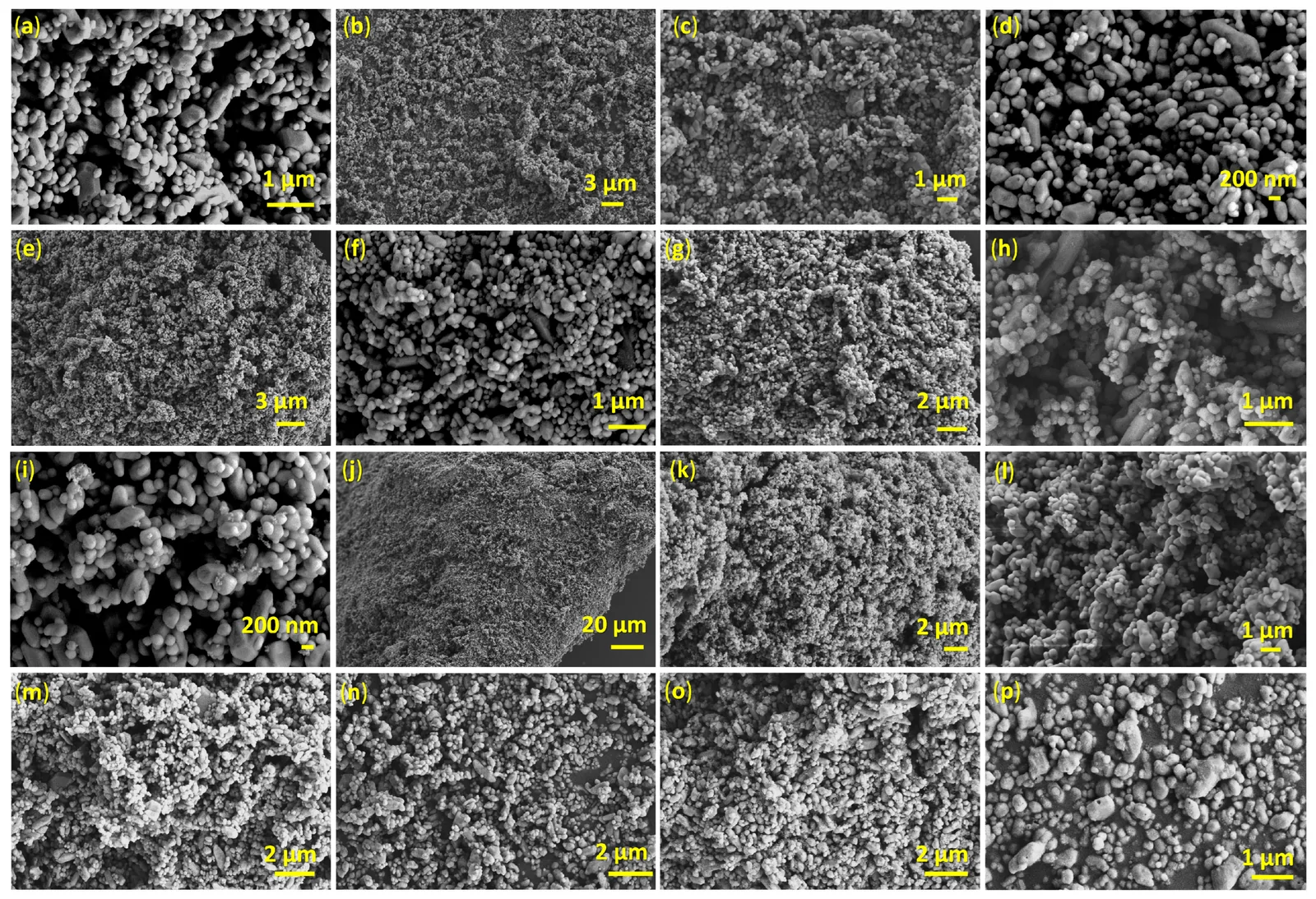
SEM images of synthesized catalysts: (**a**) C1, (**b**–**d**) C2, (**e**,**f**) C3, (**g**–**i**) C4, (**j**–**l**) C5, (**m**) C6, (**n**) C7, (**o**) C8, and (**p**) C9.

The morphologies of catalysts C1–C9 were examined by SEM (Figure 5). C1–C3 appeared as uniform rounded polyhedral sub-micron particles (0.2–1.0 μm) with a thin organic coating (Figure 5a–f). C4, C6, and C8 exhibited irregular mechanically broken particles with fine silica and alumina fragments on the surface (Figure 5g–i,m,o). In contrast, sol-gel-calcined catalysts C5, C7, and C9 displayed porous, interconnected sponge-like aggregates composed of primary nanoparticles fused into a high-surface-area matrix (Figure 5j–l,n,p). Notably, C7 exhibited the most uniform porous sponge-like morphology with well-developed interconnected networks (Figure 5n), providing maximum accessibility to active acid sites for catalytic chlorination. C5 showed a similar porous structure but without aluminum incorporation (Figure 5j–l), while physically doped catalysts lacked this porous architecture, explaining their inferior catalytic performance.

**Figure 6:**
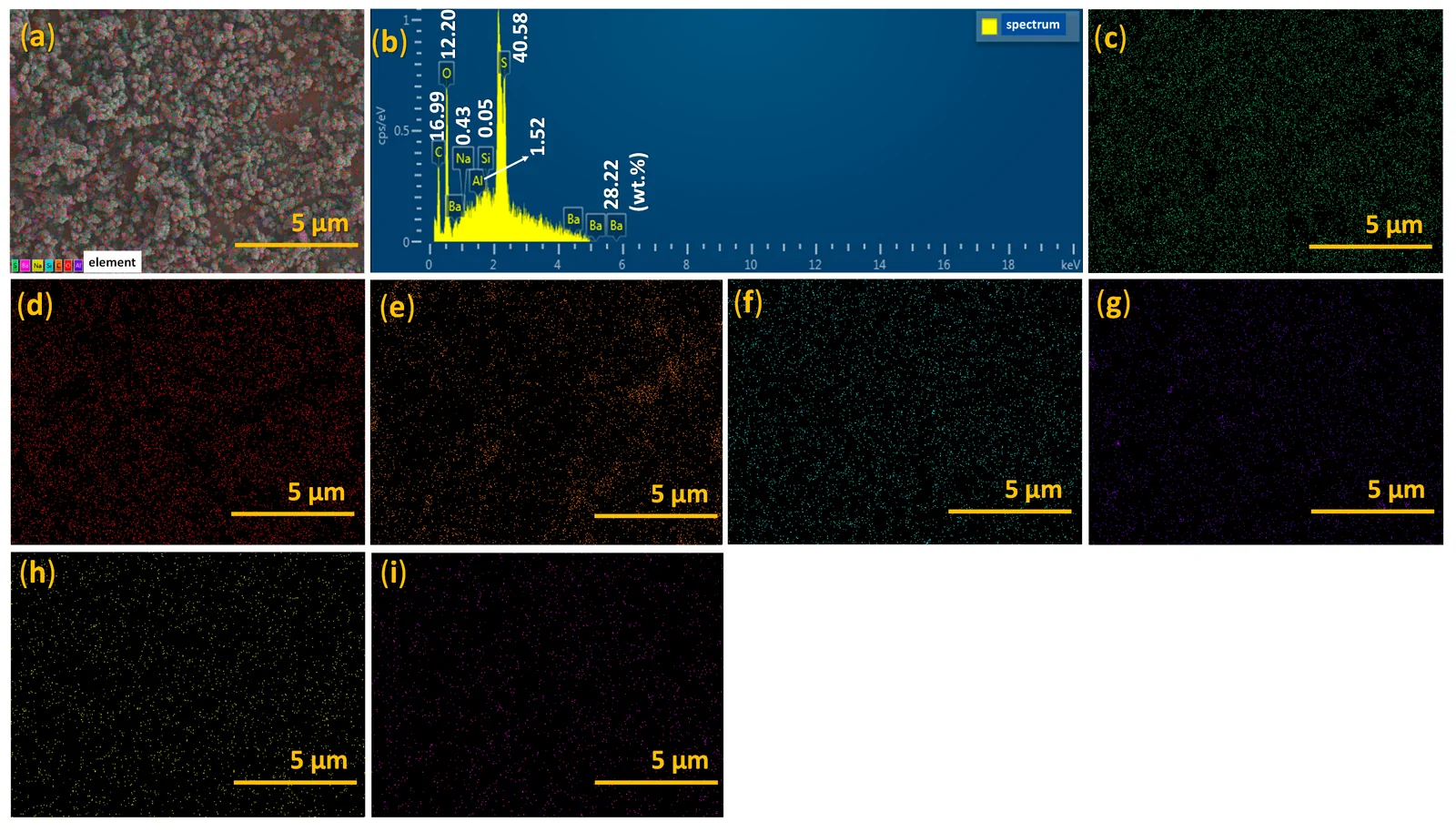
EDS mapping images of C7: (**a**) EDS layered image, (**b**) EDS spectrum, (**c**) S K*α*1, (**d**) O K*α*1, (**e**) C K*α*1,2, (**f**) Si K*α*1, (**g**) Al K*α*1, (**h**) Na K*α*1,2, and (**i**) Ba M*α*.

EDS elemental mapping confirmed the highly uniform spatial dispersion of S, O, C, Si, Al, Na, and Ba across the entire microstructure of all catalysts (Figure 6; Figures S8–S15). For C7, Figure 6 shows the homogeneous distribution of all constituent elements, confirming complete atomic-level integration achieved through the sol-gel/calcination process. The uniform dispersion of Al and Si in C7 is particularly significant, as it ensures maximum accessibility of Al–O–Si acid sites essential for electrophilic chlorination activity. Similarly, C5 exhibited uniform Si and Na distribution (Figure S12), while C1–C3 showed homogeneous C distribution from stearic acid coating (Figures S8–S10). Physically doped catalysts C4, C6, and C8 displayed uniform but less integrated elemental dispersion compared to sol-gel samples.

**Figure 7:**
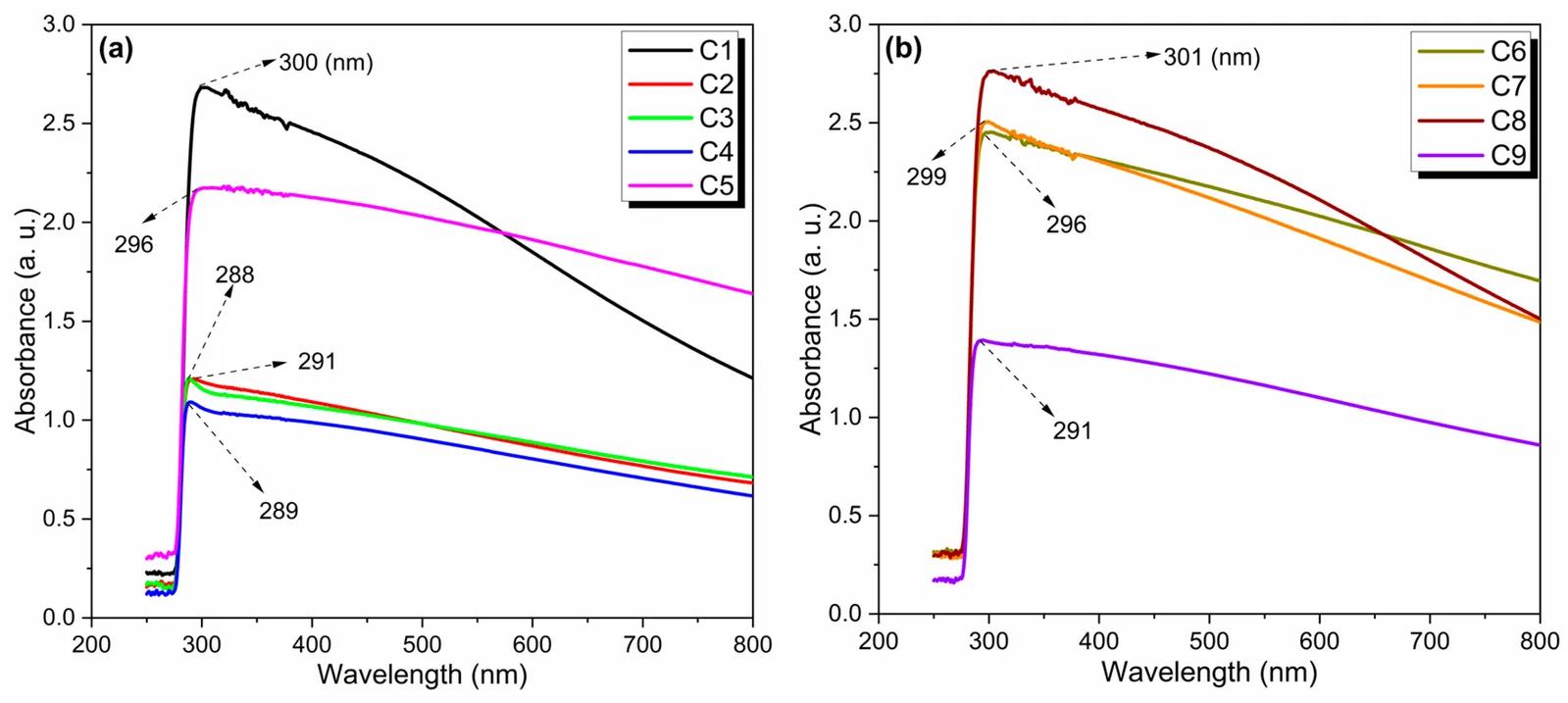
UV-Vis spectra of synthesized catalysts: (**a**) C1, C2, C3, C4, C5; (**b**) C6, C7, C8, C9.

UV-Vis absorption spectra of the catalysts are shown in Figure 7. The maximum absorption bands varied with modification strategy: C1 (300 nm), C2 (291 nm), and C3 (288 nm) for stearic-acid-coated catalysts, C4 (289 nm) and C5 (296 nm) for Na_2_SO_4_/SiO_2_-doped, C6 (296 nm) and C7 (299 nm) for Al/Si-containing, and C8 (301 nm, highest) and C9 (291 nm). These absorptions are attributed to ligand-to-metal charge transfer (LMCT) and interfacial electron interactions between the BaSO_4_ core and surface modifiers [54]. For C7, the absorption at 299 nm (Figure 7b) reflects the unique Al–O–Si electronic environment created by sol-gel processing, which correlates with enhanced acid site generation. C5 exhibited lower absorption (296 nm; Figure 7a) due to the absence of aluminum. The absorption wavelengths of C1–C3 systematically decreased with increasing stearic acid content, indicating organic modifier effects on surface electronic structure. Sol-gel catalysts showed varying absorption depending on Al/Si ratio and sodium content.

**Figure 8:**
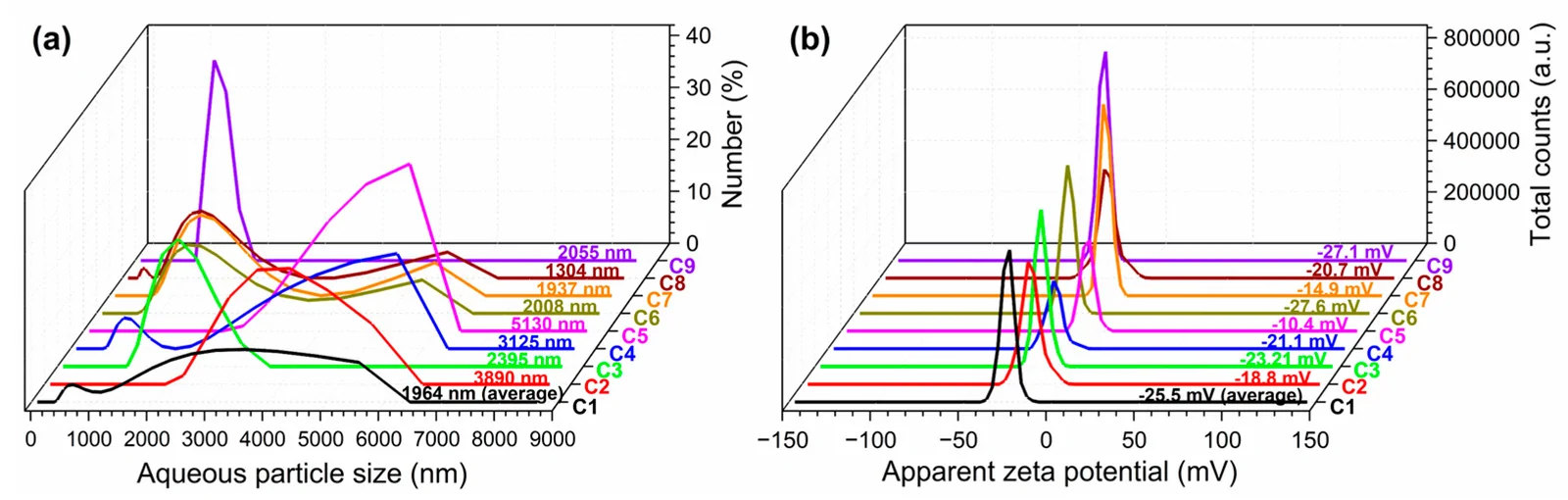
Aqueous particle sizes (**a**) and apparent zeta potentials (**b**) of synthesized catalysts.

Aqueous particle size (Figure 8a) revealed significant variations in particle sizes depending on the modification strategy. Stearic-acid-coated catalysts (C1–C3) and physically doped samples (C4, C6, and C8) exhibited sizes ranging from 1304 to 3890 nm, while sol-gel catalysts (C5, C7, and C9) showed the largest variations. All catalysts exhibited negative zeta potentials (Figure 8b), indicating stable colloidal dispersion in aqueous media. C7 showed moderate particle size (1937 nm) and zeta potential (−14.9 mV), balancing dispersion stability with surface accessibility for catalytic reactions. C5 exhibited the largest size (5230 nm) and lowest zeta potential (−10.4 mV), indicating significant agglomeration that reduces active site accessibility. C8 showed the smallest size (1304 nm) but lacked aluminum content essential for acid site generation. Stearic-acid-coated catalysts (C1–C3) displayed reduced surface charge due to organic coating layers, while sol-gel catalysts exhibited higher negative charges from sulfate and silicate surface groups [55].

**Figure 9:**
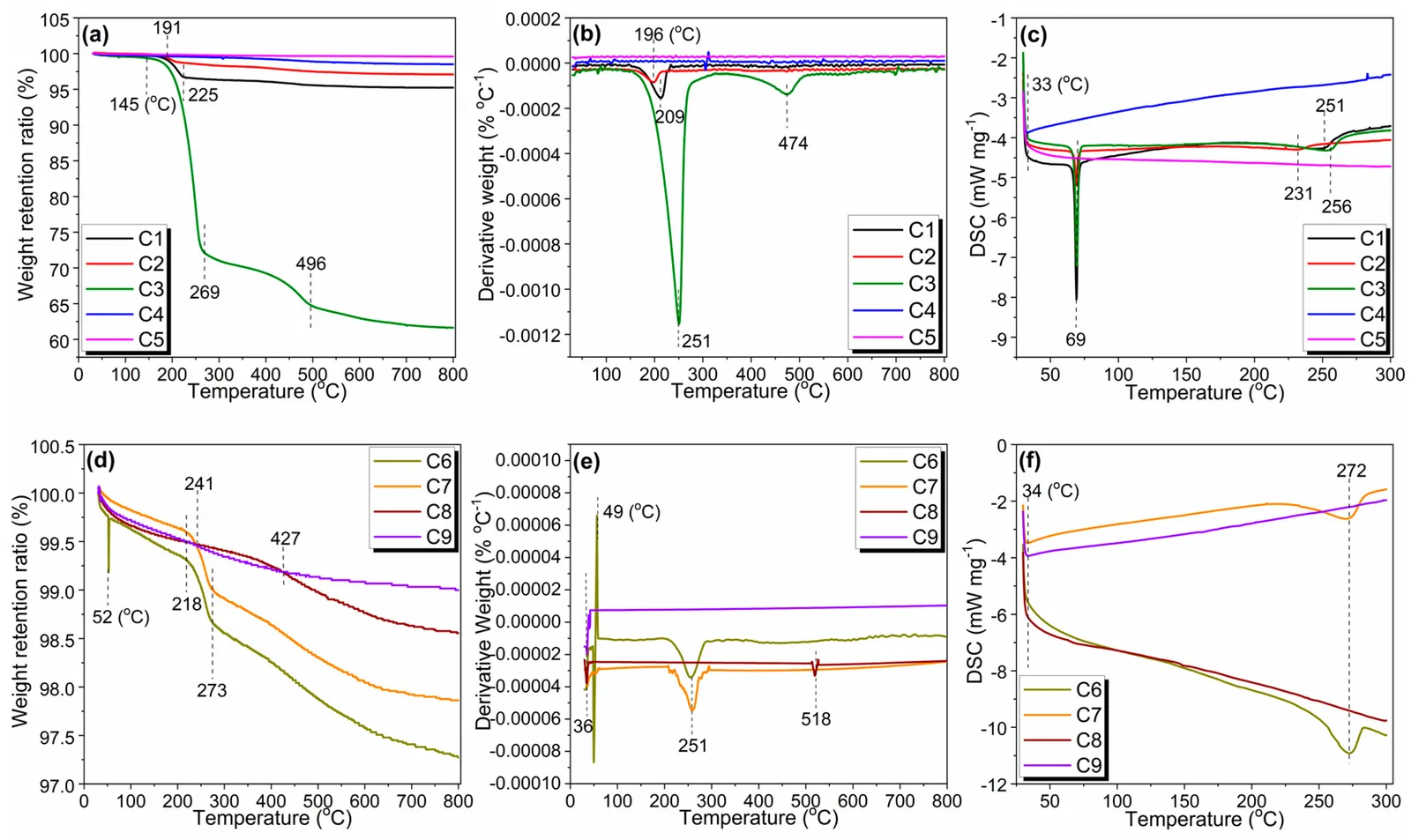
Thermal analysis of synthesized catalysts: (**a**) TGA curves of C1, C2, C3, C4, and C5; (**b**) DTG curves of C1, C2, C3, C4, and C5; (**c**) DSC curves of C1, C2, C3, C4, and C5; (**d**) TGA curves of C6, C7, C8, and C9; (**e**) DTG curves of C6, C7, C8, and C9; (**f**) DSC curves of C6, C7, C8, and C9.

Thermal stability was evaluated by TGA (Figure 9a,d) and DTG (Figure 9b,e) with a temperature range of 30–800 °C, as well as by DSC (Figure 9c,f) with a temperature range of 30–300 °C. Weight loss below 250 °C corresponds to desorption of physically adsorbed water and removal of surface hydroxyl groups, while weight loss above 250 °C indicates oxidative decomposition of organic species or structural reorganization of surface modifiers (Table S2). Stearic-acid-coated catalysts C1–C3 exhibited significant weight loss. C1 loses weight up to 3.48% below 250 °C and 1.29% above 250 °C (Entry 1, Table S2), C2 loses weight up to 1.45% below 250 °C and 1.46% above 250 °C (Entry 2, Table S2), and C3 loses weight up to 21.5% below 250 °C and 16.87% above 250 °C (Entry 3, Table S2) with exothermic peaks at 231–256 °C (Figure 9c), corresponding to oxidative combustion of the stearic acid organic coating. C3 showed the highest weight loss due to its highest stearic acid content (i.e., 3.83 g; Figure 1a), confirming that decomposition above 250 °C is primarily organic combustion.

In contrast, sol-gel-calcined catalysts demonstrated excellent thermal stability with total weight loss less than 2.5%, i.e., C5 (0.20%, 0.22%), C7 (0.66%, 1.48%), and C9 (0.57%, 0.44%; Entries 5, 7, and 9; Table S2). C7 showed minimal weight loss at both temperature ranges (Entry 7, Table S2), confirming the effectiveness of 550 °C calcination in stabilizing the inorganic aluminosilicate shell [29]. The small weight loss observed is attributed to residual surface hydroxyl groups, which are essential for Brønsted acid site generation (Si–OH–Al). Physically doped catalysts (C4, C6, and C8) also exhibited minimal weight loss (Entries 4, 6, and 8; Table S2), indicating good thermal stability due to their inorganic nature.

### 3.3. Catalytic Chlorination Performance

#### 3.3.1. Chlorination of Toluene

The catalytic performance of catalysts C1–C9 in the toluene chlorination reaction with hydrochloric acid as the chlorine donor was systematically evaluated (Table 1). The product distribution monitored by GC-MS (Figures S17–S44) includes 2-chlorotoluene (product A, ortho-position), 4-chlorotoluene (product B, para-position), benzyl chloride (product C, side-chain chlorination), 2,4-dichlorotoluene (product D), benzyl alcohol (product E, side-chain oxidation), and 2,4,5-trichlorotoluene (product F). The detailed GC-MS spectra of each item are shown in Figures S17–S44.

The effect of the type of catalyst (Entries 1–10, Table 1) on the conversion rate of toluene was observed with fixed amounts of toluene (2.0 mmol), HCl (8.0 mmol), H_2_O_2_ (2.0 mmol) as the oxidant, and temperature (60 °C). In the non-catalytic control experiment, the conversion rate of toluene was only 25.50% (Entry 1, Table 1). The introduction of the 20 mg of modified BaSO_4_ catalysts significantly promoted the reaction: the conversion rate of the stearic-acid-modified catalyst C1–C3 was 36.10–59.31% (Entries 2–4, Table 1). Sol-gel calcination catalysts C5 and C7 exhibited the highest catalytic activity in the H_2_O_2_ system, achieving conversion rates of 85.45% and 89.87%, respectively (Entries 6 and 8, Table 1), which were superior to the C4 (42.38%) and C6 (47.82%). The excellent activity of C7 stems from the synergistic Lewis acidity of tetrahedral Al^3+^ embedded in the porous BaSO_4_ matrix and the bridging Si–OH sites, which promotes the coordination and polarization of the HOCl/Cl_2_ intermediate. This performance enhancement is attributed to the high surface area, mesoporous network, and abundant surface Al–OH and Si–OH Lewis/Brønsted acidic sites formed during the sol-gel hybridization process.

The effect of the concentration of H_2_O_2_ (Entries 8, 11–14, Table 1; Figure 12b) on the conversion rate of toluene was observed with fixed amounts of C7 catalyst (20 mg), toluene (2.0 mmol), HCl (8.0 mmol), and temperature (60 °C). The 2.0 mmol amount of H_2_O_2_ oxidant gave the highest conversion rate of toluene, i.e., 89.87% (Entry 8, Table 1), while by other dosages of H_2_O_2_, i.e., 0.5–1.5 mmol and 2.5 mmol, only a conversion rate of 39.70–58.16% (Entries 11–13, Table 1) was achieved. Raising the temperature from 60 °C to 120 °C and keeping other conditions the same, i.e., fixed amounts of C7 catalyst (20 mg), toluene (2.0 mmol), HCl (8.0 mmol), and H_2_O_2_ (2.0 mmol) as an oxidant, led to a sharp drop in the conversion rate to 30.99% (Entries 15–17, Table 1), which was due to the accelerated thermal decomposition of H_2_O_2_ into O_2_ and H_2_O at high temperatures.

The mechanism of the H_2_O_2_-mediated toluene chlorination reaction (Figure S16) proceeds through a pathway similar to haloperoxidase: H_2_O_2_ oxidizes HCl to hypochlorous acid (HOCl), and HOCl reacts with additional HCl to form active electrophilic chlorine species (Cl^+^; Step 1, Figure S16), which generate 2-chlorotoluene (A), 4-chlorotoluene (B), and 2, 4-dichlorotoluene (D) through classical electrophilic aromatic substitution (EAS) (Step 2, Figure S16) [34].

It is notable that the reactivity increased sharply when H_2_O_2_ was replaced with ammonium persulfate ((NH_4_)_2_S_2_O_8_) as an oxidant (Entries 18–28, Table 1) [19]. Even in the blank reaction, (NH_4_)_2_S_2_O_8_ (1.0 mmol) as an oxidant achieved a conversion rate of 66.93% (Entry 18, Table 1). In the presence of (NH_4_)_2_S_2_O_8_ (1.0 mmol), HCl (8.0 mmol), and temperature (60 °C) with different catalysts (20 mg), i.e., C3, C5, and C7, the conversion rate achieved 76.46%, 88.94%, and 92.17%, respectively (Entries 19–21, Table 1).

The effect of the concentration of (NH_4_)_2_S_2_O_8_ (Entries 21–25, Table 1; Figure 12c) on the conversion rate of toluene was observed with fixed amounts of C7 (20 mg), toluene (2.0 mmol), HCl (8.0 mmol), and temperature (60 °C). The 2.0 mmol amount of (NH_4_)_2_S_2_O_8_ oxidant gave the highest conversion rate of toluene, i.e., 100.0% (Entry 24, Table 1). Higher reaction temperatures (80–120 °C) maintained a conversion rate of 94.88–100.0% (Entries 26–28, Table 1), while selectively turning to side-chain functionalization: benzyl chloride (C) reached 42.23% at 120 °C, and benzyl alcohol (E) reached 31.43% at 80 °C.

**Figure 10:**
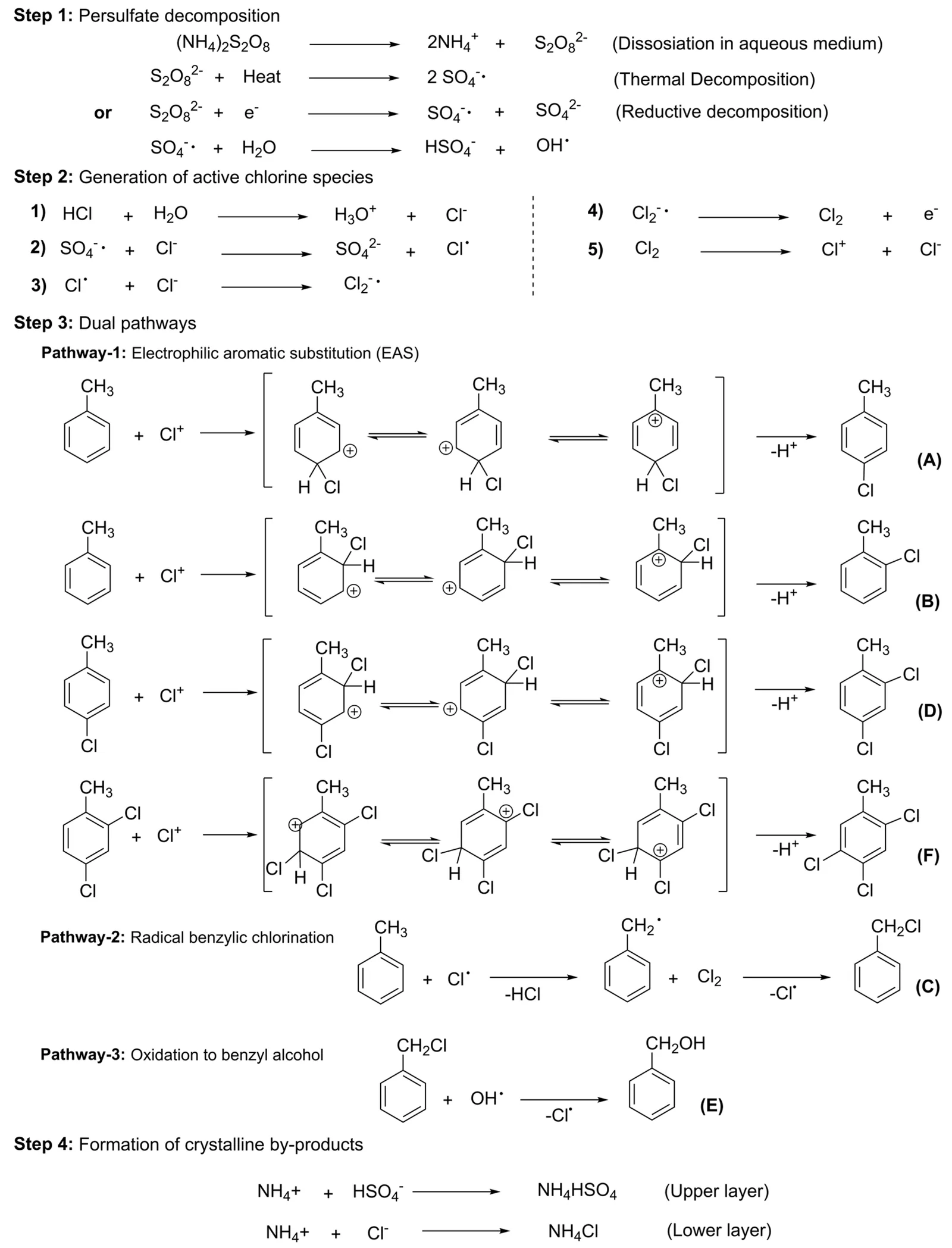
Proposed mechanism for the chlorination of toluene using (NH_4_)_2_S_2_O_8_ as an oxidant.

The persulfate reaction mechanism (Figure 10) involves the thermal/catalytic homolytic cleavage of S_2_O_8_^2−^ to form a sulfate radical anion (SO_4_^−^**∙**; Step 1, Figure 10), which rapidly oxidizes Cl^−^ to a chlorine radical (Cl**∙**) and a dichloride radical anion (Cl_2_^−^**∙**), thereby generating electrophilic species Cl^+^ and Cl_2_ (Step 2, Figure 10) [19]. At 60 °C, EAS on the ring is dominant, generating A and B (Pathway 1, Figure 10). At elevated temperatures, SO_4_^−^**∙** and Cl**∙** extract hydrogen atoms from methyl groups to form benzyl radicals (PhCH_2_**∙**), which in turn generate benzyl chlorine (C; Pathway 2, Figure 10) and oxidize to benzyl alcohol (E; Pathway 3, Figure 10). Here, in this mechanism crystalline by-products (NH_4_Cl and NH_4_HSO_4_) were also supposed to be formed in all (NH_4_)_2_S_2_O_8_-facilitated reactions (Step 4, Figure 10). This was observed in iodobenzene chlorination reactions (Figure S73), confirmed by their XRD analysis (Figure 13) and XPS (Table 5; Figure 14).

#### 3.3.2. Chlorination of Fluorobenzene

To explore the activity of the catalyst on deactivated aromatics, the fluorobenzene chlorination reaction was evaluated (Table 2). The products identified by GC-MS (Figures S46–S55) were 1-chloro-4-fluorobenzene (product A, para-position) and 1-chloro-2-fluorobenzene (product B, ortho-position).

Using H_2_O_2_ as the oxidant, the blank reaction at 60 °C only achieved a conversion rate of fluorobenzene to 10.50% (Entry 1, Table 2), while catalysts C3, C5, and C7 increased the conversion rates to 19.33%, 31.03%, and 51.35%, respectively (Entries 2–4, Table 2). Higher efficiency was achieved using (NH_4_)_2_S_2_O_8_: 24.28% for blank reaction (Entry 5, Table 2), 37.57% for C3 (Entry 6, Table 2), 47.82% for C5 (Entry 7, Table 2), and 51.74% for C7 at 60 °C (Entry 8, Table 2). When the temperature was raised to 80 °C, the conversion rate of C7 increased to 54.81% (Entry 9, Table 2). In all cases, 1-chloro-4-fluorobenzene (A) is the main product (>90% selectivity), which is due to the strong para-directed resonance effect and steric hindrance of the fluorine substituents. At 100 °C, the conversion rate dropped to 34.60% (Entry 10, Table 2), which was due to the enhanced competitive decomposition of the oxidant. The unified dual-pathway mechanism of fluorobenzene chlorination (Figure S45) details the H_2_O_2_ haloperoxidase pathway (Pathway 1a, Figure S45) and the (NH_4_)_2_S_2_O_8_ sulfate radical cascade pathway (Pathway 1b, Figure S45). Here, in this mechanism crystalline by-products (NH_4_Cl and NH_4_HSO_4_) were also supposed to be formed in all (NH_4_)_2_S_2_O_8_-facilitated reactions (Step 3, Figure S45). This was observed in iodobenzene chlorination reactions (Figure S73).

#### 3.3.3. Chlorination of Bromobenzene

The chlorination reaction of bromobenzene shows a competition between the electrophilic substitution of C–H on the ring and the cleavage of the C–Br bond (in situ substitution). The products identified by GC-MS (Figures S57–S72) included chlorobenzene (product A, in situ chlorination), 1-bromo-4-chlorobenzene (product B), 1-bromo-2-chlorobenzene (product C), 1,4-dichlorobenzene (product D), 1,2-dichlorobenzene (product E), 1,4-dibromobenzene (product F), and 1,2-dibromobenzene (product G).

The effect of the loading amount of catalyst on the conversion rate on bromobenzene was also evaluated (Entries 1–6 and 9–13, Table 3; Figure 12d,e). Under H_2_O_2_ activation, in the absence of catalyst, at 60 °C, conversion of bromobenzene was 13.68% (Entry 1, Table 3; Figure 12d). By varying the loading amount of C7 catalyst from 10 to 40 mg, chlorobenzene (A) was generated as the sole product through in situ dehalogenation reaction, with a conversion rate of 15.29–26.77% (Entries 2–5, Table 3; Figure 12d). When the catalyst loading amount was 50 mg, the conversion rate reached 42.28% (Entry 6, Table 3; Figure 12d), generating chlorobenzene (A, 8.02% combined isomer), 1-bromo-4-chlorobenzene (D, 8.76%), 1-bromo-2-chlorobenzene (E, 4.39%), 1,4-dibromobenzene (F, 16.03%), and 1,2-dibromobenzene (G, 5.05%). The H_2_O_2_-mediated bromobenzene chlorination mechanism (Figure S56) involves the formation of benzyne intermediate to form chlorobenzene (Step 3, Figure S56) and removed Br^−^ ion, which is converted into Br^+^ ion to facilitate electrophilic re-bromination (Step 4, Figure S56).

**Figure 11:**
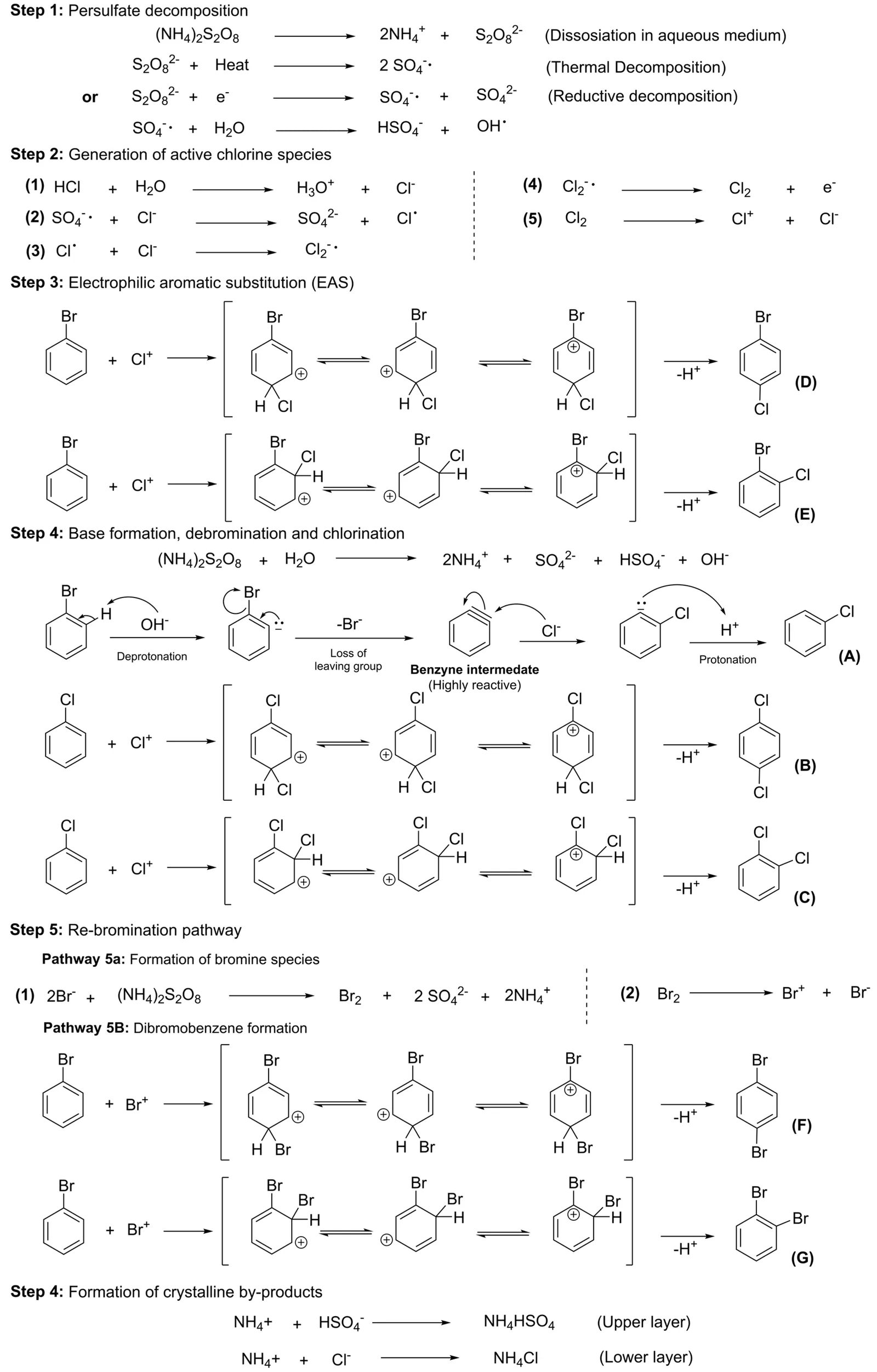
Proposed mechanism for the chlorination of bromobenzene using (NH_4_)_2_S_2_O_8_ as an oxidant.

**Figure 12:**
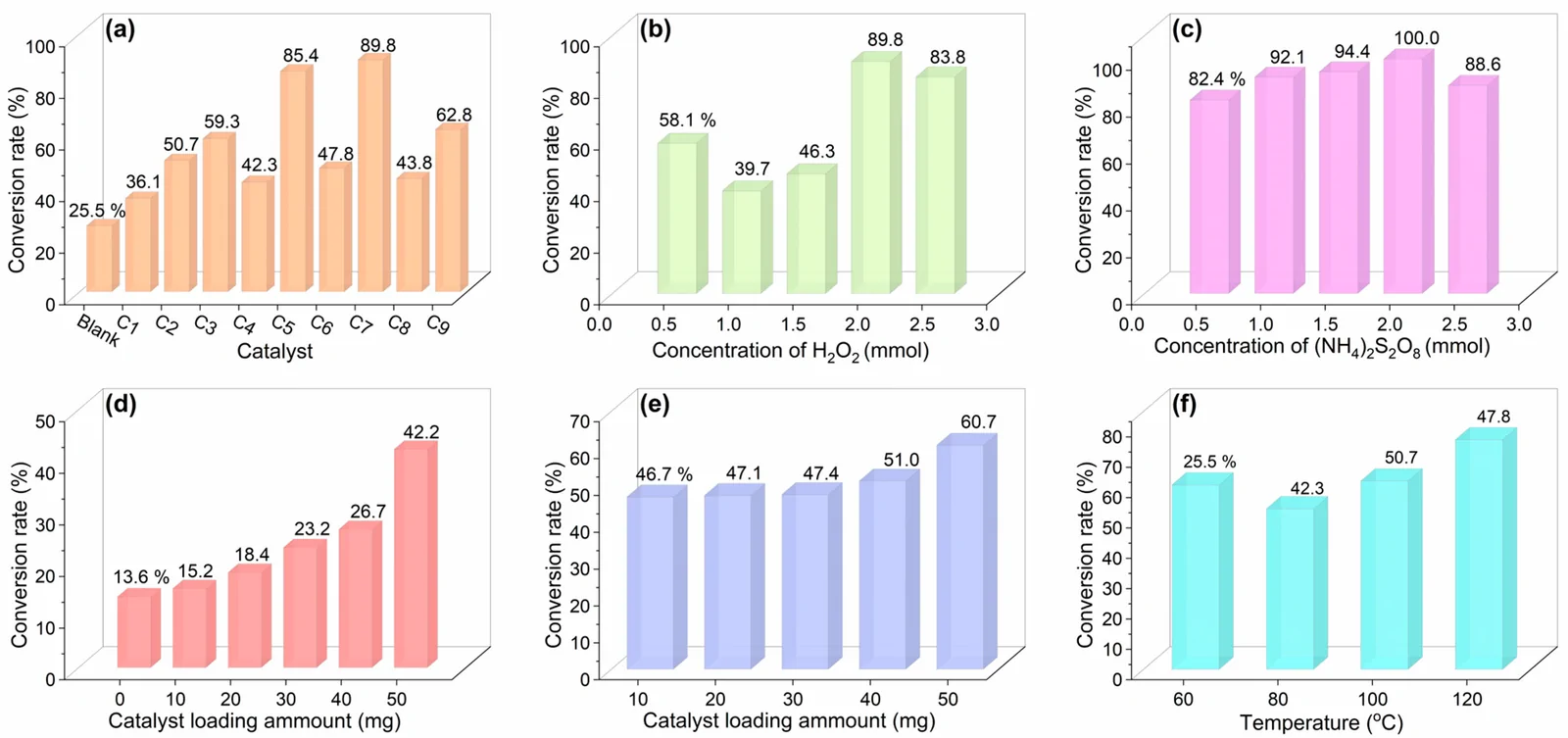
Catalytic performance of C7 under various reaction parameters: (**a**) Effect of the type of catalyst on the rate of conversion of toluene. Reaction conditions: toluene (2.0 mmol), catalyst (20 mg), oxidant H_2_O_2_ (aqueous, 30 wt.%, 2 mmol), and HCl (aqueous, 37 wt.%, 8 mmol pure HCl), 60 °C, reflux for 6 h (Entries 1–10, Table 1; Figures S17–S26, Supplementary Materials). (**b**) Effect of the concentration of H_2_O_2_ on the rate of conversion of toluene. Reaction conditions: toluene (2.0 mmol), catalyst C7 (20 mg), oxidant H_2_O_2_ (aqueous, 30 wt.%, 0.5~2.5 mmol), and HCl (aqueous, 37 wt.%, 8 mmol pure HCl), 60 °C, reflux for 6 h (Entries 8 and 11–14, Table 1; Figures S27–S30, Supplementary Materials). (**c**) Effect of the concentration of (NH_4_)_2_S_2_O_8_ on the rate of conversion of toluene. Reaction conditions: toluene (2.0 mmol), catalyst C7 (20 mg), oxidant (NH_4_)_2_S_2_O_8_ (aqueous solution, 37 wt.%, 0.5~2.5 mmol), and HCl (aqueous, 37 wt.%, 8 mmol pure HCl), 60 °C, reflux for 6 h (Entries 21–25, Table 1; Figures S37–S40, Supplementary Materials). (**d**) Effect of the amount of catalyst loading on the rate of conversion of bromobenzene in the presence of H_2_O_2_. Reaction conditions: bromobenzene (2 mmol), catalyst C7 (10~50 mg), oxidant H_2_O_2_ (aqueous, 30 wt.%, 2 mmol, and HCl (aqueous, 37 wt.%, 8 mmol pure HCl), 60 °C, reflux for 6 h (Entries 1–6, Table 3; Figures S57–S62, Supplementary Materials). (**e**) Effect of the amount of catalyst loading on the rate of conversion of bromobenzene in the presence of (NH_4_)_2_S_2_O_8_. Reaction conditions: bromobenzene (2 mmol), catalyst C7 (10~50 mg), oxidant (NH_4_)_2_S_2_O_8_ (aqueous solution, 37 wt.%, 2 mmol), and HCl (aqueous, 37 wt.%, 8 mmol pure HCl), 60 °C, reflux for 6 h (Entries 9–13, Table 3; Figures S65–S69, Supplementary Materials). (**f**) Effect of temperature on the rate of conversion of bromobenzene. Reaction conditions: bromobenzene (2 mmol), catalyst C7 (50 mg), oxidant (NH_4_)_2_S_2_O_8_ (aqueous solution, 37 wt.%, 2 mmol), and HCl (aqueous, 37 wt.%, 8 mmol pure HCl), 60~120 °C, reflux for 6 h (Entries 13–16, Table 3; Figures S69–S72, Supplementary Materials).

**Figure 13:**
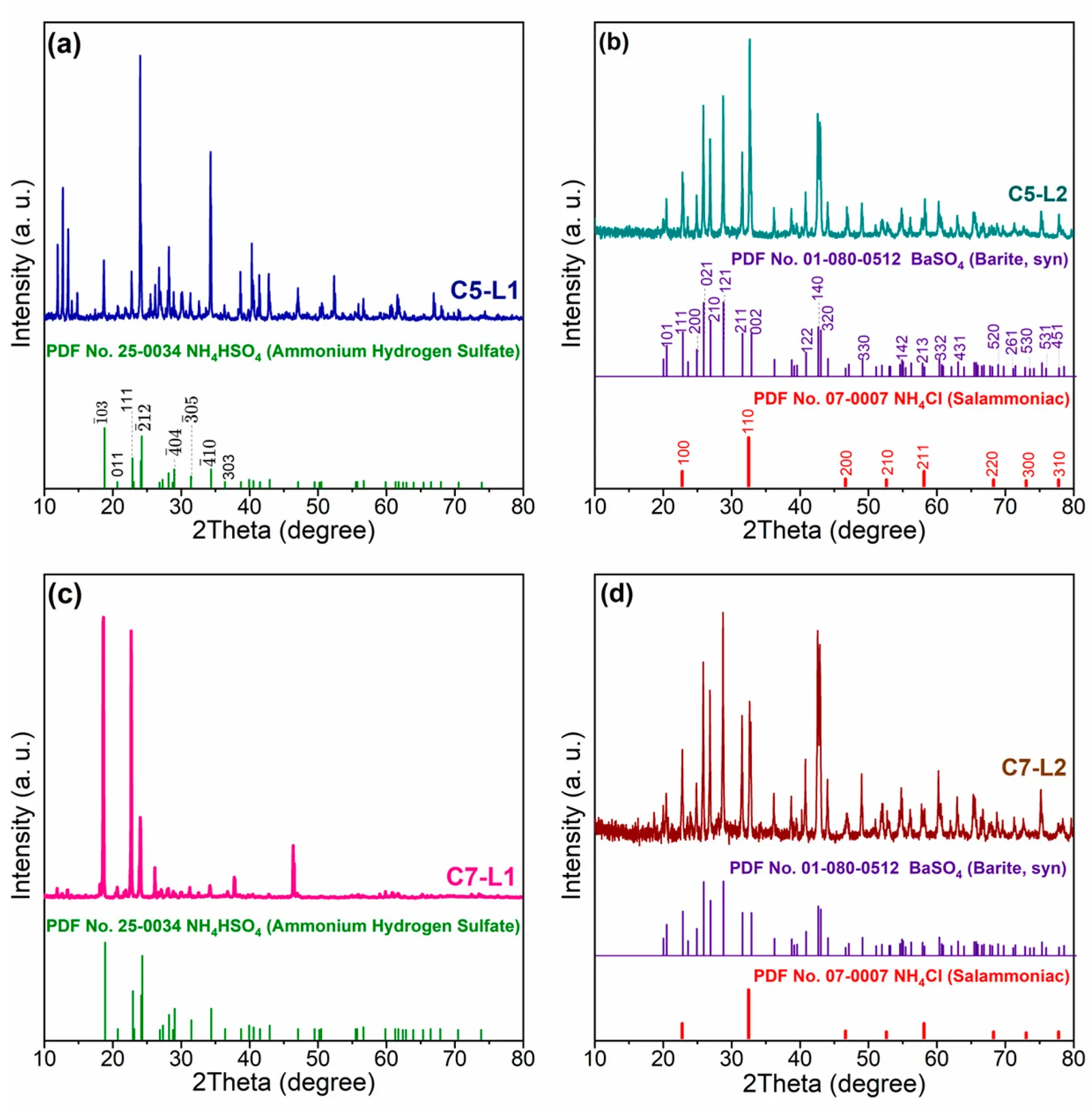
XRD patterns of crystalline by-products obtained from the chlorination of iodobenzene using (NH_4_)_2_S_2_O_8_ as an oxidant: (**a**) upper-layer crystals (C5-L1) from C5-facilitated reaction showing NH_4_HSO_4_ (Entry 8, Table 4); (**b**) lower-layer crystals (C5-L2) from C5-facilitated reaction showing BaSO_4_ with minor NH_4_Cl (Entry 8, Table 4); (**c**) upper-layer crystals (C7-L1) from C7-facilitated reaction showing NH_4_HSO_4_ (Entry 9, Table 4); (**d**) lower-layer crystals (C7-L2) from C7-facilitated reaction showing BaSO_4_ with minor NH_4_Cl (Entry 9, Table 4).

**Figure 14:**
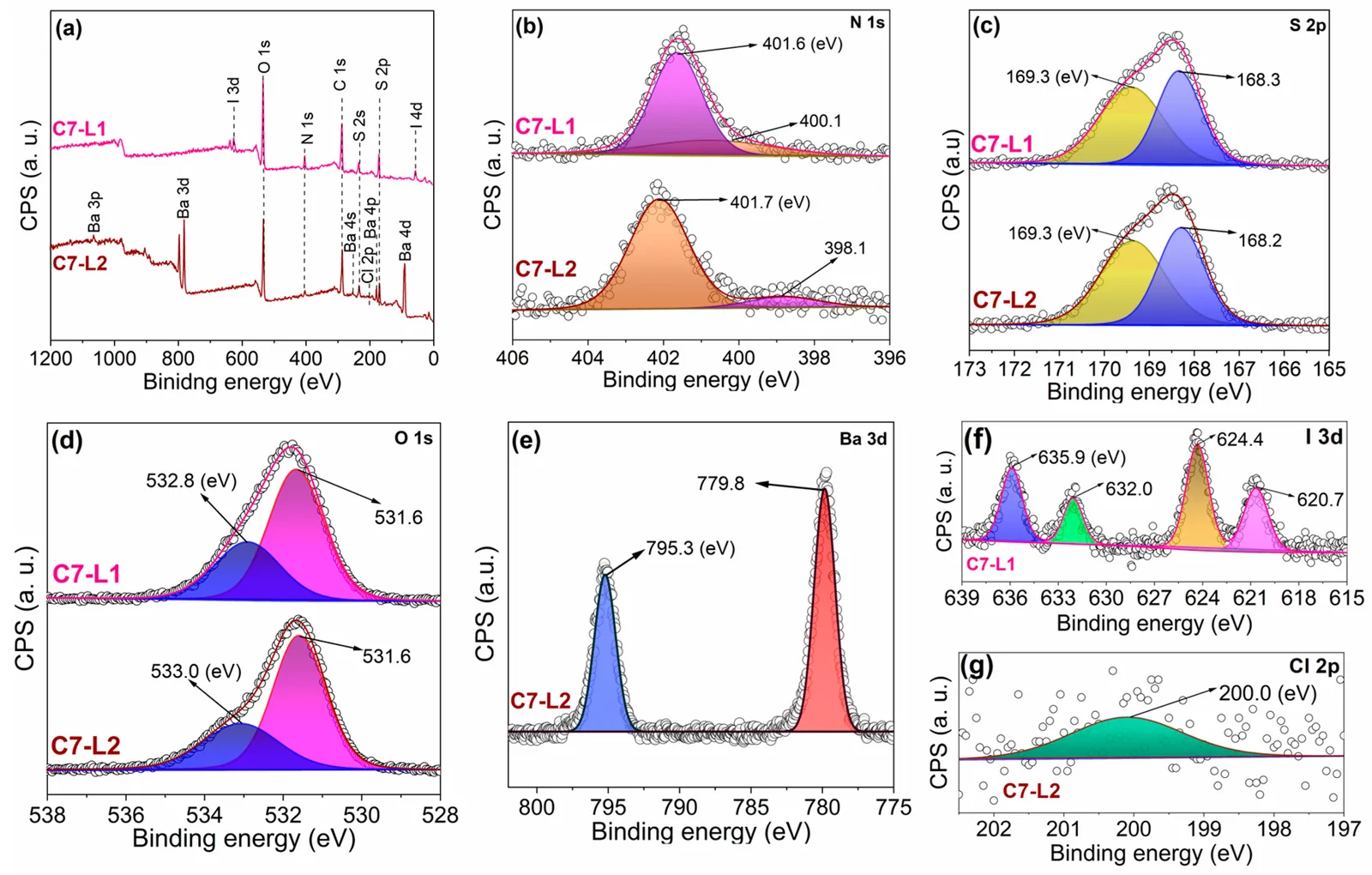
XPS analysis of upper-layer crystals (C7-L1) and lower-layer crystals (C7-L2): (**a**) XPS full-spectrum scan, (**b**) N 1s, (**c**) S2p, (**d**) O 1s, (**e**) Ba 3d, (**f**) I 3d, and (**g**) Cl 2p.

When (NH_4_)_2_S_2_O_8_ was used as the oxidant, the effect of the loading amount of C7 on the conversion rate of bromobenzene was observed (Entries 9–16, Table 3; Figure 12e). The conversion rate increased to 46.72–60.78% when the amount of catalyst increased from 10 to 50 mg, at 60 °C. The effect of temperature on the conversion rate of bromobenzene was also observed (Entries 13–16, Table 3; Figure 12f) with 50 mg of C7. The conversion rate reached 75.66% at 120 °C (Entry 16, Table 3; Figure 12f). The (NH_4_)_2_S_2_O_8_-mediated bromobenzene chlorination mechanism (Figure 11) involves the formation of benzyne intermediate to form chlorobenzene (Step 4, Figure 11) and removed Br^−^ ion, which is converted into Br^+^ ion to facilitate electrophilic re-bromination into products F and G (Step 5, Figure 11). At 120 °C, they were as high as 32.92% and 12.48%, respectively (Entry 16, Table 3). Here, in this mechanism crystalline by-products (NH_4_Cl and NH_4_HSO_4_) were also supposed to be formed in all (NH_4_)_2_S_2_O_8_-facilitated reactions (Step 4, Figure S11). This was observed in iodobenzene chlorination reactions (Figure S73), confirmed by their XRD analysis (Figure 13) and XPS (Table 5; Figure 14).

Finally, the effects of different parameters of reactions are summarized in Figure 12. Catalyst screening (Figure 12a) indicates that sol-gel calcination of C5 and C7 has a higher conversion rate. The varying amount of the concentration of H_2_O_2_ (Figure 12b) shows that the optimal concentration is 2.0 mmol. (NH_4_)_2_S_2_O_8_ concentration variation (Figure 12c) shows that the toluene conversion rate increases with the increase of persulfate, reaching 100% at 2.0 mmol. The catalyst loading amount in the bromobenzene reaction (Figure 12d,e) shows that as the C7 dosage increases from 10 mg to 50 mg, the conversion rate of the H_2_O_2_ system monotonically increases from 15.29% to 42.28%, and that of the persulfate system increases from 46.72% to 60.78%. The temperature effect in the bromobenzene reaction (Figure 12f) shows that in the (NH_4_)_2_S_2_O_8_ system, when the temperature rises from 60 °C to 120 °C, the conversion rate increases to 75.66% and is conducive to the formation of polyhalogenated products.

#### 3.3.4. Chlorination of Iodobenzene and Characterization of Crystlline By-Products

The chlorination reaction of iodobenzene was systematically studied (Table 4). The products identified by GC-MS (Figures S74–S82) included chlorobenzene (product A, in situ substitution), 1-chloro-4-iodobenzene (product B, para-position), and 1-chloro-2-iodobenzene (product C, ortho-position).

Using H_2_O_2_ as an oxidant, the conversion rate of iodobenzene without catalyst and with C7 catalyst at 60 °C was 7.94% and 16.54%, respectively. There was not any in situ chlorobenzene generated (Entries 1 and 2, Table 4). Using (NH_4_)_2_S_2_O_8_ as an oxidant, the conversion rate of iodobenzene at 120 °C and with different types of catalysts was increased from 6.75% of the blank reaction (Entry 3, Table 4) to 46.35% of C7 (Entry 9, Table 4), generating 5.87% chlorobenzene (A), 24.99% 1-chloro-4-iodobenzene (B), and 15.54% 1-chloro-2-iodobenzene (C). The catalytic activity sequence is as follows: blank < C1 < C4 < C8 < C3 < C5 < C7. The relatively weak C–I bond dissociation energy (222 kJ mol^−1^) is conducive to the in situ cleavage of free radicals under high-temperature persulfate activation. Figure S73 details the complete mechanisms of the H_2_O_2_ and persulfate pathways.

During the post-treatment chlorination of iodobenzene with (NH_4_)_2_S_2_O_8_ (Entries 8 and 9, Table 4), two different types of crystals were observed in the organic phase after standing at room temperature (Figure 13). Wide-angle XRD analysis indicated that the upper-layer crystals, C5-L1 and C7-L1 (Figure 13a,c), were pure crystalline ammonium hydrogen sulfate (NH_4_HSO_4_, PDF No. 25-0034), confirming the chemical reduction of persulfate. The lower crystals (Figure 13b,d) were mainly BaSO_4_ (PDF No. 01-080-0512), accompanied by a small amount of ammonium chloride (NH_4_Cl, PDF No. 07-0007; Step 4, Figure 11). This discovery confirmed the clean transformation of persulfate and the structural stability of the BaSO_4_ catalyst. The formation of these crystalline by-products further demonstrated the versatility of persulfate chemistry in generating recyclable and characterizable inorganic salts.

XPS analysis was performed on upper-layer crystals (C7-L1) and lower-layer crystals (C7-L2) from the C7-facilitated reaction (Entry 9, Table 4). Results are summarized in Table 5 and Figure 14.

XPS analysis of C7-L1 revealed O, C, N, S, and I as major elements (Entry 1, Table 5; Figure 14a) [44]. N 1s peaks at 401.6 eV and 400.1 eV confirm NH_4_^+^ (Figure 14b). The S 2p doublet at 169.3 and 168.3 eV confirms SO_4_^−^ (Figure 14c) [48]. O 1s peaks at 532.8 and 531.6 eV correspond to sulfate oxygen (Figure 14d). I 3d signals (Figure 14f) confirm iodine-containing organic products (1-chloro-4-iodobenzene and 1-chloro-2-iodobenzene) that crystallized during organic layer drying. Results confirm NH_4_HSO_4_ with iodobenzene products, consistent with XRD (PDF No. 25-0034).

XPS of C7-L2 revealed Ba, S, O, C, N, and Cl (Figure 14a). N 1s at 401.7 and 398.1 eV confirms NH_4_^+^ (Figure 14b). S 2p at 169.3 and 168.2 eV confirms SO_4_^2−^ (Figure 14c) [49]. O 1s peaks at 532.8 and 531.6 eV correspond to sulfate oxygen (Figure 14d). The Ba 3d doublet at 779.8 and 795.3 eV confirms Ba^2+^ in BaSO_4_ (Figure 14e) [46,47]. Cl 2p at ~200.0 eV confirms chloride (Figure 14g) in NH_4_Cl. These results confirm BaSO_4_ with minor NH_4_Cl, consistent with XRD (PDF No. 01-080-0512 and 07-0007).

The C 1s peak observed in both C7-L1 and C7-L2 crystals at 284.8 eV (Table 4) is attributed to adventitious surface carbon used for binding energy calibration. Since the samples were exposed to air during handling, this carbon signal is unavoidable and does not represent any crystalline phase. Therefore, no further analysis of the C 1s region is required for crystalline by-products.

## 4. Conclusions

This study establishes a modified barium sulfate (BaSO_4_) catalyst system for the chlorination of aromatic compounds, employing hydrochloric acid (HCl) as the chlorine source and either hydrogen peroxide (H_2_O_2_) or ammonium persulfate ((NH_4_)_2_S_2_O_8_) as the oxidant. Among the nine catalysts synthesized via stearic acid coating, physical doping, and sol-gel processing, the sol-gel-derived C7 exhibits superior performance. This enhanced activity is attributed to the well-dispersed Al–O–Si species, tetrahedrally coordinated Al^3+^, abundant Brønsted acid sites (Si–OH–Al), and uniform elemental dispersion on the porous morphology, which collectively facilitate HCl activation. Under optimized conditions, C7 achieves complete toluene conversion and demonstrates high conversions for fluorobenzene (55%), bromobenzene (76%), and iodobenzene (46%).

Mechanistically, H_2_O_2_ proceeds through a haloperoxidase-like pathway generating hypochlorous acid (HOCl) for electrophilic substitution. In contrast, ammonium persulfate initiates a radical cascade involving sulfate radical anions (SO_4_•^−^) that oxidize Cl^−^ to chlorine radicals (Cl•). Critically, the persulfate system enables an in situ halogen exchange pathway with bromobenzene and iodobenzene via benzyne intermediates and radical-mediated C–Br/C–I bond cleavage, yielding chlorobenzene as the exclusive product—a distinctive advantage over conventional methods.

XRD and XPS analyses of the crystalline by-products confirm the formation of NH_4_HSO_4_ and NH_4_Cl, validating the persulfate-driven radical mechanism, while the preservation of BaSO_4_ diffraction peaks attests to the catalyst’s structural integrity and reusability potential. Reaction optimization reveals a temperature-dependent selectivity profile: moderate temperatures (60 °C) favor ring chlorination, whereas elevated temperatures (80–120 °C) promote side-chain functionalization and polyhalogenation.

Unlike traditional protocols that rely on toxic molecular chlorine, noble metals, or harsh conditions, this work offers a sustainable and cost-effective route utilizing abundant BaSO_4_ supports and industrial HCl waste. The synergy between tailored surface acidity and persulfate-driven radical chemistry provides a versatile platform for tunable aromatic halogenation. Collectively, these findings not only deepen the fundamental understanding of persulfate-mediated chlorination mechanisms but also provide practical guidelines for designing robust heterogeneous catalysts for pharmaceutical, agrochemical, and fine chemical manufacturing, addressing both economic and environmental challenges inherent in conventional halogenation processes.

## Figures and Tables

**Table 1:** Chlorination of toluene.

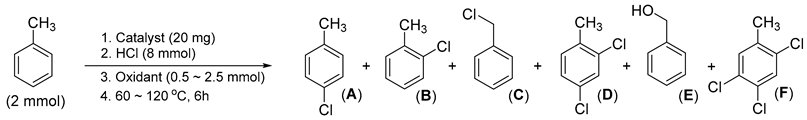											
Entry ^a^	Catalyst (20 mg)	Oxidant H_2_O_2_ (mmol)	Oxidant (NH_4_)_2_S_2_O_8_ (mmol)	T (°C)	Conversion (%) ^b^	A (%) ^c^	B (%) ^c^	C (%) ^c^	D (%) ^c^	E (%) ^c^	F (%) ^c^
1	Blank	2.0	-	60	25.50	16.32	7.90	-	1.23	-	-
2	C1	2.0	-	60	36.10	22.86	12.21	-	1.02	-	-
3	C2	2.0	-	60	50.78	31.38	15.17	-	4.22	-	-
4	C3	2.0	-	60	59.31	38.10	16.83	-	4.38	-	-
5	C4	2.0	-	60	42.38	27.78	13.53	-	1.05	-	-
6	C5	2.0	-	60	85.45	49.49	25.16	-	10.78	-	-
7	C6	2.0	-	60	47.82	29.28	14.47	-	4.06	-	-
8	C7	2.0	-	60	89.87	46.27	21.87	-	21.72	-	-
9	C8	2.0	-	60	43.82	27.61	15.69	-	0.51	-	-
10	C9	2.0	-	60	62.81	40.01	17.23	-	5.55	-	-
11	C7	0.5	-	60	58.16	37.85	18.92	-	1.36	-	-
12	C7	1.0	-	60	39.70	25.19	13.48	-	1.01	-	-
13	C7	1.5		60	46.34	28.51	16.50	-	1.32	-	-
14	C7	2.5	-	60	83.86	50.87	16.27	-	16.71	-	-
15	C7	2.0		80	32.18	23.48	8.69	-	-	-	-
16	C7	2.0		100	45.35	30.84	14.50	-	-	-	-
17	C7	2.0		120	30.99	25.26	5.73	-	-	-	-
18	Blank	-	1.0	60	66.93	30.98	24.71	6.75	4.48	-	-
19	C3	-	1.0	60	76.46	37.17	18.39	15.03	5.85	-	-
20	C5	-	1.0	60	88.94	39.80	17.71	18.22	7.68	1.39	4.11
21	C7	-	1.0	60	92.17	52.23	25.97	8.43	5.53	-	-
22	C7	-	0.5	60	82.43	26.11	17.39	31.15	7.76	-	-
23	C7	-	1.5	60	94.41	56.33	27.83	7.59	2.65	-	-
24	C7	-	2	60	100	42.84	20.40	25.56	11.180	-	-
25	C7	-	2.5	60	88.63	51.53	26.55	6.30	4.23	-	-
26	C7	-	2.0	80	97.23	23.40	18.37	18.12	5.893	31.43	-
27	C7	-	2.0	100	94.88	19.72	12.81	22.69	10.65	28.98	-
28	C7	-	2.0	120	100	16.54	10.25	42.23	14.31	16.65	-

^a^ Reaction conditions: toluene (2.0 mmol), catalyst (20 mg), oxidant H_2_O_2_ (aqueous, 30 wt.%, 0.5~2.5 mmol) or oxidant (NH_4_)_2_S_2_O_8_ (aqueous solution, 37 wt.%, 0.5~2.5 mmol), and HCl (aqueous, 37 wt.%, 8.0 mmol pure HCl), 60~120 °C, reflux for 6 h. ^b^ Conversion of toluene (%), determined by GC-MS (Figures S17–S44, Supplementary Materials), calculated by area normalization using the following formula: 1—[integrated area of residual toluene]/([integrated area of residual toluene] + [integrated area of all chlorinated products]) × 100%. ^c^ Yield of chlorinated product (%), determined by GC-MS (Figures S17–S44, Supplementary Materials), calculated by area normalization using the following formula: [integrated area of target chlorinated product]/([integrated area of residual toluene] + [integrated area of all chlorinated products]) × 100%.

**Table 2:** Chlorination of fluorobenzene.

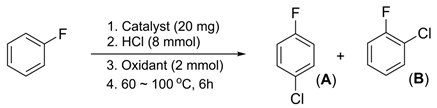						
Entry ^a^	Catalyst (20 mg)	Oxidant (2.0 mmol)	T (°C)	Conversion (%) ^b^	A (%) ^c^	B (%) ^c^
1	Blank	H_2_O_2_	60	10.50	10.50	-
2	C3	H_2_O_2_	60	19.33	19.33	-
3	C5	H_2_O_2_	60	31.03	28.86	2.16
4	C7	H_2_O_2_	60	51.35	47.07	4.28
5	Blank	(NH_4_)_2_S_2_O_8_	60	24.28	24.28	-
6	C3	(NH_4_)_2_S_2_O_8_	60	37.57	34.23	3.32
7	C5	(NH_4_)_2_S_2_O_8_	60	47.82	41.49	6.33
8	C7	(NH_4_)_2_S_2_O_8_	60	51.74	47.33	4.40
9	C7	(NH_4_)_2_S_2_O_8_	80	54.81	49.45	5.35
10	C7	(NH_4_)_2_S_2_O_8_	100	34.60	34.60	-

^a^ Reaction conditions: fluorobenzene (2.0 mmol), catalyst (20 mg), oxidant H_2_O_2_ (aqueous, 30 wt.%, 2.0 mmol) or oxidant (NH_4_)_2_S_2_O_8_ (aqueous solution, 37 wt.%, 2 mmol), and HCl (aqueous, 37 wt.%, 8.0 mmol pure HCl), 60~100 °C, reflux for 6 h. ^b^ Conversion of fluorobenzene (%), determined by GC-MS (Figures S46–S55, Supplementary Materials), calculated by area normalization using the following formula: 1—[integrated area of residual fluorobenzene]/([integrated area of residual fluorobenzene] + [integrated area of all chlorinated products]) × 100%. ^c^ Yield of chlorinated product (%), determined by GC-MS (Figures S46–S55, Supplementary Materials), calculated by area normalization using the following formula: [integrated area of target chlorinated product]/([integrated area of residual fluorobenzene] + [integrated area of all chlorinated products]) × 100%.

**Table 3:** Chlorination of bromobenzene.

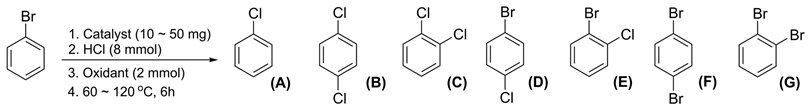											
Entry ^a^	C7 Catalyst Loading (mg)	Oxidant (2 mmol)	T (°C)	Conv. (%) ^b^	A (%) ^c^	B (%) ^c^	C (%) ^c^	D (%) ^c^	E (%) ^c^	F (%) ^c^	G (%) ^c^
1	Blank	H_2_O_2_	60	13.68	13.68	-	-	-	-	-	-
2	10	H_2_O_2_	60	15.29	15.29	-	-	-	-	-	-
3	20	H_2_O_2_	60	18.42	18.42	-	-	-	-	-	-
4	30	H_2_O_2_	60	23.24	23.24	-	-	-	-	-	-
5	40	H_2_O_2_	60	26.77	26.77	-	-	-	-	-	-
6	50	H_2_O_2_	60	42.28	8.02 ^d^	-	-	8.76	4.39	16.03	5.05
7	50	H_2_O_2_	80	25.28	17.63	-	-	-	-	5.99	1.65
8	50	H_2_O_2_	120	12.56	4.81	-	-	-	-	5.86	1.88
9	10	(NH_4_)_2_S_2_O_8_	60	46.72	13.37	1.60	0.76	9.22	6.10	12.68	2.96
10	20	(NH_4_)_2_S_2_O_8_	60	47.12	12.69	2.29	1.70	11.28	8.35	8.94	1.84
11	30	(NH_4_)_2_S_2_O_8_	60	47.40	13.07	1.75	0.97	11.72	7.23	10.76	1.87
12	40	(NH_4_)_2_S_2_O_8_	60	51.08	12.81	2.88	2.99	12.28	8.57	9.73	1.79
13	50	(NH_4_)_2_S_2_O_8_	60	60.78	18.08	3.29	2.25	13.77	8.57	12.03	2.77
14	50	(NH_4_)_2_S_2_O_8_	80	52.89	34.55	-	-	7.18	3.06	6.55	1.54
15	50	(NH_4_)_2_S_2_O_8_	100	62.09	3.87	-	-	14.40	5.72	28.69	9.40
16	50	(NH_4_)_2_S_2_O_8_	120	75.66	9.62	-	-	16.50	4.11	32.92	12.48

^a^ Reaction conditions: bromobenzene (2.0 mmol), catalyst (10~50 mg), oxidant H_2_O_2_ (aqueous, 30 wt.%, 2.0 mmol) or oxidant (NH_4_)_2_S_2_O_8_ (aqueous solution, 37 wt.%, 2.0 mmol), and HCl (aqueous, 37 wt.%, 8.0 mmol pure HCl), 60~120 °C, reflux for 6 h. ^b^ Conversion of fluorobenzene (%), determined by GC-MS (Figures S57–S72, Supplementary Materials), calculated by area normalization using the following formula: 1—[integrated area of residual bromobenzene]/([integrated area of residual bromobenzene] + [integrated area of all chlorinated products]) × 100%. ^c^ Yield of chlorinated product (%), determined by GC-MS (Figures S57–S72, Supplementary Materials), calculated by area normalization using the following formula: [integrated area of target chlorinated product]/([integrated area of residual bromobenzene] + [integrated area of all chlorinated products]) × 100%. ^d^ Yield of product A for entry 6 is the sum of % yield of two peaks of the GC-MS graph, indicating the same type of product, i.e., 3.48 + 4.54 = 8.02 (Figure S62, Supplementary Materials).

**Table 4:** Chlorination of iodobenzene.

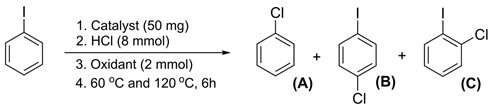							
Entry ^a^	Catalyst (50 mg)	Oxidant (2.0 mmol)	T (°C)	Conversion (%) ^b^	A (%) ^c^	B (%) ^c^	C (%) ^c^
1	Blank	H_2_O_2_	60	7.94	-	4.33	3.60
2	C7	H_2_O_2_	60	16.54	-	7.94	8.60
3	Blank	(NH_4_)_2_S_2_O_8_	120	6.75	2.92	2.12	1.70
4	C1	(NH_4_)_2_S_2_O_8_	120	8.43	2.56	2.55	3.31
5	C4	(NH_4_)_2_S_2_O_8_	120	11.06	2.25	5.00	3.79
6	C8	(NH_4_)_2_S_2_O_8_	120	17.58	-	7.17	10.41
7	C3	(NH_4_)_2_S_2_O_8_	120	30.40	-	14.92	15.47
8	C5	(NH_4_)_2_S_2_O_8_	120	41.67	1.89	23.10	16.67
9	C7	(NH_4_)_2_S_2_O_8_	120	46.35	5.87	24.99	15.54

^a^ Reaction conditions: iodobenzene (2.0 mmol), catalyst (50 mg), oxidant H_2_O_2_ (aqueous, 30 wt.%, 2.0 mmol) or oxidant (NH_4_)_2_S_2_O_8_ (aqueous solution, 37 wt.%, 2.0 mmol), and HCl (aqueous, 37 wt.%, 8.0 mmol pure HCl), 60 °C and 120 °C, reflux for 6 h. ^b^ Conversion of iodobenzene (%), determined by GC-MS (Figures S74–S82, Supplementary Materials), calculated by area normalization using the following formula: 1—[integrated area of residual iodobenzene]/([integrated area of residual iodobenzene] + [integrated area of all chlorinated products]) × 100%. ^c^ Yield of chlorinated product (%), determined by GC-MS (Figures S74–S82, Supplementary Materials), calculated by area normalization using the following formula: [integrated area of target chlorinated product]/([integrated area of residual iodobenzene] + [integrated area of all chlorinated products]) × 100%.

**Table 5:** Binding energies and atomic compositions on surfaces of crystalline by-products from iodobenzene chlorination (upper and lower layers; depth, 0–10 nm) ^a^.

Entry	S (2p)	O (1s)	C (1s)	N (1s)	Ba (3d)	I (3d)	Cl (2p)
C7-L1 ^b^	168.80 (10.65) ^d^	531.80 (32.45)	284.8 (52.16)	400.40 (66.56)	- ^e^	623.80 (0.45)	-
C7-L2 ^c^	168.80 (10.71)	531.80 (30.08)	284.8 (52.01)	401.80 (2.97)	779.80 (1.85)	-	202.80 (0.10)

^a^ XPS analysis of crystalline by-products obtained from the chlorination of iodobenzene. Reaction conditions: iodobenzene (2.0 mmol), C7 catalyst (50 mg), oxidant (NH_4_)_2_S_2_O_8_ (aqueous solution, 37 wt.%, 2.0 mmol), and HCl (aqueous, 37 wt.%, 8.0 mmol pure HCl), 120 °C, reflux for 6 h (Entry 9, Table 4). ^b^ Upper-layer crystals (C7-L1) from C7-facilitated reaction showing NH_4_HSO_4_ (Entry 9, Table 4). ^c^ Lower-layer crystals (C7-L2) from C7-facilitated reaction showing BaSO_4_ with minor NH_4_Cl (Entry 9, Table 4). ^d^ Binding energy (eV), along with atomic percentage (at%) in parentheses. ^e^ Not found or not counted by instrument due to low content.

## Data Availability

The original contributions presented in this study are included in the article/Supplementary Material. Further inquiries can be directed to the corresponding author.

## Supplementary Materials

The following supporting information can be downloaded at: https://www.mdpi.com/article/10.3390/nano16171120/s1. Table S1: The binding energy and atomic composition on the surface of the synthetic catalyst (depth 0–10 nm); Figure S1: XPS measurements of the O 1s regions of synthesized catalysts: (a) C1, (b) C2, (c) C3, (d) C4, (e) C5, (f) C6, (g) C7, (h) C8, and (i) C9; Figure S2: XPS measurements of the Ba 3d regions of synthesized catalysts: (a) C1, (b) C2, (c) C3, (d) C4, (e) C5, (f) C6, (g) C7, (h) C8, and (i) C9; Figure S3: XPS measurements of the S 2p regions of synthesized catalysts: (a) C1, (b) C2, (c) C3, (d) C4, (e) C5, (f) C6, (g) C7, (h) C8, and (i) C9; Figure S4: XPS measurements of the C 1s regions of synthesized catalysts: (a) C1, (b) C2, (c) C3, (d) C5, (e) C7, and (f) C9; Figure S5: XPS measurements of the Si 2p regions of synthesized catalysts: (a) C4, (b) C5, (c) C6, (d) C7, (e) C8, and (f) C; Figure S6: XPS measurements of the Al 2p regions of synthesized catalysts: (a) C6, (b) C7, and (c) C8; Figure S7: XPS measurements of the Na 1s regions of synthesized catalysts: (a) C4 and (b) C5; Figure S8: EDS mapping images of C1: (a) EDS layered image, (b) EDS spectrum, (c) O K*α*1, (d) S K*α*1, (e) C K*α*1,2, and (f) Ba M*α*; Figure S9: EDS mapping images of C2: (a) EDS layered image, (b) EDS Spectrum, (c) S K*α*1, (d) O K*α*1, (e) C K*α*1,2, and (f) Ba M*α*; Figure S10: EDS mapping images of C3: (a) EDS layered image, (b) EDS spectrum, (c) Ba M*α*, (d) S K*α*1, (e) O K*α*1, and (f) C K*α*1,2; Figure S11: EDS mapping images of C4: (a) EDS layered image, (b) EDS spectrum, (c) S K*α*1, (d) O K*α*1, (e) Si K*α*1, (f) Na K*α*1,2, and (g) Ba M*α*; Figure S12: EDS mapping images of C5: (a) EDS layered image, (b) EDS spectrum, (c) Si K*α*1, (d) O K*α*1, (e) S K*α*1, (f) Na K*α*1,2, (g) C K*α*1,2, and (h) Ba M*α*; Figure S13: EDS mapping images of C6: (a) EDS layered image, (b) EDS spectrum, (c) S K*α*1, (d) O K*α*1, (e) Si K*α*1, (f) Al K*α*1, (g) Na K*α*1,2, and (h) Ba M*α*; Figure S14: EDS mapping images of C8: (a) EDS layered image, (b) EDS spectrum, (c) S K*α*1, (d) O K*α*1, (e) Si K*α*1, (f) Al K*α*1, and (g) Ba M*α*; Figure S15: EDS mapping images of C9: (a) EDS layered image, (b) EDS spectrum, (c) S K*α*1, (d) C K*α*1,2, (e) O K*α*1, (f) Si K*α*1, (g) Al K*α*1, and (h) Ba M*α*; Table S2: Thermal analysis of synthesized catalysts derived from Figure 9; Figure S16: Proposed mechanism for the chlorination of toluene using H_2_O_2_ as an oxidant; Figure S17: GC part of GC-MS for Entry 1, Table 1; Figure S18: GC part of GC-MS for Entry 2, Table 1; Figure S19: GC part of GC-MS for Entry 3, Table 1; Figure S20: GC part of GC-MS for Entry 4, Table 1; Figure S21: GC part of GC-MS for Entry 5, Table 1; Figure S22: GC part of GC-MS for Entry 6, Table 1; Figure S23: GC part of GC-MS for Entry 7, Table 1; Figure S24: GC part of GC-MS for Entry 8, Table 1; Figure S25: GC part of GC-MS for Entry 9, Table 1; Figure S26: GC part of GC-MS for Entry 10, Table 1; Figure S27: GC part of GC-MS for Entry 11, Table 1; Figure S28: GC part of GC-MS for Entry 12, Table 1; Figure S29: GC part of GC-MS for Entry 13, Table 1; Figure S30: GC part of GC-MS for Entry 14, Table 1; Figure S31: GC part of GC-MS for Entry 15, Table 1; Figure S32: GC part of GC-MS for Entry 16, Table 1; Figure S33: GC part of GC-MS for Entry 17, Table 1; Figure S34: GC part of GC-MS for Entry 18, Table 1; Figure S35: GC part of GC-MS for Entry 19, Table 1; Figure S36: GC part of GC-MS for Entry 20, Table 1; Figure S37: GC part of GC-MS for Entry 21, Table 1; Figure S38: GC part of GC-MS for Entry 22, Table 1; Figure S39: GC part of GC-MS for Entry 23, Table 1; Figure S40: GC part of GC-MS for Entry 24, Table 1; Figure S41: GC part of GC-MS for Entry 25, Table 1; Figure S42: GC part of GC-MS for Entry 26, Table 1; Figure S43: GC part of GC-MS for Entry 27, Table 1; Figure S44: GC part of GC-MS for Entry 28, Table 1; Figure S45: Proposed mechanism for the chlorination of fluorobenzene using H_2_O_2_ and (NH_4_)_2_S_2_O_8_ as oxidants; Figure S46: GC part of GC-MS for Entry 1, Table 2; Figure S47: GC part of GC-MS for Entry 2, Table 2; Figure S48: GC part of GC-MS for Entry 3, Table 2; Figure S49: GC part of GC-MS for Entry 4, Table 2; Figure S50: GC part of GC-MS for Entry 5, Table 2; Figure S51: GC part of GC-MS for Entry 6, Table 2; Figure S52: GC part of GC-MS for Entry 7, Table 2; Figure S53: GC part of GC-MS for Entry 8, Table 2; Figure S54: GC part of GC-MS for Entry 9, Table 2; Figure S55: GC part of GC-MS for Entry 10, Table 2; Figure S56: Proposed mechanism for the chlorination of bromobenzene using H_2_O_2_ as an oxidant; Figure S57: GC part of GC-MS for Entry 1, Table 3; Figure S58: GC part of GC-MS for Entry 2, Table 3; Figure S59: GC part of GC-MS for Entry 3, Table 3; Figure S60: GC part of GC-MS for Entry 4, Table 3; Figure S61: GC part of GC-MS for Entry 5, Table 3; Figure S62: GC part of GC-MS for Entry 6, Table 3; Figure S63: GC part of GC-MS for Entry 7, Table 3; Figure S64: GC part of GC-MS for Entry 8, Table 3; Figure S65: GC part of GC-MS for Entry 9, Table 3; Figure S66: GC part of GC-MS for Entry 10, Table 3; Figure S67: GC part of GC-MS for Entry 11, Table 3; Figure S68: GC part of GC-MS for Entry 12, Table 3; Figure S69: GC part of GC-MS for Entry 13, Table 3; Figure S70: GC part of GC-MS for Entry 14, Table 3; Figure S71: GC part of GC-MS for Entry 15, Table 3; Figure S72: GC part of GC-MS for Entry 16, Table 3; Figure S73: Proposed mechanism for the chlorination of iodobenzene using H_2_O_2_ and (NH_4_)_2_S_2_O_8_ as oxidants; Figure S74: GC part of GC-MS for Entry 1, Table 4; Figure S75: GC part of GC-MS for Entry 2, Table 4; Figure S76: GC part of GC-MS for Entry 3, Table 4; Figure S77: GC part of GC-MS for Entry 4, Table 4; Figure S78: GC part of GC-MS for Entry 5, Table 4; Figure S79: GC part of GC-MS for Entry 6, Table 4; Figure S80: GC part of GC-MS for Entry 7, Table 4; Figure S81: GC part of GC-MS for Entry 8, Table 4; Figure S82: GC part of GC-MS for Entry 9, Table 4.
