# Supplementary material for: Selective Chlorination of Toluene and Halobenzenes Using Modified BaSO4-Supported Catalysts: A Sustainable Approach with (NH4)2S2O8 and H2O2 as Oxidants
Source: Nanomaterials (Basel). 2026 Sep 6;16(17):1120. doi: 10.3390/nano16171120 (PMC13567722; doi:10.3390/nano16171120)
Supplement: Supplementary file 1 [file nanomaterials-16-01120-s001.zip › nanomaterials-4549019-supplementary.pdf]

Supplementary Materials

Selective Chlorination of Toluene and Halobenzenes Using Modified $BaSO_4$-Supported Catalysts: A Sustainable Approach with $(NH_4)_2S_2O_8$ and H_2O_2 as Oxidants

Sidra Chaudhary [1], Sumaira Jamal [1], Mohsin Alam [1], Yuan Gao [2], Muhammad Faisal Altaf [2], Junsheng Bai [2], Yang Sun [1,3,*]

[1] Department of Applied Chemistry, School of Chemistry, Xi'an Jiaotong University, No. 28, Xianning West Road, Xi'an 710049, P.R. China

[2] Shaanxi Fuhua Chemical Co., Ltd., 2nd Industrial Road East, Zhuangli Industrial Zone, Fuping County, Weinan City, 711711, P.R. China

[3] Xi'an Biomass Green Catalysis and Advanced Valorization International Science and Technology Cooperation Base, No. 28, Xianning West Road, Xi'an 710049, P.R. China

Table of Contents

1. Binding Energies and Atomic Compositions on Surfaces of Synthesized Catalysts (Depth, 0–10 nm) (**Table S1**)
2. XPS Measurements of the O 1s Regions of Synthesized Catalysts (**Figure S1**)
3. XPS Measurements of the Ba 3d Regions of Synthesized Catalysts (**Figure S2**)
4. XPS Measurements of the S 2p Regions of Synthesized Catalysts (**Figure S3**)
5. XPS Measurements of the C 1s Regions of Synthesized Catalysts (**Figure S4**)

---

* Corresponding author. Tel.: +86 29 82663914 (Y. Sun); fax: +86 29 82668559 (Y. Sun).

E-mail address: sunyang79@mail.xjtu.edu.cn (Y. Sun).

6. XPS Measurements of the Si 2p Regions of Synthesized Catalysts (**Figure S5**)
7. XPS Measurements of the Al 2p Regions of Synthesized Catalysts (**Figure S6**)
8. XPS Measurements of the Na 1s Regions of Synthesized Catalysts (**Figure S7**)
9. EDS Mapping Images of C1 (**Figure S8**)
10. EDS Mapping Images of C2 (**Figure S9**)
11. EDS Mapping Images of C3 (**Figure S10**)
12. EDS Mapping Images of C4 (**Figure S11**)
13. EDS Mapping Images of C5 (**Figure S12**)
14. EDS Mapping Images of C6 (**Figure S13**)
15. EDS Mapping Images of C8 (**Figure S14**)
16. EDS Mapping Images of C9 (**Figure S15**)
17. Thermal Analysis of Synthesized Catalysts (**Table S2**)
18. Proposed Mechanism for the Chlorination of Toluene using H_2O_2 as an Oxidant (**Figure S16**)
19. GC-MS Examples for Table 1 (**Figures S17–S44**)
20. Proposed Mechanism for the Chlorination of fluorobenzene using H_2O_2 and $(NH_4)_2S_2O_8$ as Oxidants (**Figure S45**)
21. GC-MS examples for Table 2 (**Figures S46–S55**)
22. Proposed Mechanism for the Chlorination of Bromobenzene using H_2O_2 as an Oxidant (**Figure S56**)
23. GC-MS Examples for Table 3 (**Figures S57–S72**)
24. Proposed Mechanism for the Chlorination of Iodobenzene using H_2O_2 and $(NH_4)_2S_2O_8$ as Oxidants (**Figure S73**)
25. GC-MS Examples for Table 4 (**Figures S74–S82**)

1. Binding Energies and Atomic Compositions on Surfaces of Synthesized Catalysts (Depth, 0–10 nm)

Table S1. Binding energies and atomic compositions on surfaces of synthesized catalysts (depth, 0–10 nm).

Catalyst	Ba (3d)	S (2p)	O (1s)	C (1s)	Si (2p)	Al (2p) or Na (1s)
C1	779.80 (2.72) [a]	167.8 (10.61)	530.80 (30.74)	284.80 (55.92)	- [b]	-
C2	779.80 (2.39)	167.80 (10.31)	531.80 (29.95)	284.80 (57.34)	-	-
C3	779.80 (2.85)	168.80 (10.94)	531.80 (31.13)	284.80 (55.09)	-	-
C4	779.80 (2.68)	167.80 (12.32)	530.80 (34.26)	-	102.80 (50.18)	1071.80 (0.55) [c]
C5	779.80 (2.23)	167.80 (10.12)	530.80 (25.32)	284.80 (23.83)	102.80 (38.32)	1070.80 (0.19) [c]
C6	779.80 (4.60)	168.80 (17.88)	531.80 (52.52)	-	102.80 (22.74)	73.80 (2.27)
C7	779.80 (2.34)	167.80 (9.67)	531.80 (29.17)	283.80 (47.40)	101.80 (10.10)	73.80 (1.32)
C8	779.80 (3.20)	168.80 (15.11)	531.80 (54.31)	-	101.80 (27.04)	73.80 (0.34)
C9	779.80 (2.64)	168.80 (10.97)	531.80 (37.76)	284.80 (30.82)	102.80 (17.80)	-

[a] Binding energy (eV), along with atomic percentage (at%) in parentheses.

[b] Not found or not counted by instrument due to low content.

[c] Binding energy (atomic percentage, at%) of Na 1s photoelectron.

2. XPS Measurements of the O 1s Regions of Synthesized Catalysts

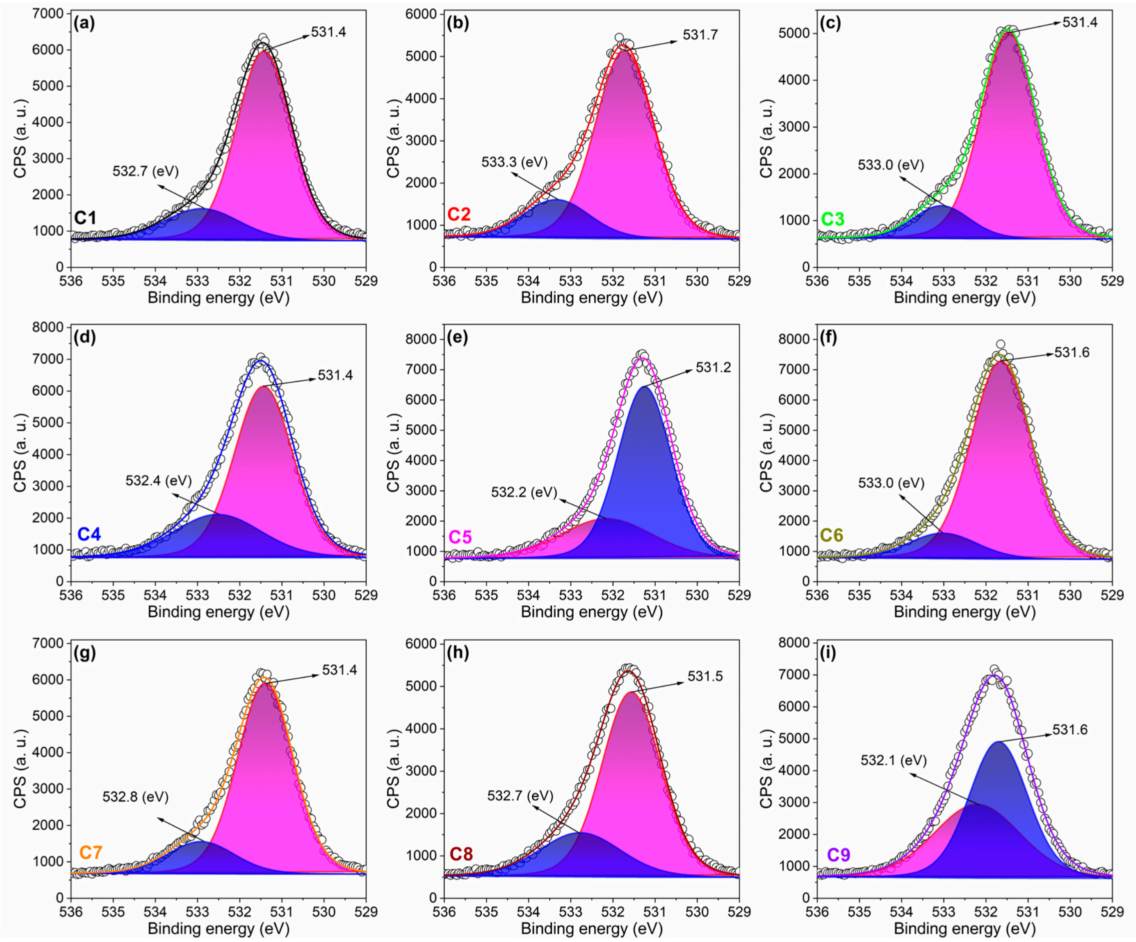

Figure S1. XPS measurements of the O 1s regions of synthesized catalysts: (a) C1; (b) C2; (c) C3; (d) C4; (e) C5; (f) C6; (g) C7; (h) C8; (i) C9.

3. XPS Measurements of the Ba 3d Regions of Synthesized Catalysts

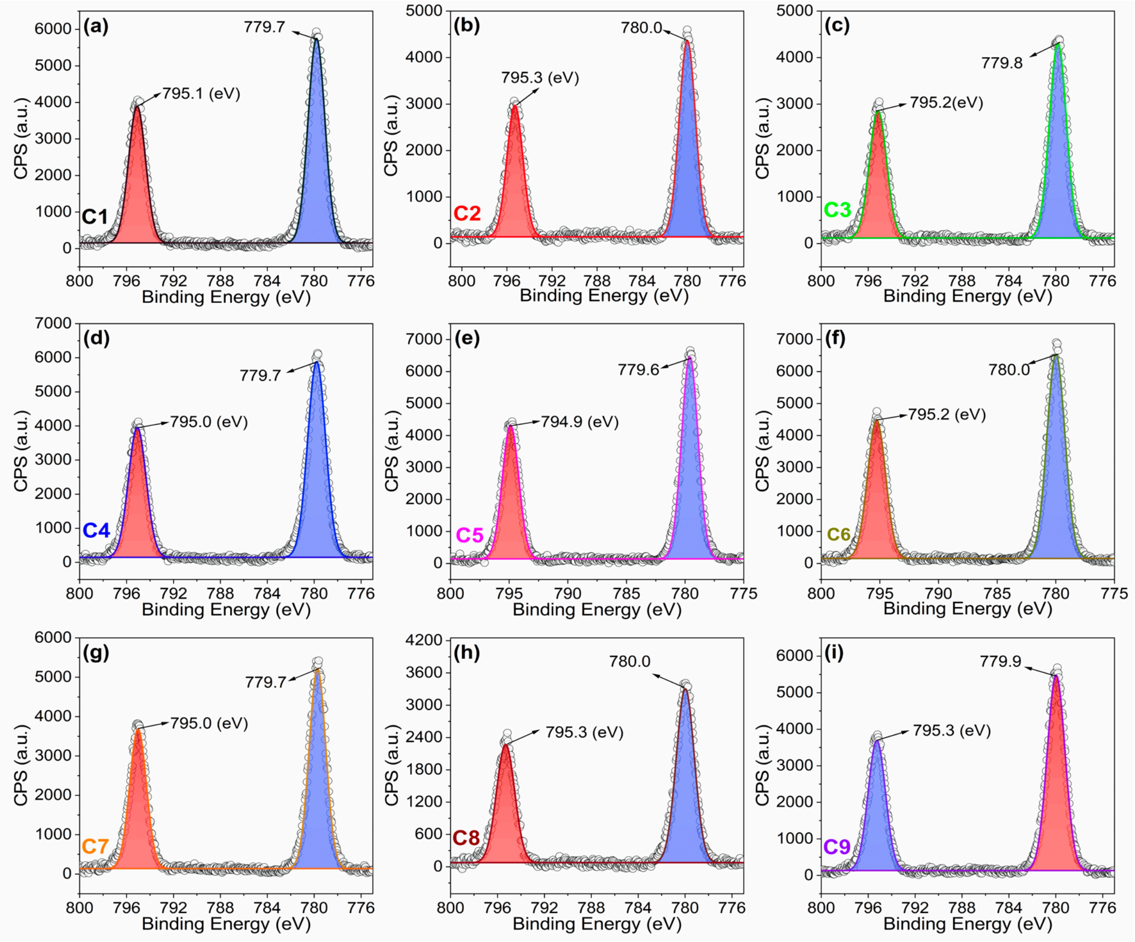

Figure S2. XPS measurements of the Ba 3d regions of synthesized catalysts: (a) C1; (b) C2; (c) C3; (d) C4; (e) C5; (f) C6; (g) C7; (h) C8; (i) C9.

4. XPS Measurements of the S 2p Regions of Synthesized Catalysts

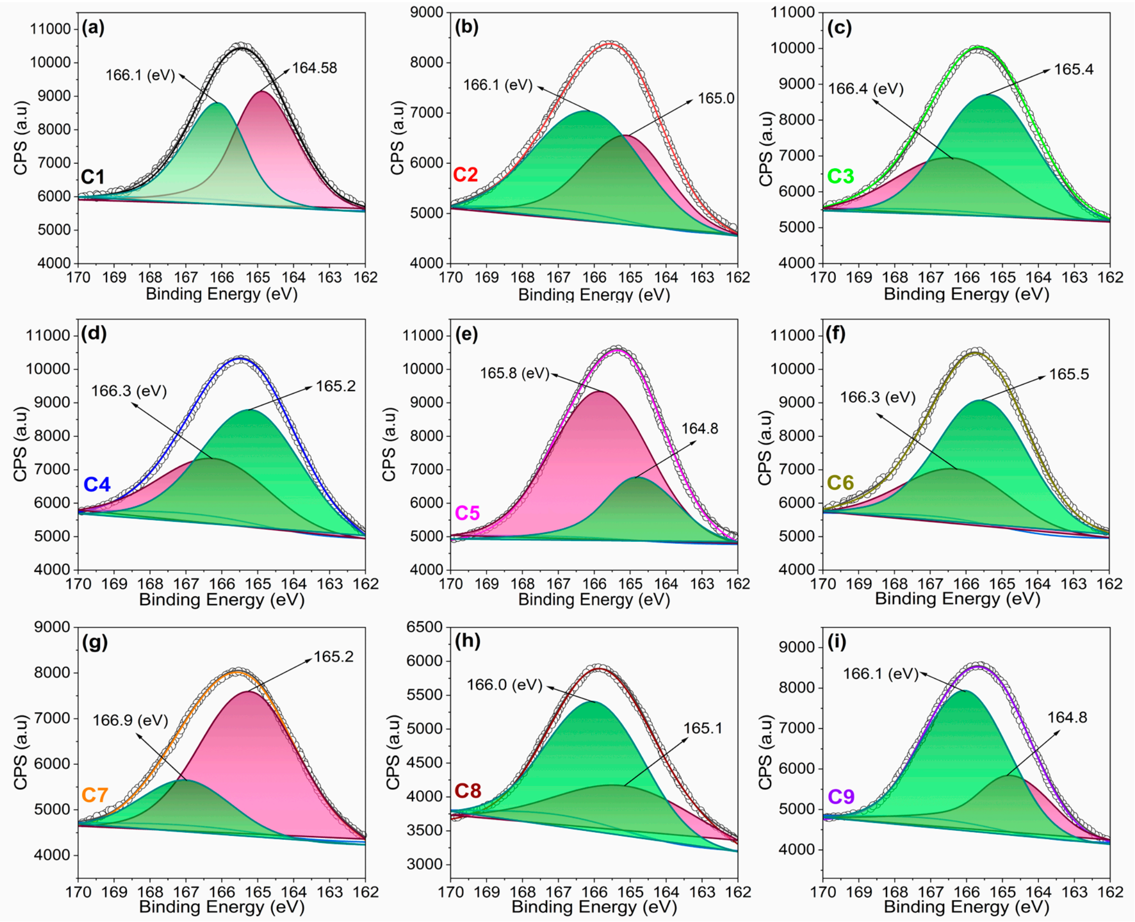

Figure S3. XPS measurements of the S 2p regions of synthesized catalysts: (a) C1; (b) C2; (c) C3; (d) C4; (e) C5; (f) C6; (g) C7; (h) C8; (i) C9.

5. XPS Measurements of the C 1s Regions of Synthesized Catalysts

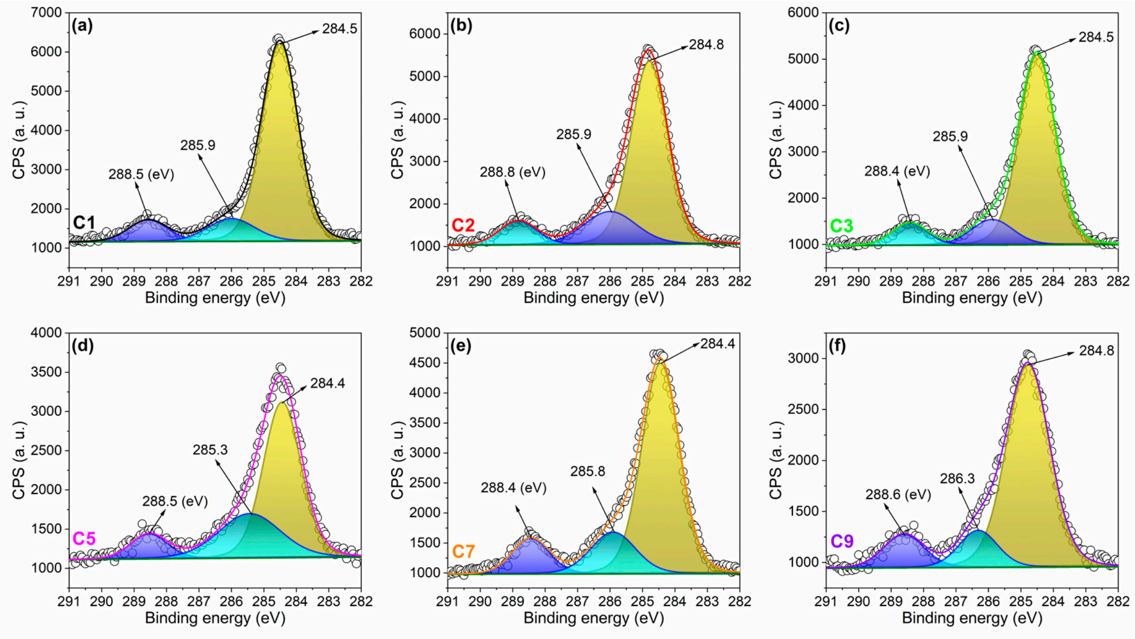

Figure S4. XPS measurements of the C 1s regions of synthesized catalysts: (a) C1; (b) C2; (c) C3; (d) C5; (e) C7; (f) C9.

6. XPS Measurements of the Si 2p Regions of Synthesized Catalysts

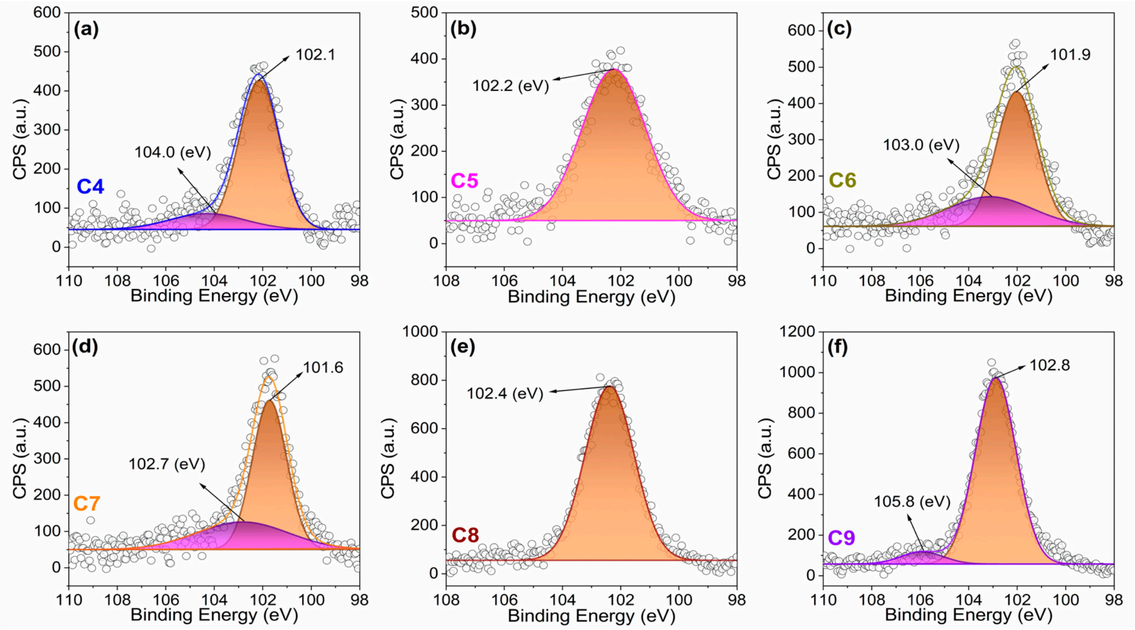

Figure S5. XPS measurements of the Si 2p regions of synthesized catalysts: (a) C4; (b) C5; (c) C6; (d) C7; (e) C8; (f) C.

7. XPS Measurements of the Al 2p Regions of Synthesized Catalysts

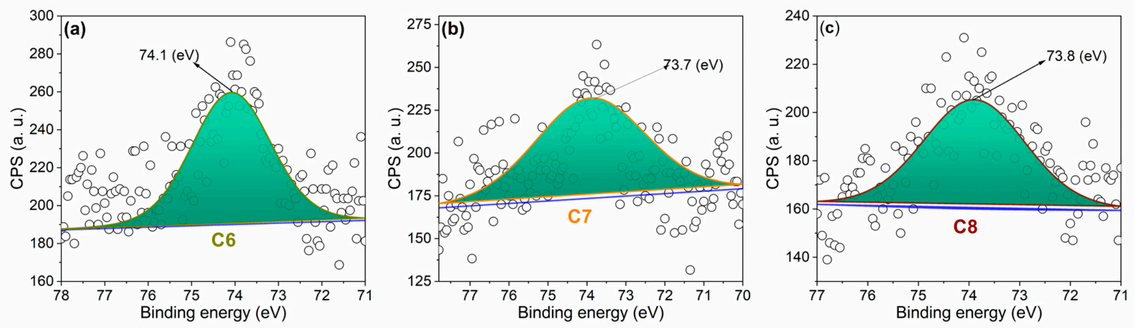

Figure S6. XPS measurements of the Al 2p regions of synthesized catalysts: (a) C6; (b) C7; (c) C8.

8. XPS Measurements of the Na 1s Regions of Synthesized Catalysts

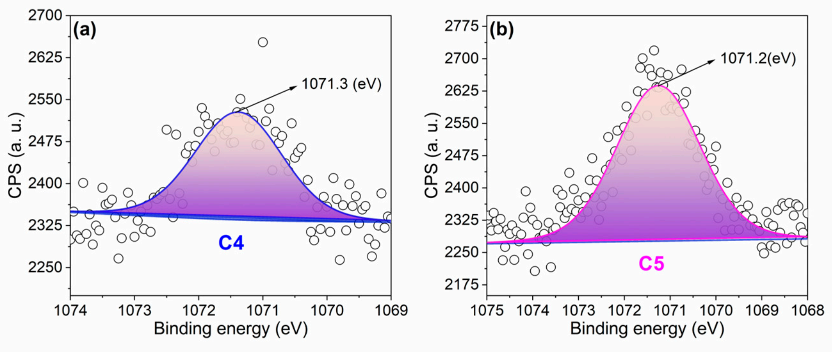

Figure S7. XPS measurements of the Na 1s regions of synthesized catalysts: (a) C4; (b) C5.

9. EDS Mapping Images of C1

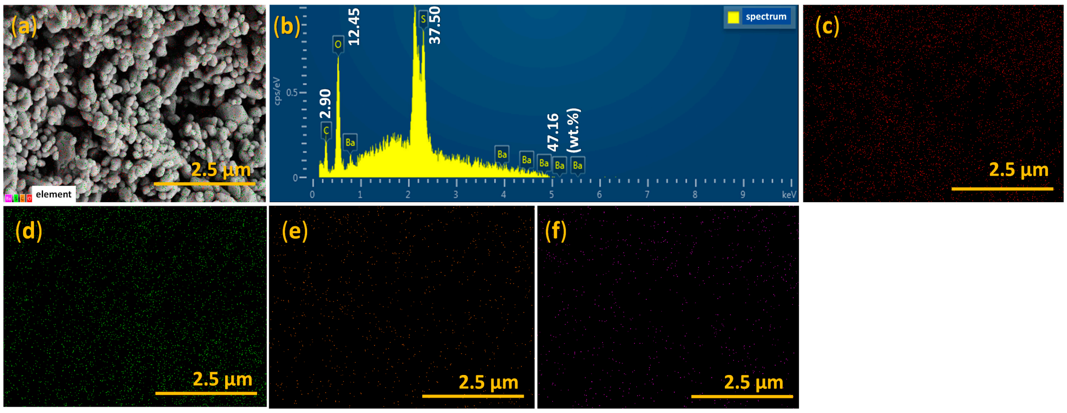

Figure S8. EDS mapping images of C1: (a) EDS layered image; (b) EDS Spectrum; (c) O Kα1； (d) S Kα1; (e) C Kα1,2; (f) Ba Mα.

10. EDS Mapping Images of C2

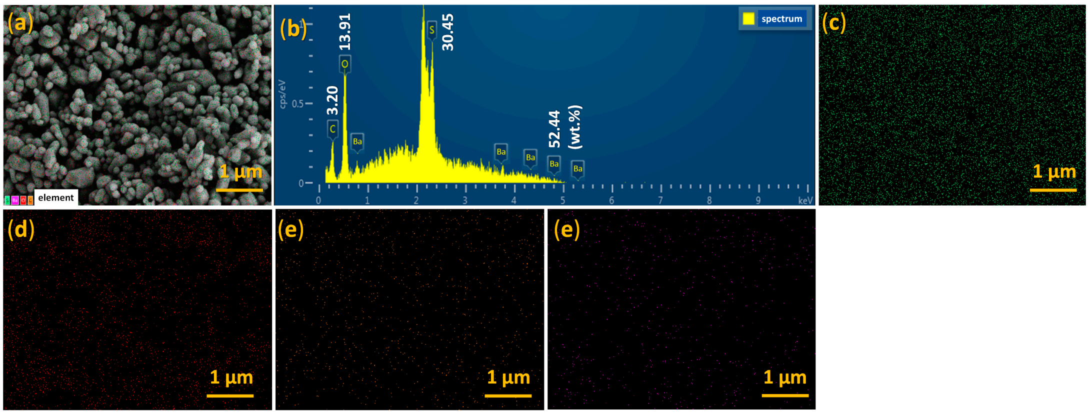

Figure S9. EDS mapping images of C2: (a) EDS layered image; (b) EDS Spectrum; (c) S Kα1； (d) O Kα1; (e) C Kα1,2; (f) Ba Mα.

11. EDS Mapping Images of C3

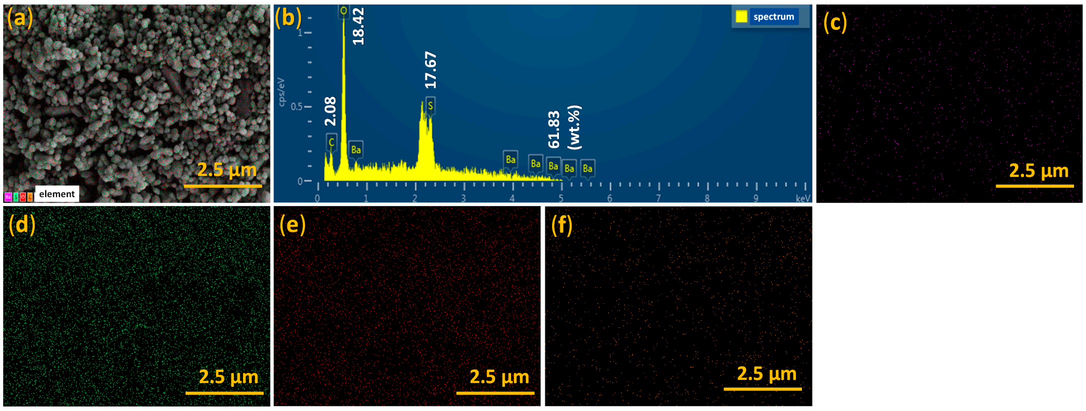

Figure S10. EDS mapping images of C3: (a) EDS layered image; (b) EDS Spectrum; (c) Ba Mα; (d) S Kα1; (e) O Kα1; (f) C Kα1,2.

12. EDS Mapping Images of C4

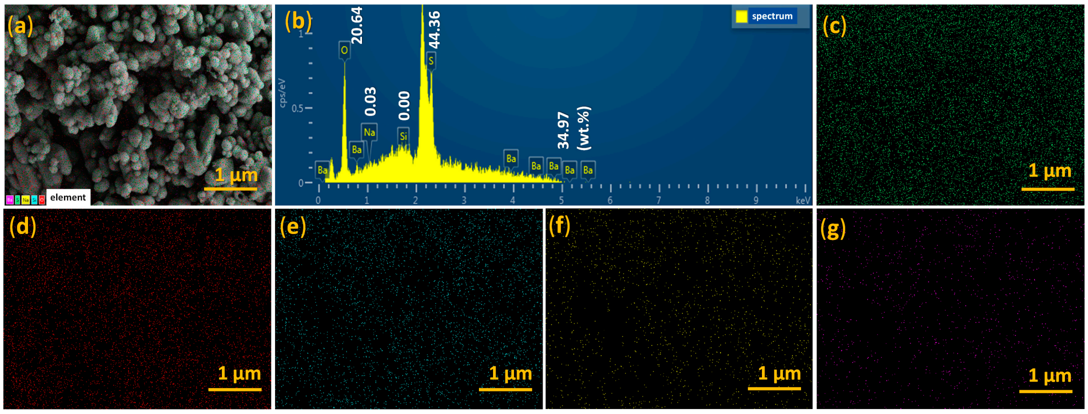

Figure S11. EDS mapping images of C4: (a) EDS layered image; (b) EDS Spectrum; (c) S Kα1; (d) O Kα1; (e) Si Kα1; (f) Na Kα1,2; (g) Ba Mα.

13. EDS Mapping Images of C5

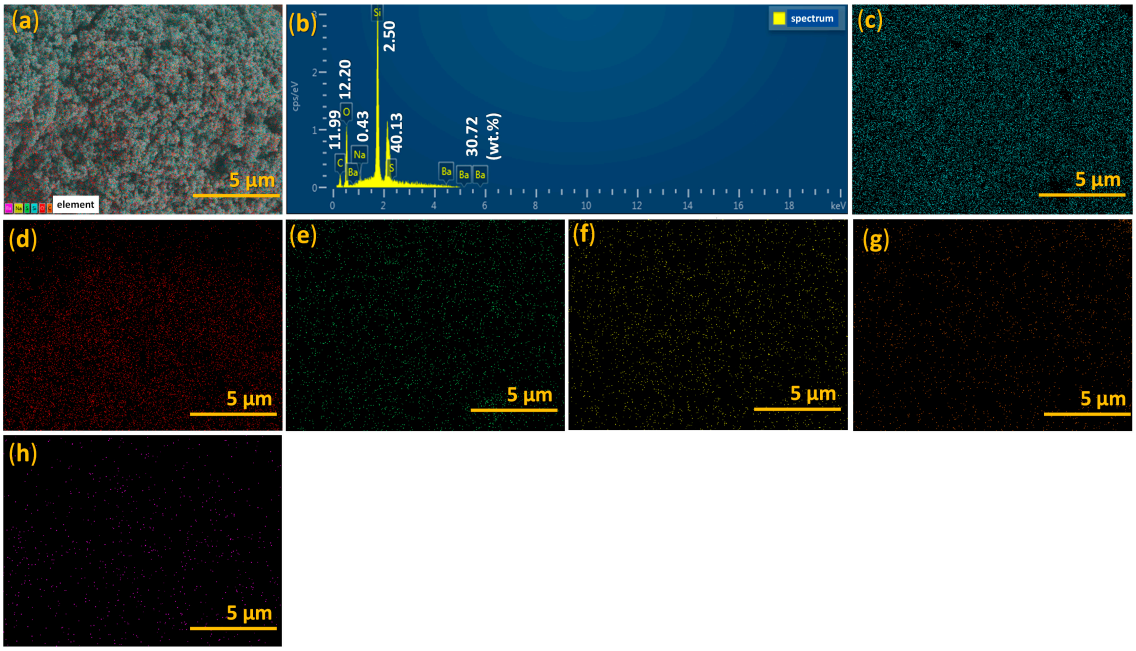

Figure S12. EDS mapping images of C5: (a) EDS layered image; (b) EDS Spectrum; (c) Si Kα1； (d) O Kα1; (e) S Kα1; (f) Na Kα1,2; (g) C Kα1,2; (h) Ba Mα.

14. EDS Mapping Images of C6

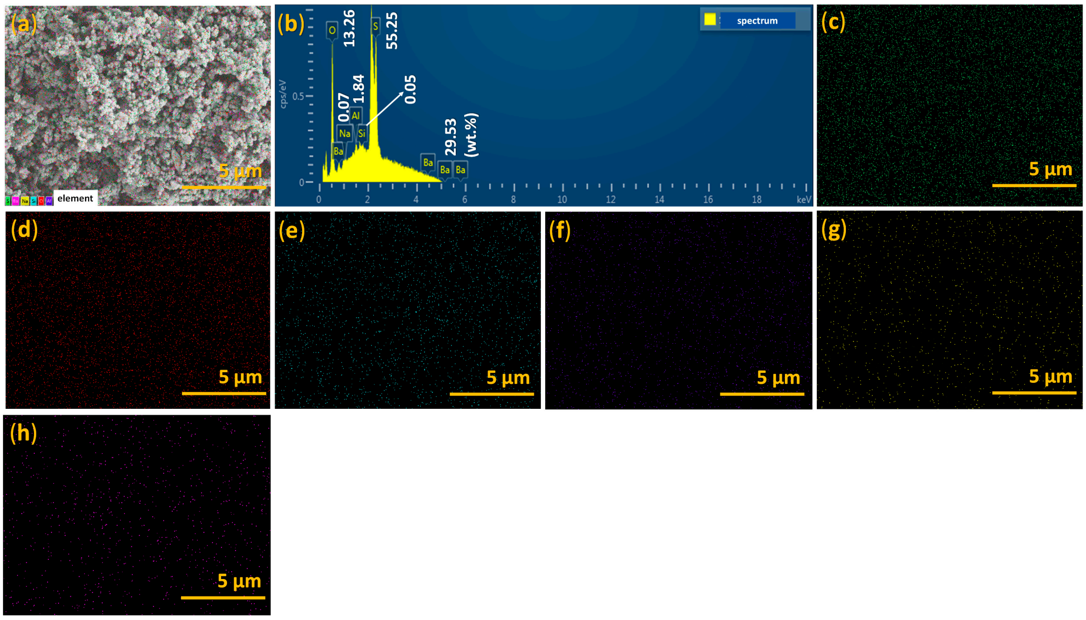

Figure S13. EDS mapping images of C6: (a) EDS layered image; (b) EDS Spectrum; (c) S Kα1； (d) O Kα1; (e) Si Kα1; (f) Al Kα1; (g) Na Kα1,2; (h) Ba Mα.

15. EDS Mapping Images of C8

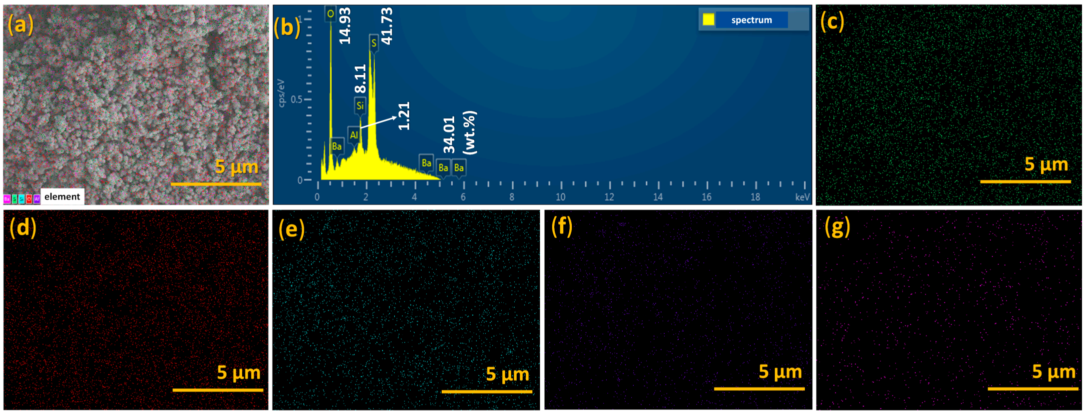

Figure S14. EDS mapping images of C8: (a) EDS layered image; (b) EDS Spectrum; (c) S Kα1； (d) O Kα1; (e) Si Kα1; (f) Al Kα1; (g) Ba Mα.

16. EDS Mapping Images of C9

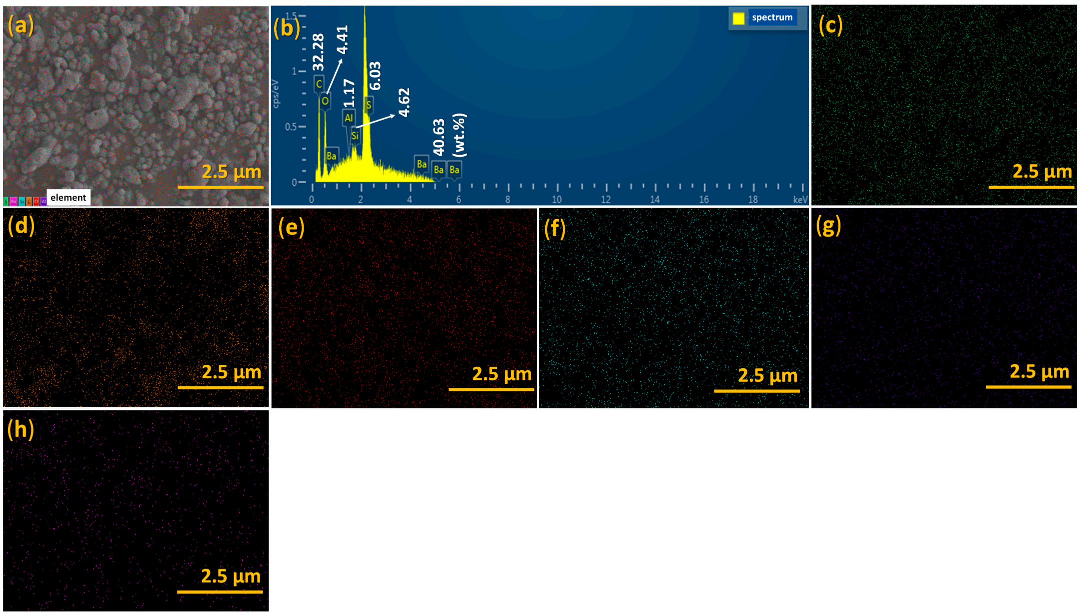

Figure S15. EDS mapping images of C9: (a) EDS layered image; (b) EDS Spectrum;

(c) S Kα1； (d) C Kα1,2; (e) O Kα1; (f) Si Kα1; (g) Al Kα1; (h) Ba Mα.

17. Thermal Analysis of Synthesized Catalysts

Table S2. Thermal analysis of synthesized catalysts derived from Figure 9.

Entry	Sample	Weight loss at 35–250 ^{o}C (%)	Weight loss at 250–800 ^{o}C (%)	Exothermic peak (^{o}C)
1	C1	3.48	1.29	33, 69, 251
2	C2	1.45	1.46	33, 69, 231
3	C3	21.50	16.87	33, 69, 256
4	C4	0.47	1.01	33
5	C5	0.20	0.22	34
6	C6	0.95	1.76	34, 272
7	C7	0.66	1.48	34, 270
8	C8	0.59	0.91	34
9	C9	0.57	0.44	34

18. Proposed Mechanism for the Chlorination of Toluene using H_2O_2 as an Oxidant

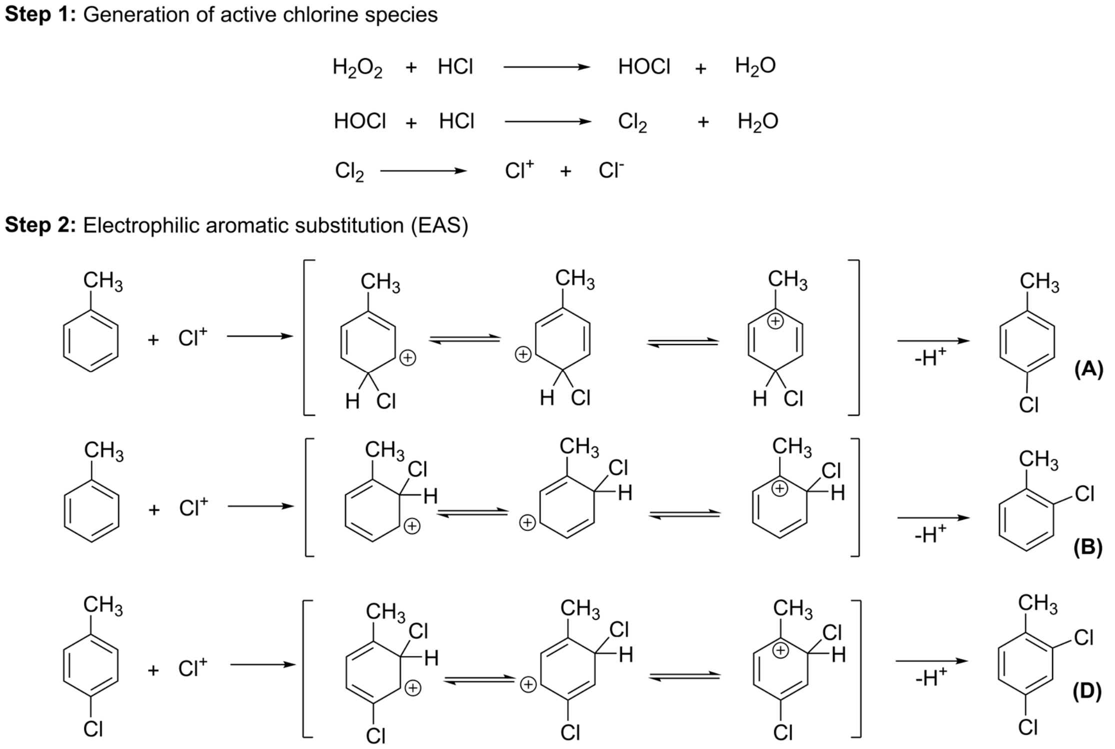

Figure S16. Proposed mechanism for the chlorination of toluene using H_2O_2 as an oxidant.

19. GC-MS Examples for Table 1

(1) Entry 1, Table 1

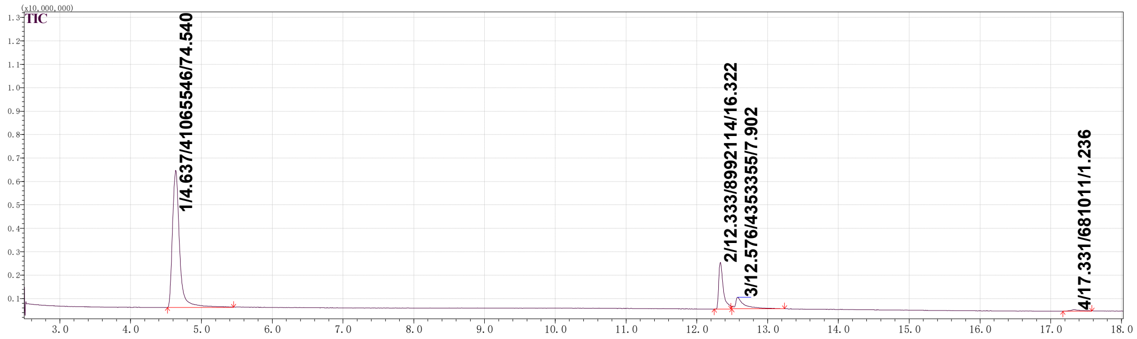

Figure S17. GC part of GC-MS for Entry 1, Table 1.

The peak 1 for t_R = 4.637 min with 74.540 % yield is:

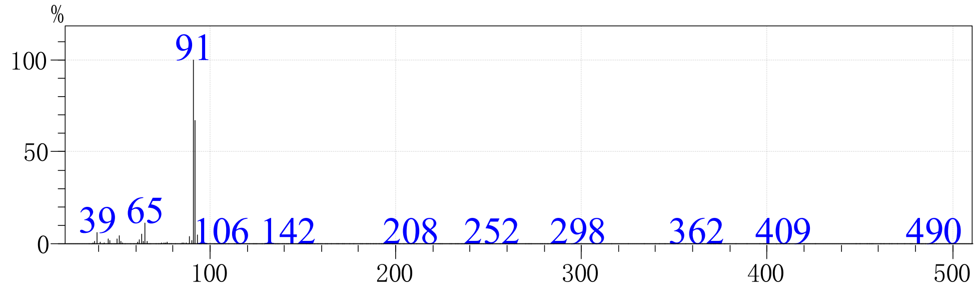

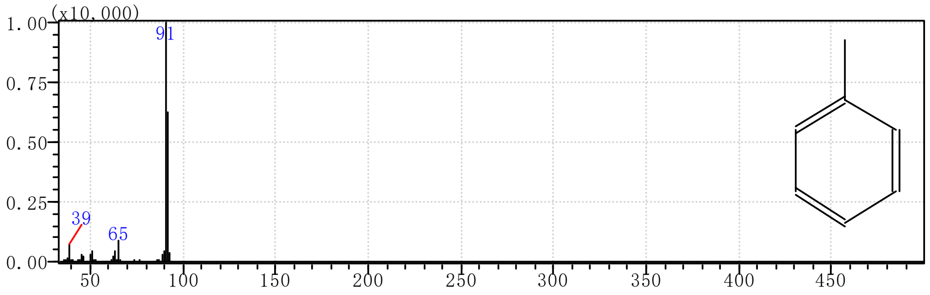

GC-MS: calcd. for C_7H_8 92, found 92 (C_7H_8). Table 1. Upper is the real mass spectrum, the lower is the standard mass spectrum in GC-MS database.

The peak 2 for t_R = 12.333 min with 16.322 % yield is:

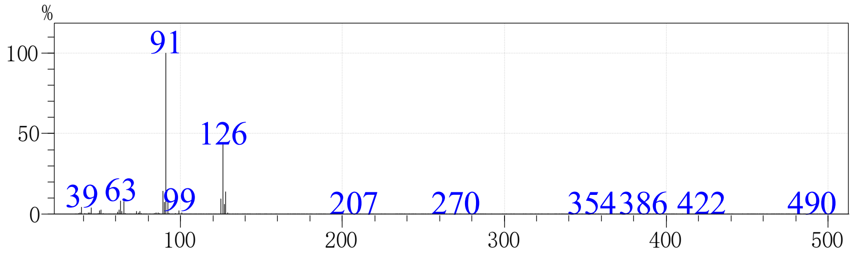

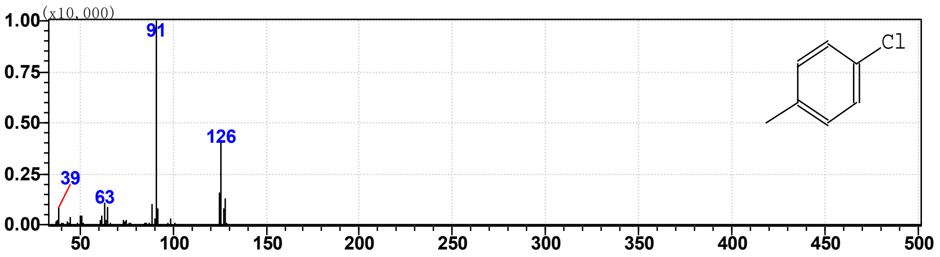

GC-MS: calcd. for C_7H_7Cl 126, found 126 (C_7H_7Cl). Table 1.

The peak 3 for t_R = 12.576 min with 7.902 % yield is:

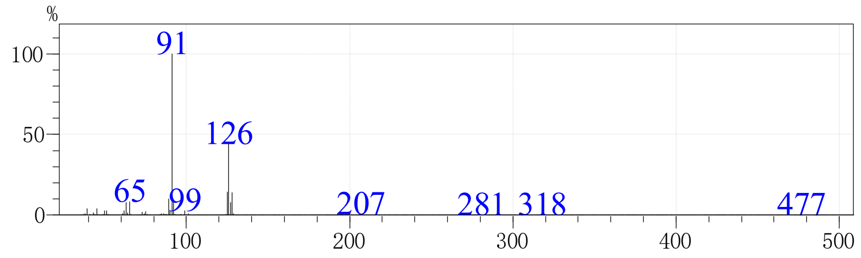

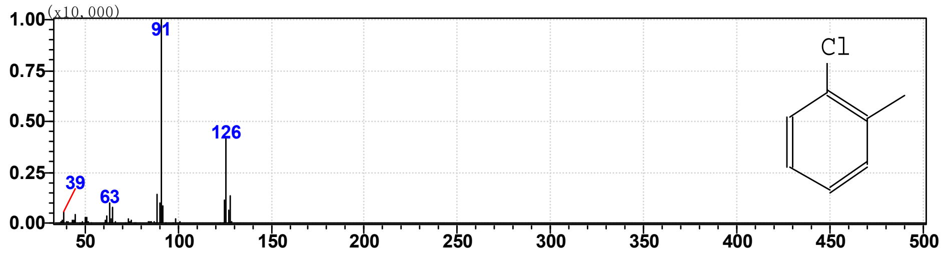

GC-MS: calcd. for C_7H_7Cl 126, found 126 (C_7H_7Cl). Table 1.

The peak 4 for t_R = 17.331 min with 1.236 % yield is:

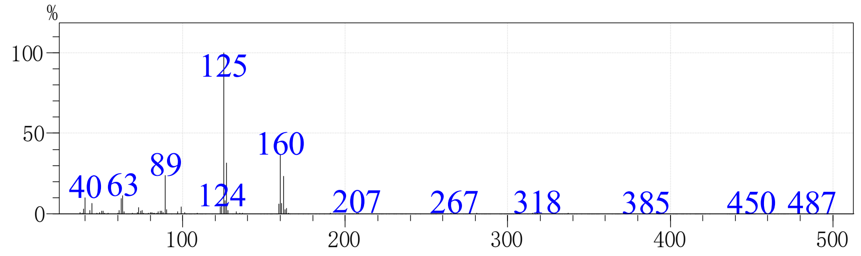

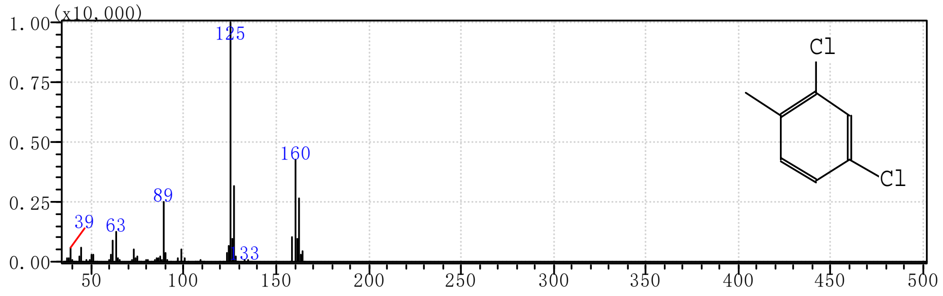

GC-MS: calcd. for $C_7H_6Cl_2$ 160, found 160 ($C_7H_6Cl_2$). Table 1.

The peak 1 for t_R = 4.637 min with 74.540 % yield is unreacted toluene. The peak 2 for t_R = 12.333 min with 16.322 % yield is 4-chlorotoluene. The peak 3 for t_R = 12.576 min with 7.902 % yield is 2-chlorotoluene. The peak 4 for t_R = 17.331 min with 1.236 % yield is 2,4-dichlorotoluene.

(2) Entry 2, Table 1

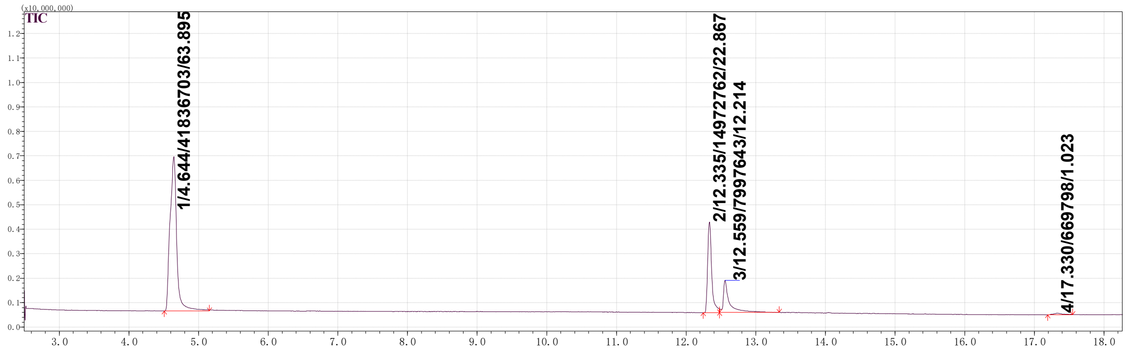

Figure S18. GC part of GC-MS for Entry 2, Table 1.

The peak 1 for t_R = 4.644 min with 63.895 % yield is unreacted toluene. The peak 2 for t_R = 12.355 min with 22.867 % yield is 4-chlorotoluene. The peak 3 for t_R = 12.559 min with 12.214 % yield is 2-chlorotoluene. The peak 4 for t_R = 17.330 min with 1.023 % yield is 2,4-dichlorotoluene.

(3) Entry 3, Table 1

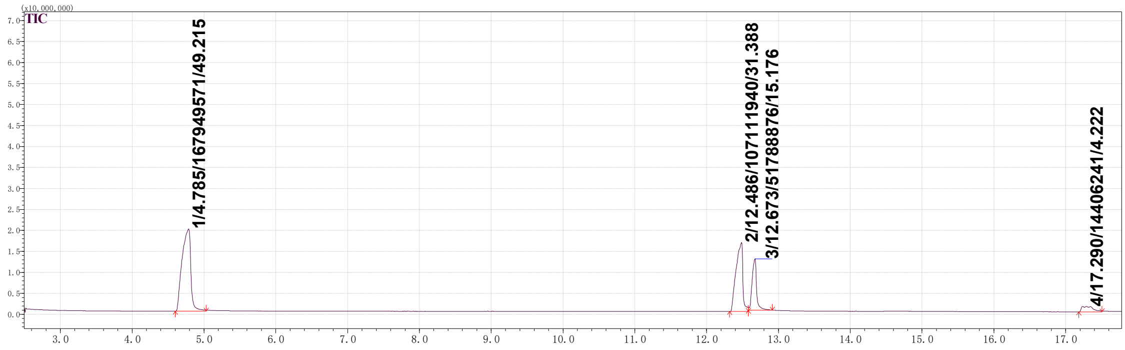

Figure 19. GC part of GC-MS for Entry 3, Table 1.

The peak 1 for t_R = 4.785 min with 49.215 % yield is unreacted toluene. The peak 2 for t_R = 12.486 min with 31.388 % yield is 4-chlorotoluene. The peak 3 for t_R = 12.673 min with 15.176 % yield is 2-chlorotoluene. The peak 4 for t_R = 17.290 min with 4.222 % yield is 2,4-dichlorotoluene.

(4) Entry 4, Table 1

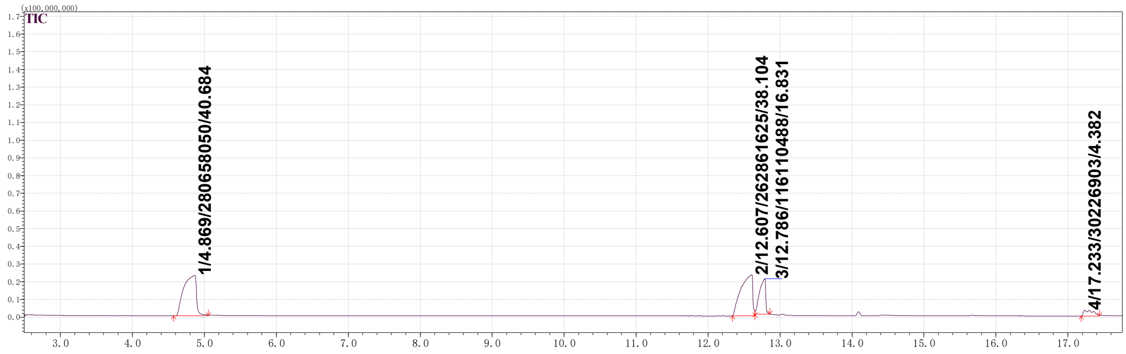

Figure S20. GC part of GC-MS for Entry 4, Table 1.

The peak 1 for t_R = 4.869 min with 40.684 % yield is unreacted toluene. The peak 2 for t_R = 12.607 min with 38.104 % yield is 4-chlorotoluene. The peak 3 for t_R = 12.786 min with 16.831 % yield is 2-chlorotoluene. The peak 4 for t_R = 17.233 min with 4.382 % yield is 2,4-dichlorotoluene.

(5) Entry 5, Table 1

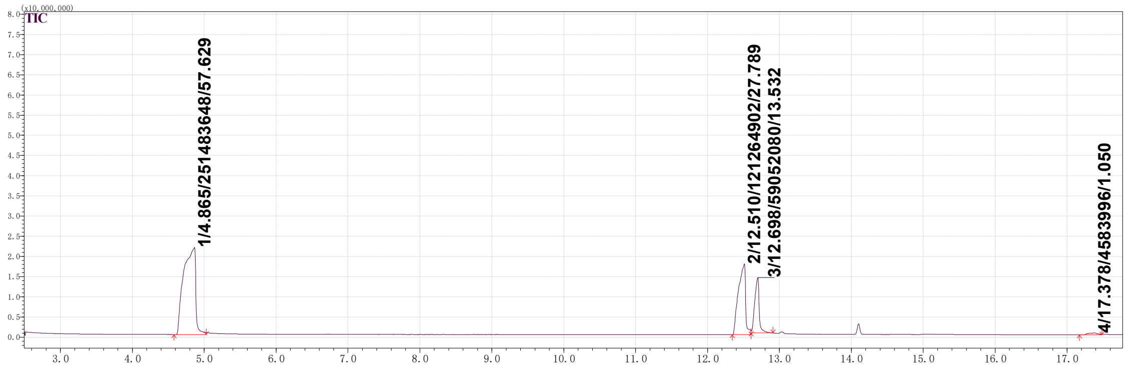

Figure S21. GC part of GC-MS for Entry 5, Table 1.

The peak 1 for t_R = 4.865 min with 57.629 % yield is unreacted toluene. The peak 2 for t_R = 12.510 min with 27.789 % yield is 4-chlorotoluene. The peak 3 for t_R = 12.698

min with 13.532 % yield is 2-chlorotoluene. The peak 4 for t_R = 17.378 min with 1.050 % yield is 2,4-dichlorotoluene.

(6) Entry 6, Table 1

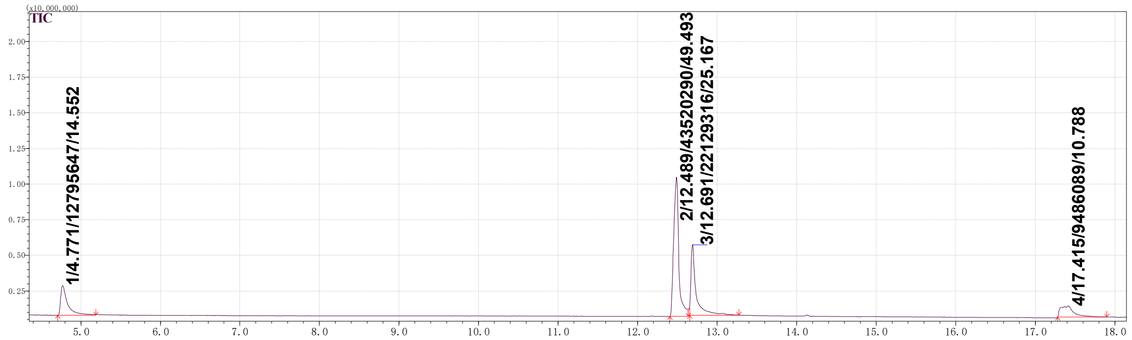

Figure S22. GC part of GC-MS for Entry 6, Table 1.

The peak 1 for t_R = 4.771 min with 14.552 % yield is unreacted toluene. The peak 2 for t_R = 12.489 min with 49.493 % yield is 4-chlorotoluene. The peak 3 for t_R = 12.691 min with 25.167 % yield is 2-chlorotoluene. The peak 4 for t_R = 17.415 min with 10.788 % yield is 2,4-dichlorotoluene.

(7) Entry 7, Table 1

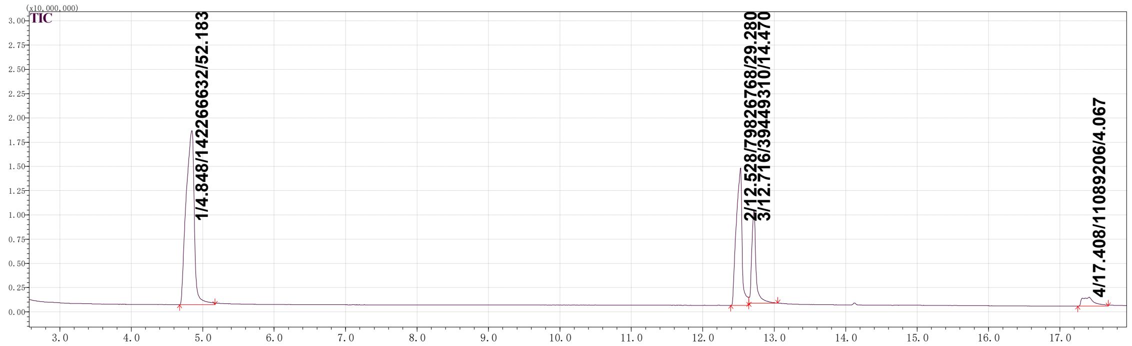

Figure S23. GC part of GC-MS for Entry 7, Table 1.

The peak 1 for t_R = 4.848 min with 52.183 % yield is unreacted toluene. The peak 2 for t_R = 12.528 min with 29.280 % yield is 4-chlorotoluene. The peak 3 for t_R = 12.716 min with 14.470 % yield is 2-chlorotoluene. The peak 4 for t_R = 17.408 min with 4.067 % yield is 2,4-dichlorotoluene.

(8) Entry 8, Table 1

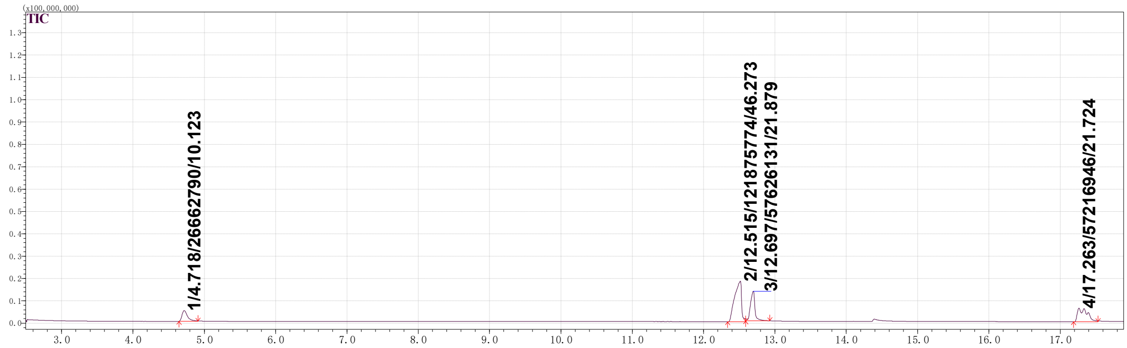

Figure S24. GC part of GC-MS for Entry 8, Table 1.

The peak 1 for t_R = 4.718 min with 10.123 % yield is is unreacted toluene. The peak 2 for t_R = 12.515 min with 46.273 % yield is 4-chlorotoluene. The peak 3 for t_R = 12.697 min with 21.879 % yield is 2-chlorotoluene. The peak 4 for t_R = 17.263 min with 21.724 % yield is 2,4-dichlorotoluene.

(9) Entry 9, Table 1

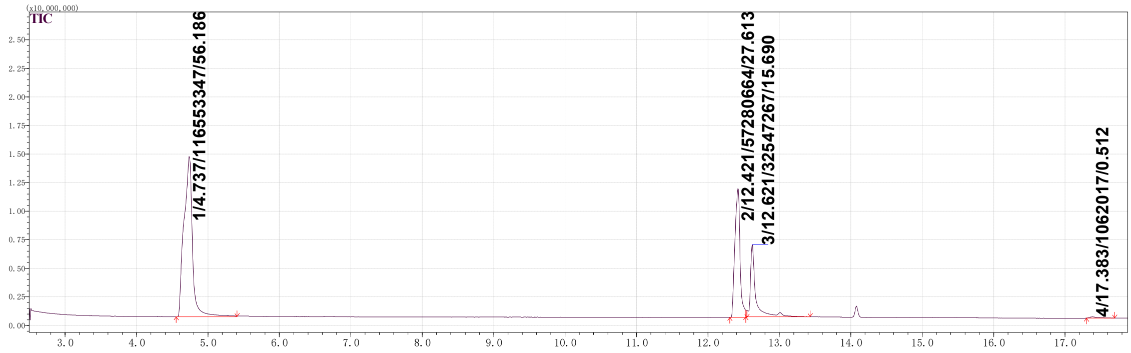

Figure S25. GC part of GC-MS for Entry 9, Table 1.

The peak 1 for t_R = 4.737 min with 56.186 % yield is unreacted toluene. The peak 2 for t_R = 12.421 min with 27.613 % yield is 4-chlorotoluene. The peak 3 for t_R = 12.621 min with 15.690 % yield is 2-chlorotoluene. The peak 4 for t_R = 17.383 min with 0.512% yield is 2,4-dichlorotoluene.

(10) Entry 10, Table 1

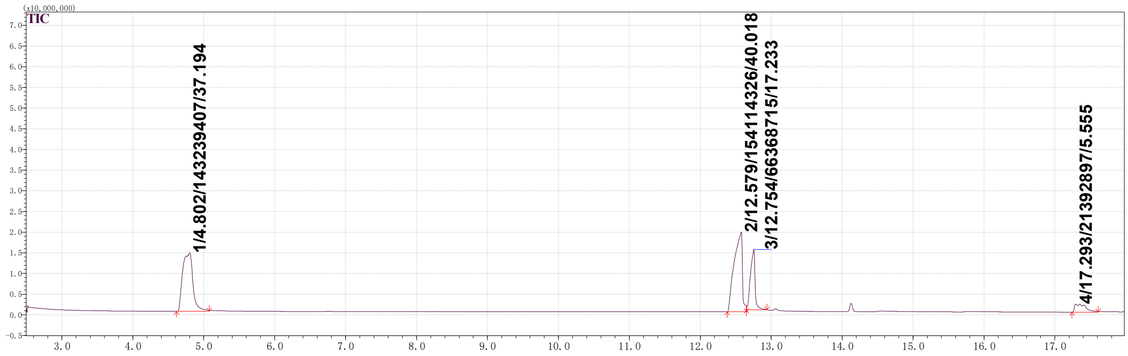

Figure S26. GC part of GC-MS for Entry 10, Table 1.

The peak 1 for t_R = 4.802 min with 37.194 % yield is unreacted toluene. The peak 2 for t_R = 12.579 min with 40.018 % yield is 4-chlorotoluene. The peak 3 for t_R = 12.754 min with 17.233 % yield is 2-chlorotoluene. The peak 4 for t_R = 17.293 min with 5.555 % yield is 2,4-dichlorotoluene.

(11) Entry 11, Table 1

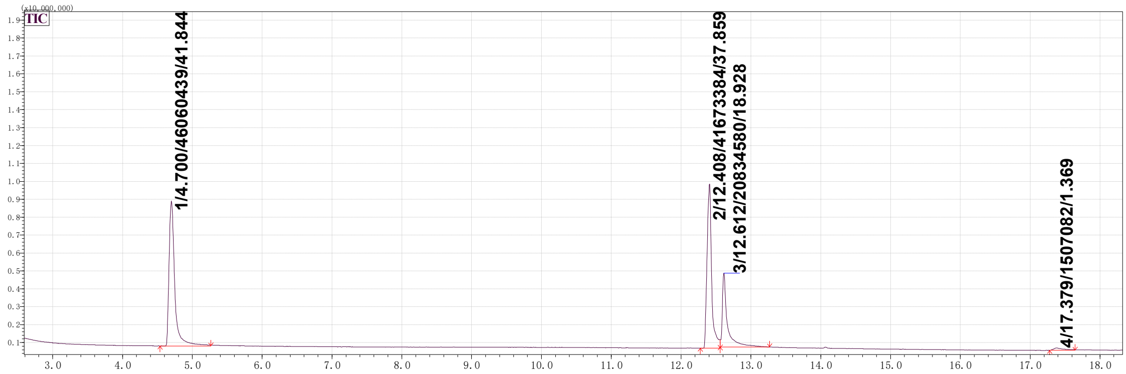

Figure S27. GC part of GC-MS for Entry 11, Table 1.

The peak 1 for t_R = 4.700 min with 41.844 % yield is unreacted toluene. The peak 2 for t_R = 12.408 min with 37.859 % yield is 4-chlorotoluene. The peak 3 for t_R = 12.612 min with 18.928 % yield is 2-chlorotoluene. The peak 4 for t_R = 17.379 min with 1.369% yield is 2,4-dichlorotoluene.

(12) Entry 12, Table 1

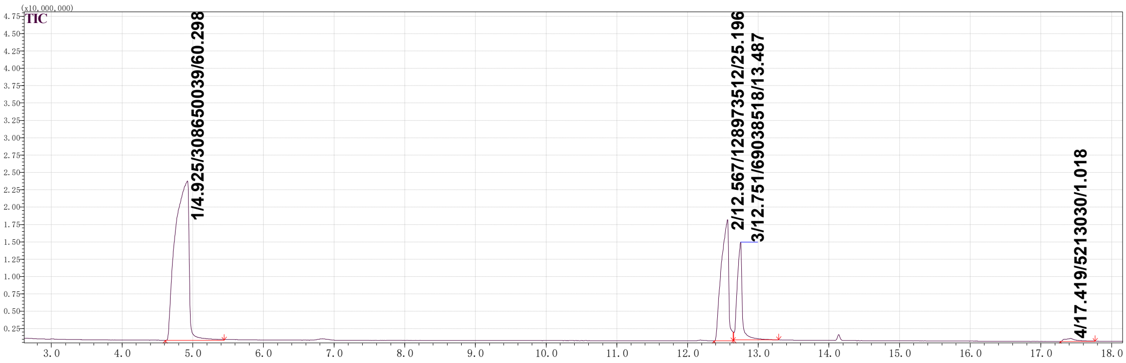

Figure S28. GC part of GC-MS for Entry 12, Table 1.

The peak 1 for t_R = 4.925 min with 60.298 % yield is unreacted toluene. The peak 2 for t_R = 12.567 min with 25.196 % yield is 4-chlorotoluene. The peak 3 for t_R = 12.751 min with 13.487 % yield is 2-chlorotoluene. The peak 4 for t_R = 17.419 min with 1.018 % yield is 2,4-dichlorotoluene.

(13) Entry 13, Table 1

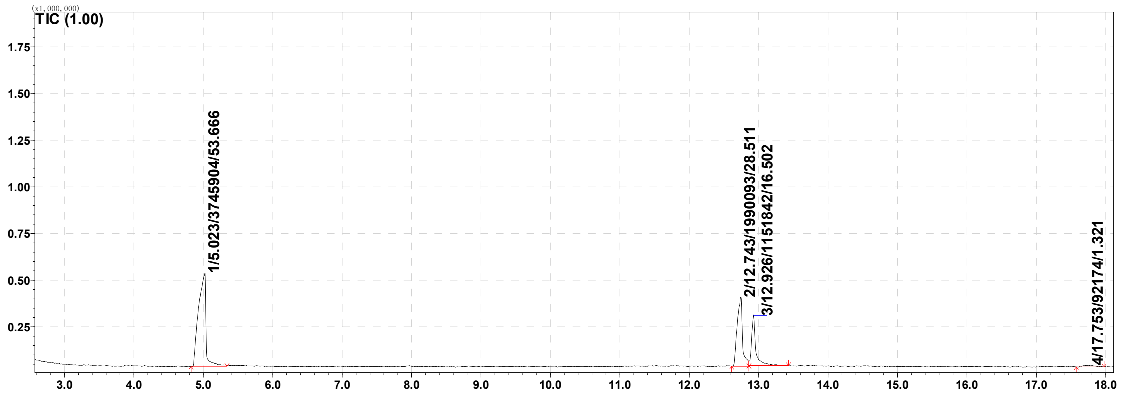

Figure S29. GC part of GC-MS for Entry 13, Table 1.

The peak 1 for t_R = 5.023 min with 53.666 % yield is unreacted toluene. The peak 2 for t_R = 12.743 min with 28.511 % yield is 4-chlorotoluene. The peak 3 for t_R = 12.926 min with 16.502 % yield is 2-chlorotoluene. The peak 4 for t_R = 17.753 min with 1.321 % yield is 2,4-dichlorotoluene.

(14) Entry 14, Table 1

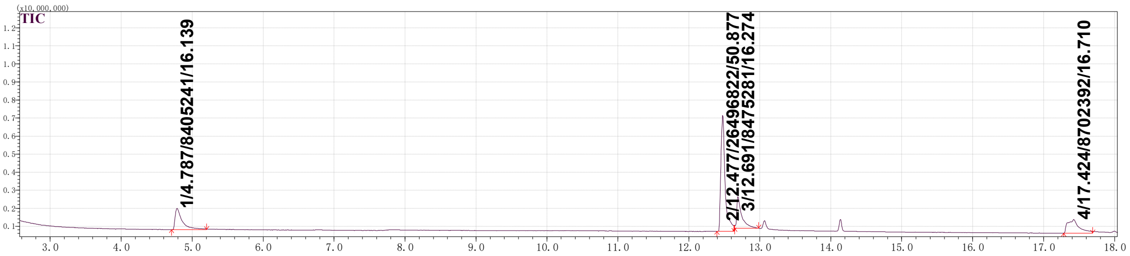

Figure S30. GC part of GC-MS for Entry 14, Table 1.

The peak 1 for t_R = 4.787 min with 16.139 % yield is unreacted toluene. The peak 2 for t_R = 12.477 min with 50.877 % yield is 4-chlorotoluene. The peak 3 for t_R = 12.691 min with 16.274 % yield is 2-chlorotoluene. The peak 4 for t_R = 17.242min with 16.710 yield is 2,4-dichlorotoluene.

(15) Entry 15, Table 1

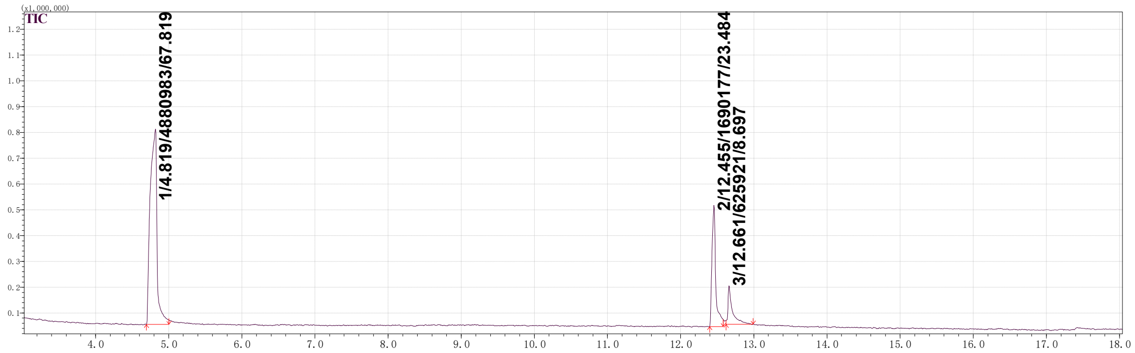

Figure S31. GC part of GC-MS for Entry 15, Table 1.

The peak 1 for t_R = 4.819 min with 67.819 % yield is unreacted toluene. The peak 2 for t_R = 12.455 min with 23.484 % yield is 4-chlorotoluene. The peak 3 for t_R = 12.661 min with 8.697 % yield is 2-chlorotoluene.

(16) Entry 16, Table 1

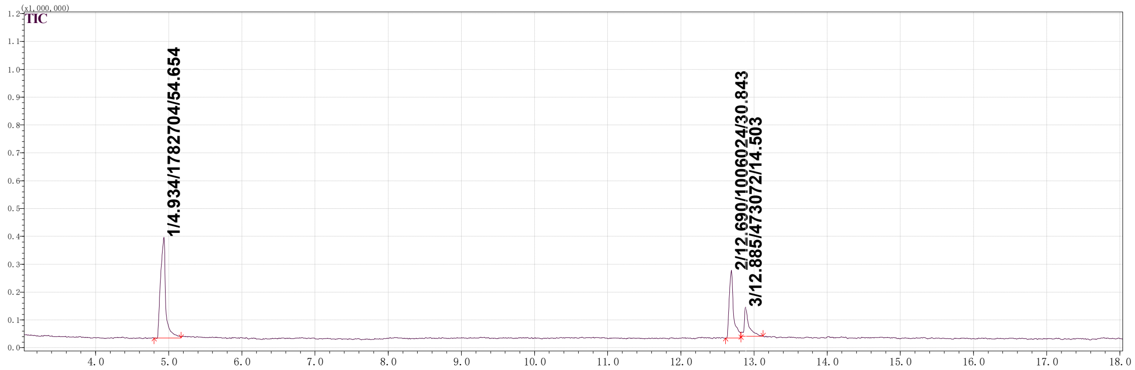

Figure S32. GC part of GC-MS for Entry 16, Table 1.

The peak 1 for t_R = 4.934 min with 54.654 % yield is unreacted toluene. The peak 2 for t_R = 12.690 min with 30.843 % yield is 4-chlorotoluene. The peak 3 for t_R = 12.885 min with 14.503 % yield is 2-chlorotoluene.

(17) Entry 17, Table 1

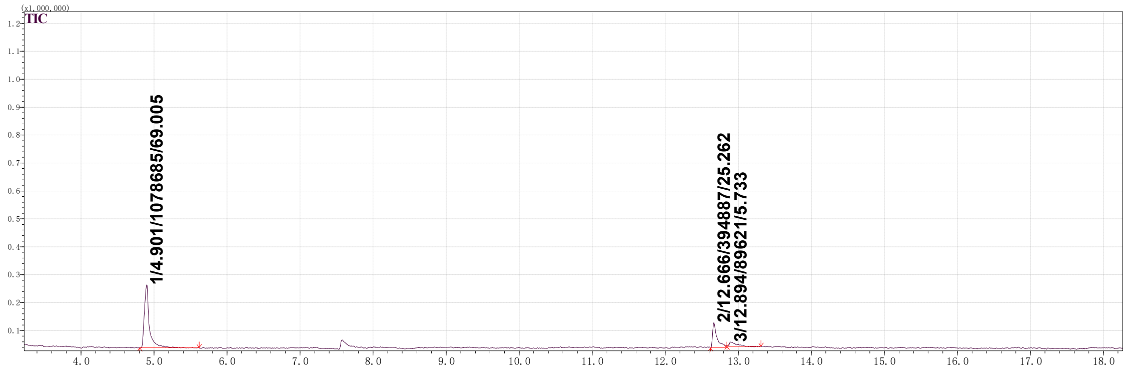

Figure S33. GC part of GC-MS for Entry 17, Table 1.

The peak 1 for t_R = 4.901min with 69.005 % yield is unreacted toluene. The peak 2 for t_R = 12.666 min with 25.262 % yield is 4-chlorotoluene. The peak 3 for t_R = 12.894 min with 5.733 % yield is 2-chlorotoluene.

(18) Entry 18, Table 1

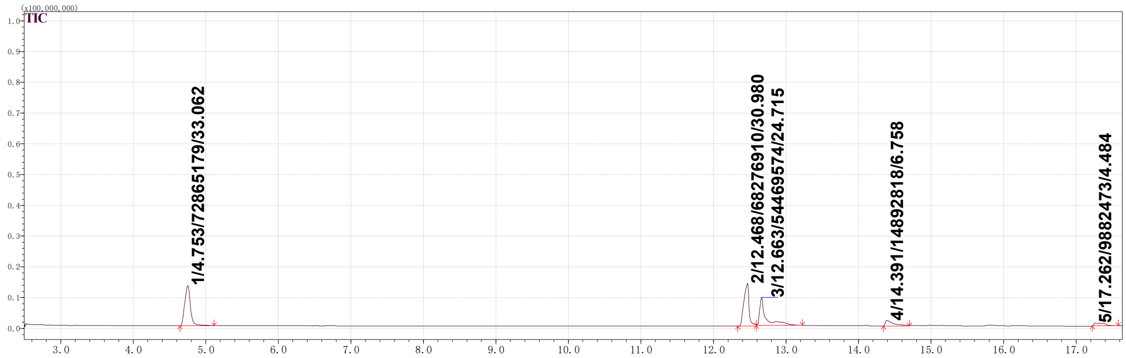

Figure S34. GC part of GC-MS for Entry 18, Table 1.

The peak 4 for t_R = 14.391 min with 6.758 % yield is:

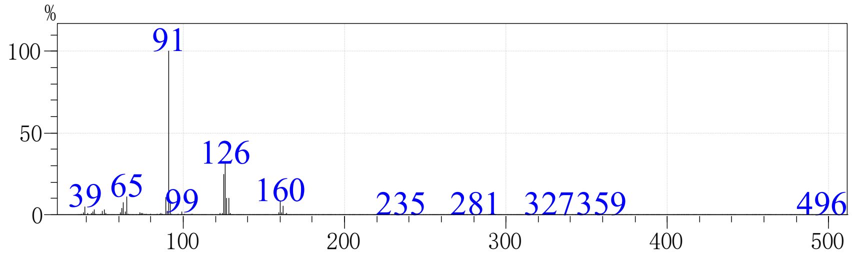

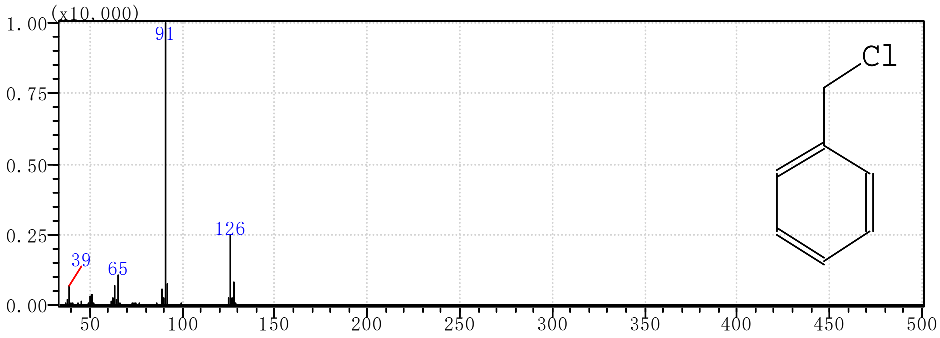

GC-MS: calcd. for C_7H_7Cl 126, found 126 (C_7H_7Cl). Table 1.

The peak 1 for t_R = 4.753 min with 33.062 % yield is unreacted toluene. The peak 2 for t_R = 12.468 min with 30.980 % yield is 4-chlorotoluene. The peak 3 for t_R = 12.663 min with 24.715 % yield is 2-chlorotoluene. The peak 4 for t_R = 14.391 min with 6.758 % yield is benzyl chloride. The peak 5 for t_R = 17.262 min with 4.484% yield is 2,4-dichlorotoluene.

(19) Entry 19, Table 1

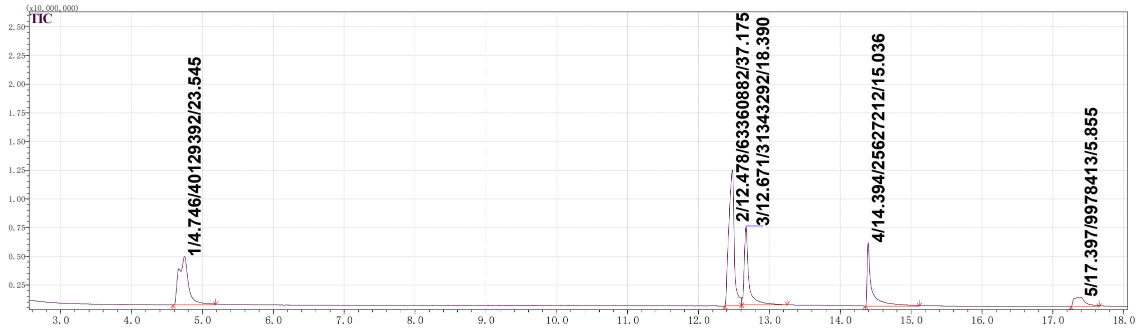

Figure S35. GC part of GC-MS for Entry 19, Table 1.

The peak 1 for t_R = 4.746 min with 23.545 % yield is unreacted toluene. The peak 2 for t_R = 12.478 min with 37.175 % yield is 4-chlorotoluene. The peak 3 for t_R = 12.671 min with 18.390 % yield is 2-chlorotoluene. The peak 4 for t_R = 14.394 min with 15.036 % yield is benzyl chloride. The peak 5 for t_R = 17.397 min with 5.855 % yield is 2,4-dichlorotoluene.

(20) Entry 20, Table 1

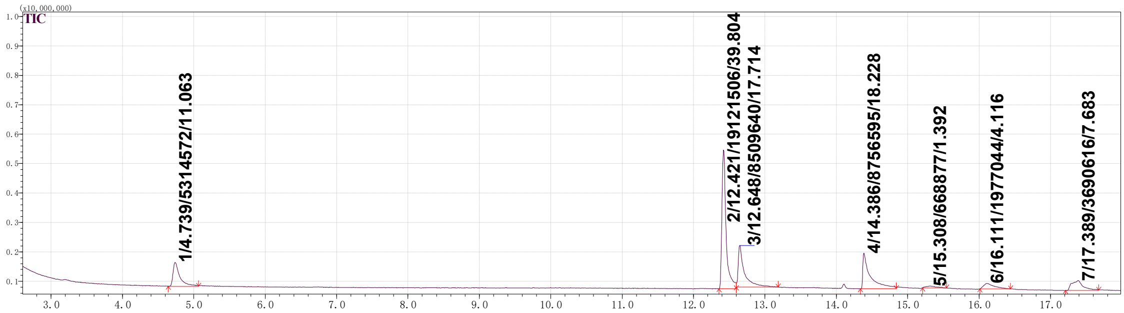

Figure S36. GC part of GC-MS for Entry 20, Table 1.

The peak 5 for t_R = 15.308 min with 1.392 % yield is:

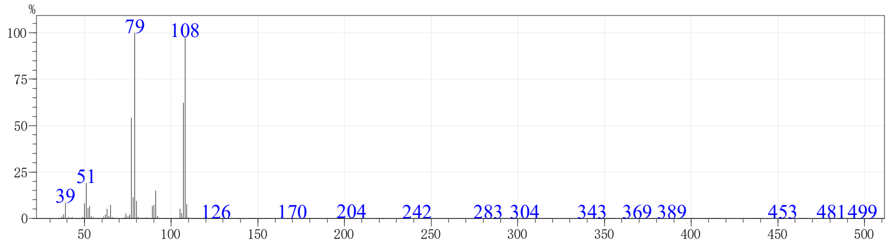

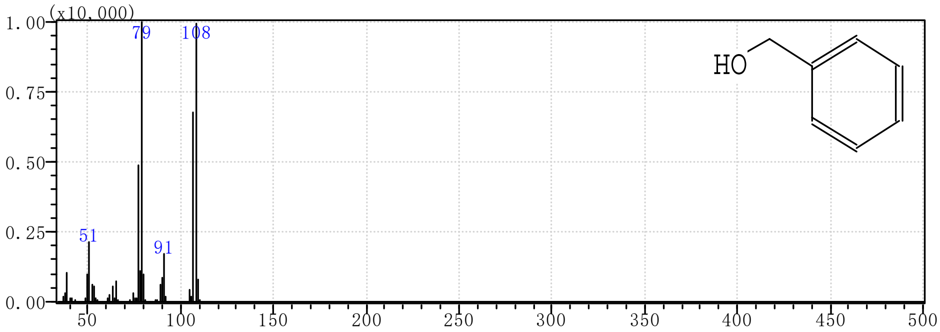

GC-MS: calcd. for C_7H_8O 108, found 108 (C_7H_8O). Table 1.

The peak 6 for t_R = 16.111 min with 4.116 % yield is:

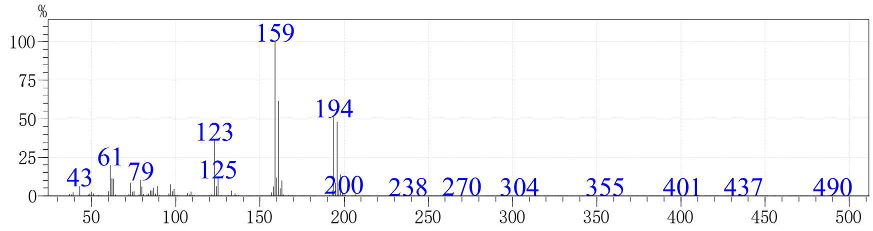

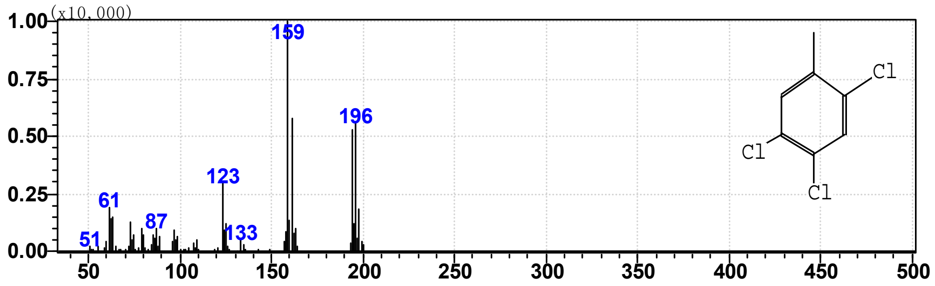

GC-MS: calcd. for $C_7H_5Cl_3$ 194, found 194 ($C_7H_5Cl_3$). Table 1.

The peak 1 for t_R = 4.739 min with 11.063 % yield is unreacted toluene. The peak 2 for t_R = 12.421 min with 39.804 % yield is 4-chlorotoluene. The peak 3 for t_R = 12.648 min with 17.714 % yield is 2-chlorotoluene. The peak 4 for t_R = 14.386 min with 18.228 % yield is benzyl chloride. The peak 5 for t_R = 15.308 min with 1.392 % yield is benzyl alcohol. The peak 6 for t_R = 16.111 min with 4.116 % yield is 2,4,5-trichlorotoluene. The peak 7 for t_R = 17.389 min with 7.683 % yield is 2,4-dichlorotoluene.

(21) Entry 21, Table 1

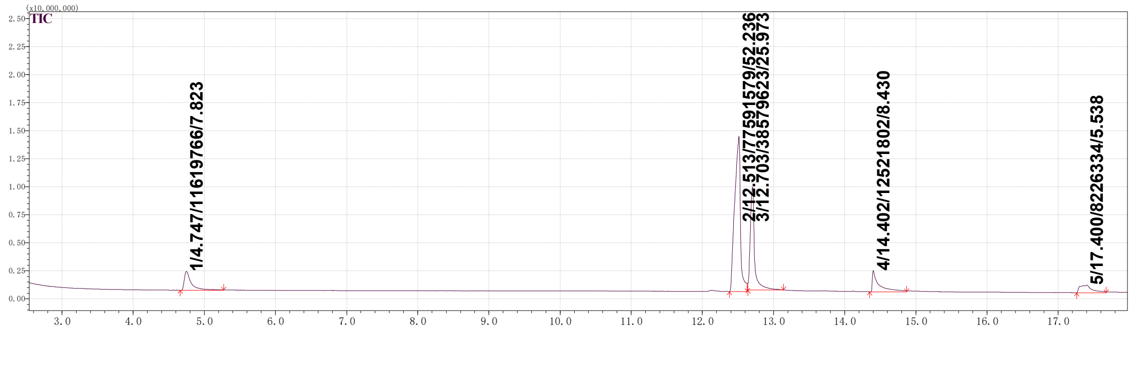

Figure S37. GC part of GC-MS for Entry 21, Table 1.

The peak 1 for t_R = 4.747 min with 7.823 % yield is unreacted toluene. The peak 2 for t_R = 12.513 min with 52.236 % yield is 4-chlorotoluene. The peak 3 for t_R = 12.703 min with 25.973 % yield is 2-chlorotoluene. The peak 4 for t_R = 14.402 min with 8.430 % yield is benzyl chloride. The peak 5 for t_R = 17.400 min with 5.538 % yield is 2,4-dichlorotoluene.

(22) Entry 22, Table 1

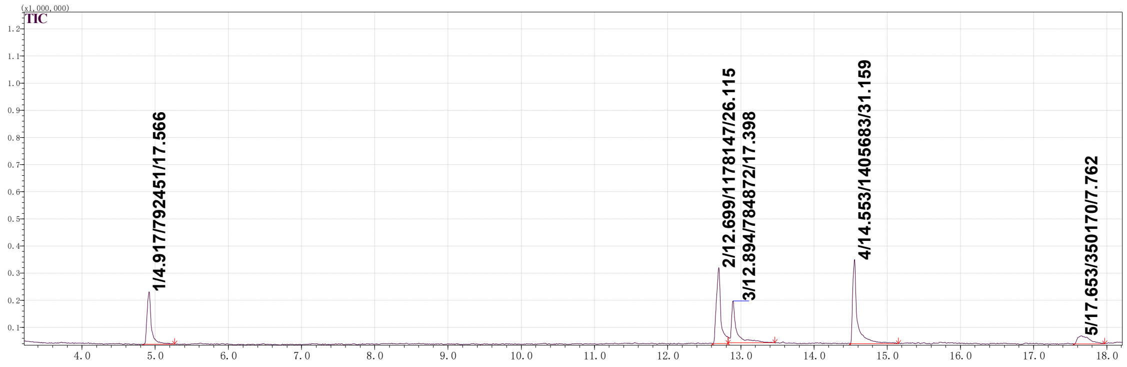

Figure S38. GC part of GC-MS for Entry 22, Table 1.

The peak 1 for t_R = 4.917 min with 17.566 % yield is unreacted toluene. The peak 2 for t_R = 12.699 min with 26.115 % yield is 4-chlorotoluene. The peak 3 for t_R = 12.894 min with 17.398 % yield is 2-chlorotoluene. The peak 4 for t_R = 14.553 min with 31.159 % yield is benzyl chloride. The peak 5 for t_R = 17.653 min with 7.762 % yield is 2,4-dichlorotoluene.

(23) Entry 23, Table 1

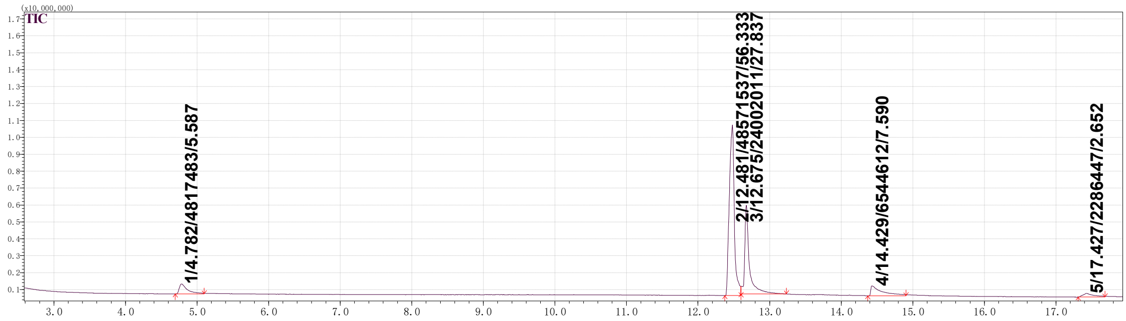

Figure S39. GC part of GC-MS for Entry 23, Table 1.

The peak 1 for t_R = 4.782 min with 5.587 % yield is unreacted toluene. The peak 2 for t_R = 12.481 min with 56.333 % yield is 4-chlorotoluene. The peak 3 for t_R = 12.675 min with 27.837 % yield is 2-chlorotoluene. The peak 4 for t_R = 14.429 min with 7.590 % yield is benzyl chloride. The peak 5 for t_R = 17.427 min with 2.652 % yield is 2,4-dichlorotoluene.

(24) Entry 24, Table 1

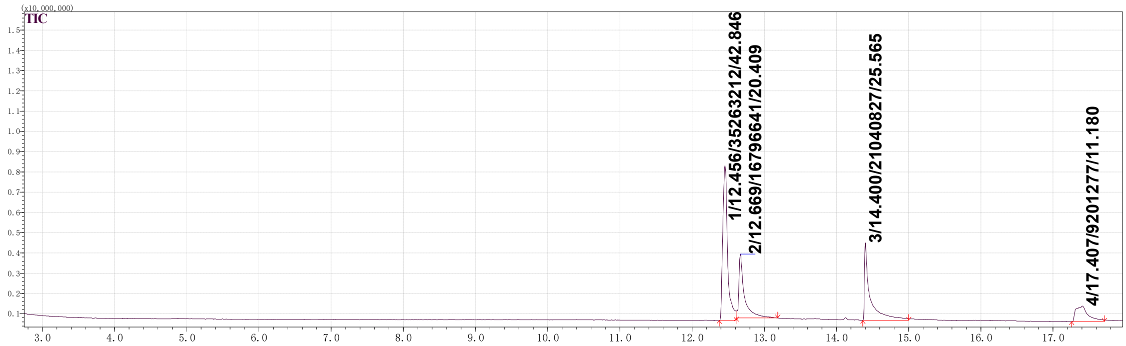

Figure S40. GC part of GC-MS for Entry 24, Table 1.

The peak 1 for t_R = 12.456 min with 42.846 % yield is 4-chlorotoluene. The peak 2 for t_R = 12.669 min with 20.409 % yield is 2-chlorotoluene. The peak 3 for t_R = 14.400 min with 25.565 % yield is benzyl chloride. The peak 4 for t_R = 17.407 min with 11.180 % yield is 2,4-dichlorotoluene.

(25) Entry 25, Table 1

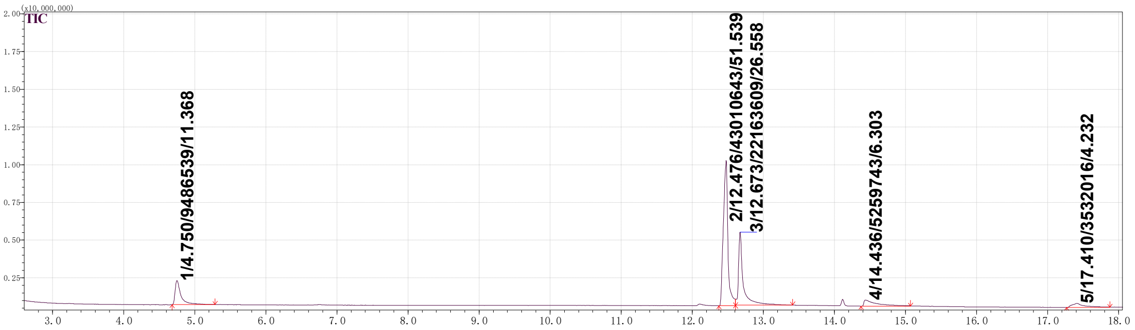

Figure S41. GC part of GC-MS for Entry 25, Table 1.

The peak 1 for t_R = 4.750 min with 11.368 % yield is unreacted toluene. The peak 2 for t_R = 12.476 min with 51.539 % yield is 4-chlorotoluene. The peak 3 for t_R = 12.673 min with 26.558 % yield is 2-chlorotoluene. The peak 4 for t_R = 14.436 min with 6.303 % yield is benzyl chloride. The peak 5 for t_R = 17.410 min with 4.232 % yield is 2,4-dichlorotoluene.

(26) Entry 26, Table 1

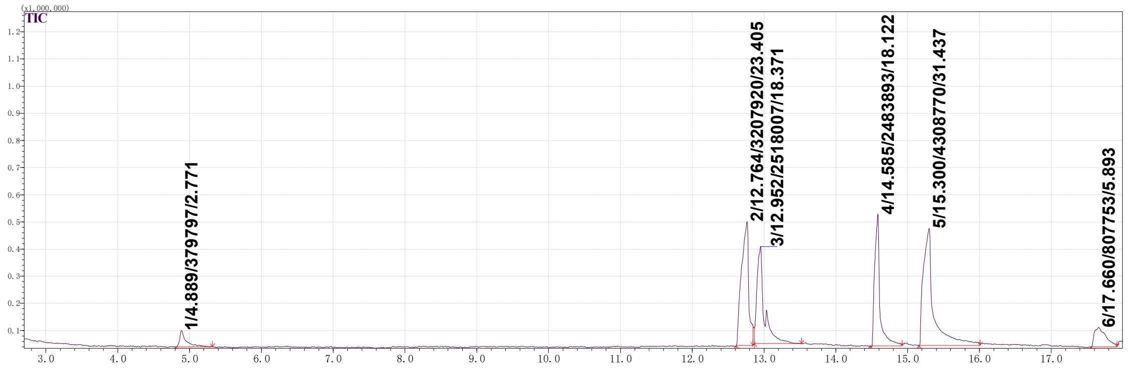

Figure S42. GC part of GC-MS for Entry 26, Table 1.

The peak 1 for t_R = 4.889 min with 2.771 % yield is unreacted toluene. The peak 2 for t_R = 12.764 min with 23.405 % yield is 4-chlorotoluene. The peak 3 for t_R = 12.952

min with 18.371 % yield is 2-chlorotoluene. The peak 4 for t_R = 14.585 min with 18.122 % yield is benzyl chloride. The peak 5 for t_R = 15.300 min with 31.437 % yield is benzyl alcohol. The peak 6 for t_R = 17.660 min with 5.893 % yield is 2,4-dichlorotoluene.

(27) Entry 27, Table 1

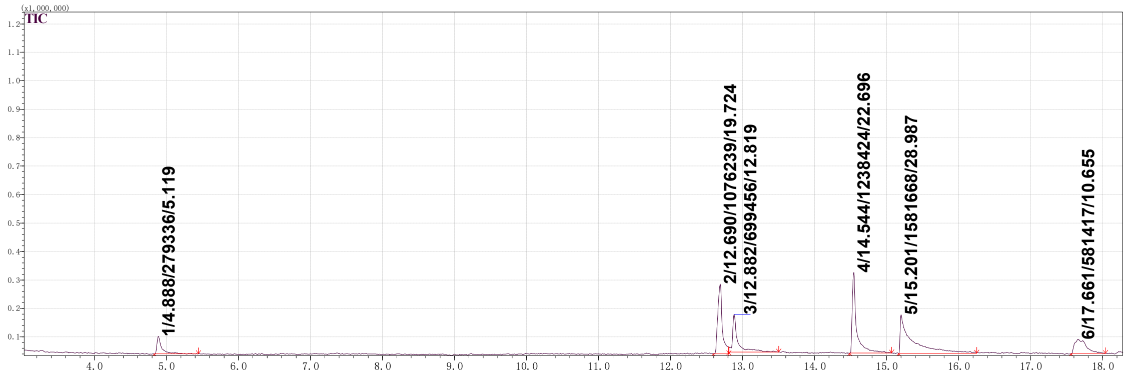

Figure S43. GC part of GC-MS for Entry 27, Table 1.

The peak 1 for t_R = 4.888 min with 5.119 % yield is unreacted toluene. The peak 2 for t_R = 12.690 min with 19.724 % yield is 4-chlorotoluene. The peak 3 for t_R = 12.882 min with 12.819% yield is 2-chlorotoluene. The peak 4 for t_R = 14.544 min with 22.696 % yield is benzyl chloride. The peak 5 for t_R = 15.201 min with 28.987 % yield is benzyl alcohol. The peak 6 for t_R = 17.661 min with 10.655 % yield is 2,4-dichlorotoluene.

(28) Entry 28, Table 1

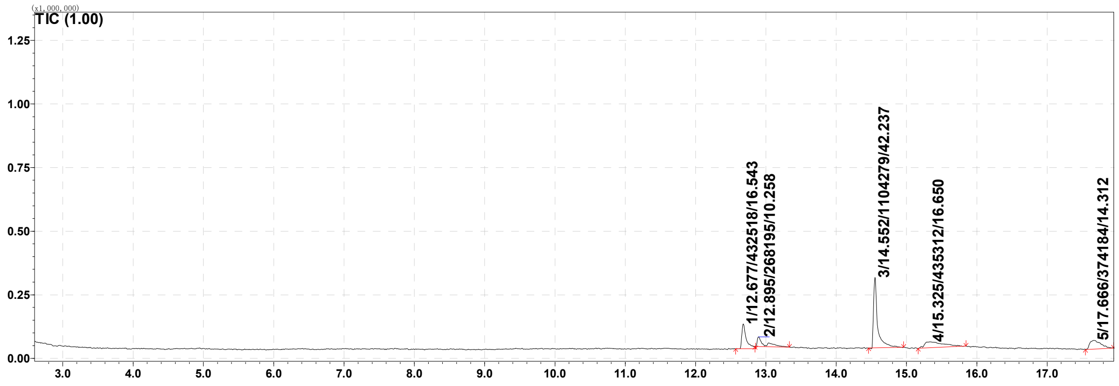

Figure S44. GC part of GC-MS for Entry 28, Table 1.

The peak 1 for t_R = 12.677 min with 16.543 % yield is 4-chlorotoluene. The peak 2 for t_R = 12.895 min with 10.258 % yield is 2-chlorotoluene. The peak 3 for t_R = 14.552 min with 42.237 % yield is benzyl chloride. The peak 4 for t_R = 15.325 min with 16.650 % yield is benzyl alcohol. The peak 5 for t_R = 17.666 min with 14.312 % yield is 2,4-dichlorotoluene.

20. Proposed Mechanism for the Chlorination of Fluorobenzene using H_2O_2 and $(NH_4)_2S_2O_8$ as Oxidants

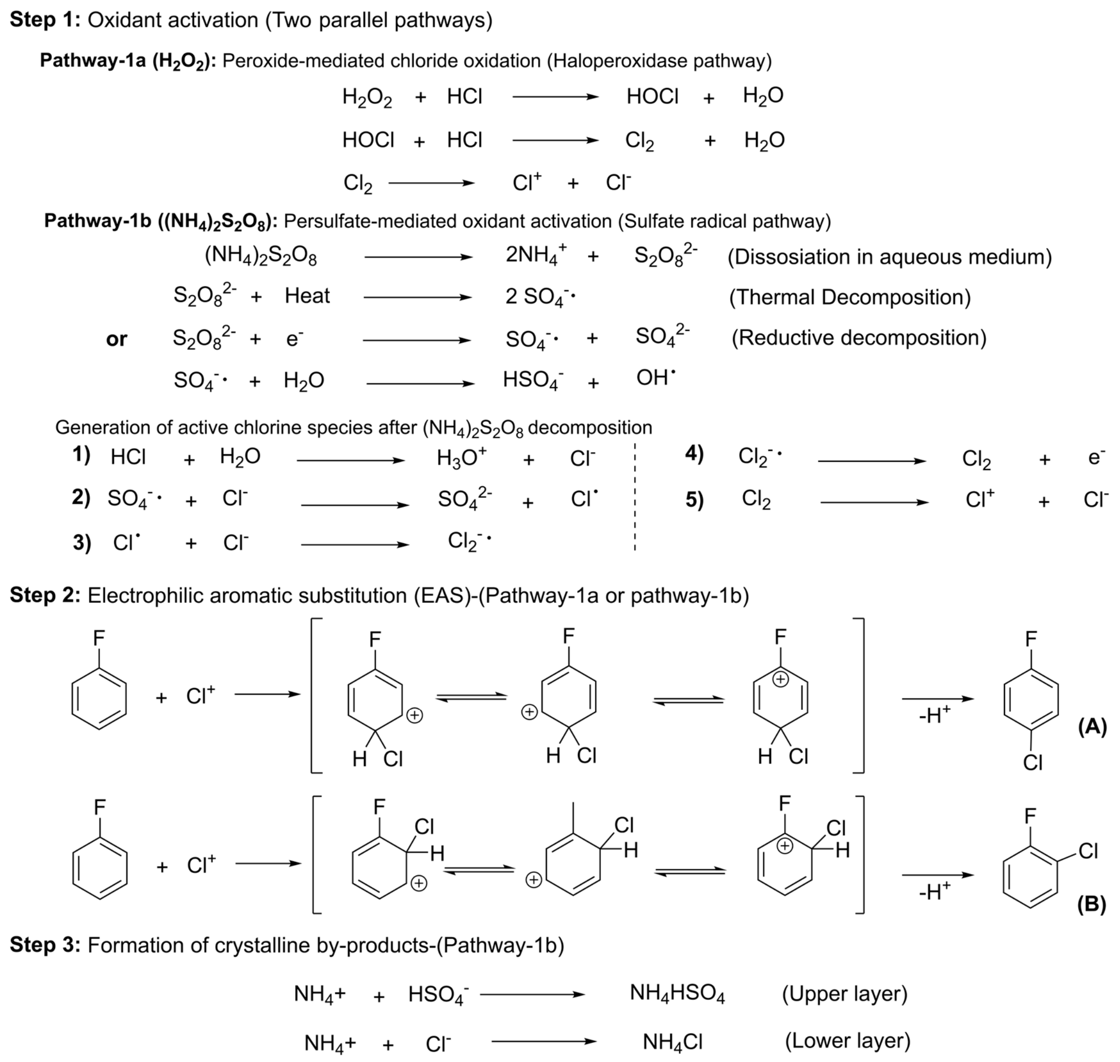

Figure S45. Proposed mechanism for the chlorination of fluorobenzene using H_2O_2 and $(NH_4)_2S_2O_8$ as oxidants.

21. GC-MS Examples for Table 2

(1) Entry 1, Table 2

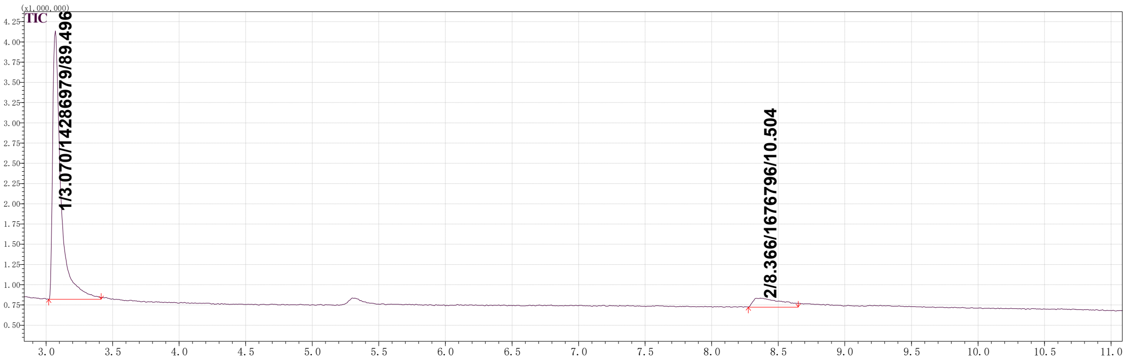

Figure S46. GC part of GC-MS for Entry 1, Table 2.

The peak 1 for t_R = 3.070 min with 89.496 % yield is:

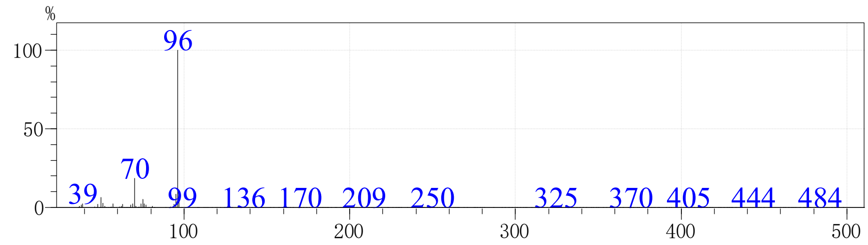

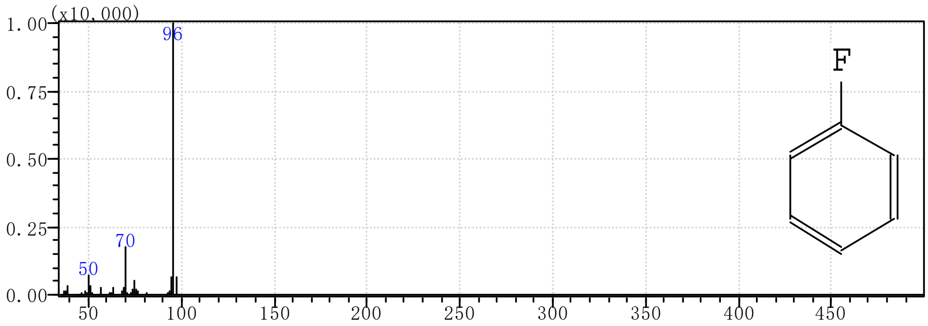

GC-MS: calcd. for C_6H_5F 96, found 96 (C_6H_5F). Table 2.

The peak 2 for t_R = 8.366 min with 10.504 % yield is:

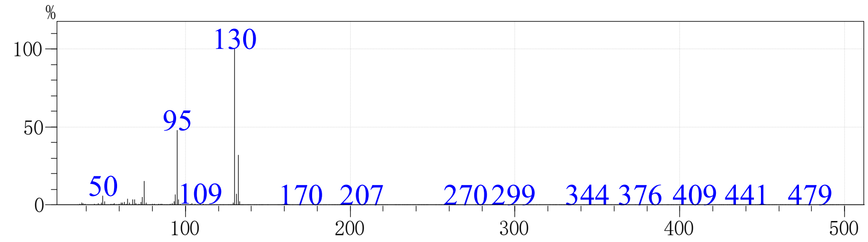

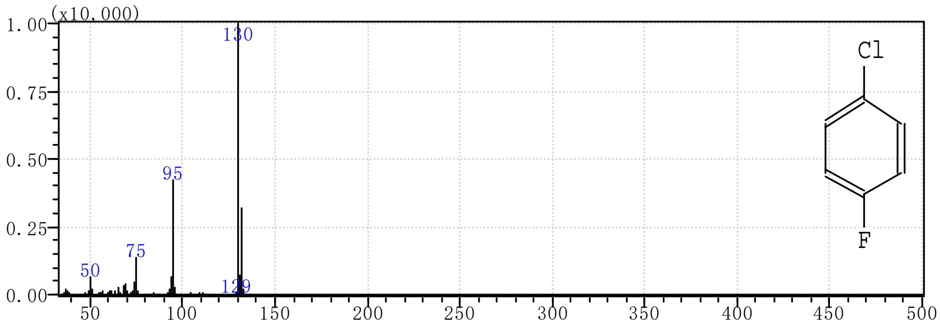

GC-MS: calcd. for C_6H_4ClF 130, found 130 (C_6H_4ClF). Table 2.

The peak 1 for t_R = 3.070 min with 89.496 % yield is unreacted fluorobenzene. The peak 2 for t_R = 8.366 min with 10.504 % yield is 4-chlorofluorobenzene.

(2) Entry 2, Table 2

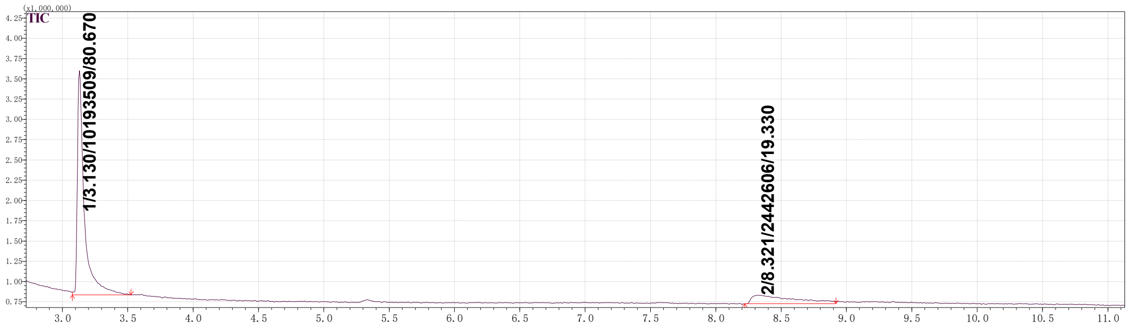

Figure S47. GC part of GC-MS for Entry 2, Table 2.

The peak 1 for t_R = 3.130 min with 80.670 % yield is unreacted fluorobenzene. The peak 2 for t_R = 8.321 min with 19.330 % yield is 4-chlorofluorobenzene.

(3) Entry 3, Table 2

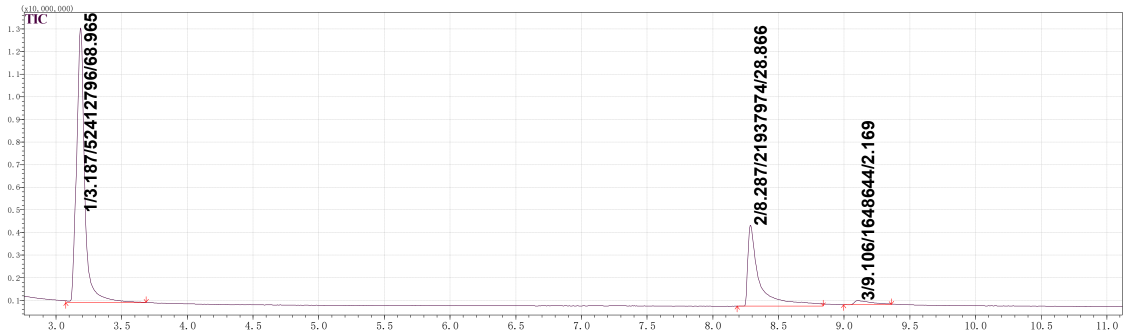

Figure S48. GC part of GC-MS for Entry 3, Table 2.

The peak 3 for t_R = 9.106 min with 2.169 % yield is:

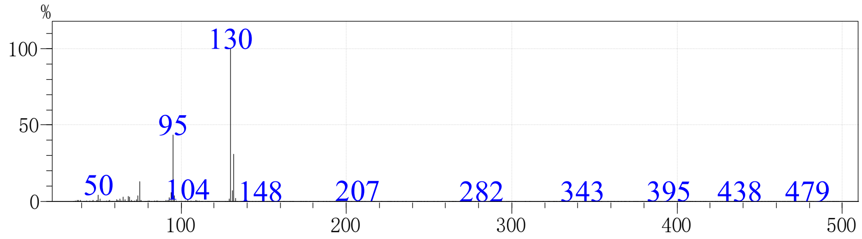

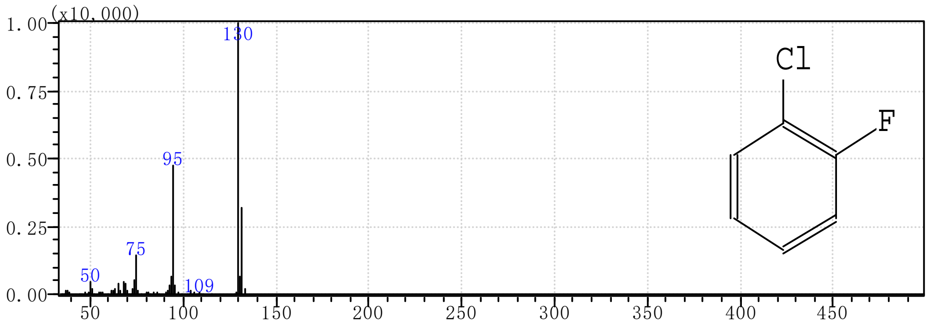

GC-MS: calcd. for C_6H_4ClF 130, found 130 (C_6H_4ClF). Table 2.

The peak 1 for t_R = 3.187 min with 68.965 % yield is unreacted fluorobenzene. The peak 2 for t_R = 8.287 min with 28.866 % yield is 4-chlorofluorobenzene. The peak 3 for t_R = 9.106 min with 2.169 % yield is 2-chlorofluorobenzene.

(4) Entry 4, Table 2

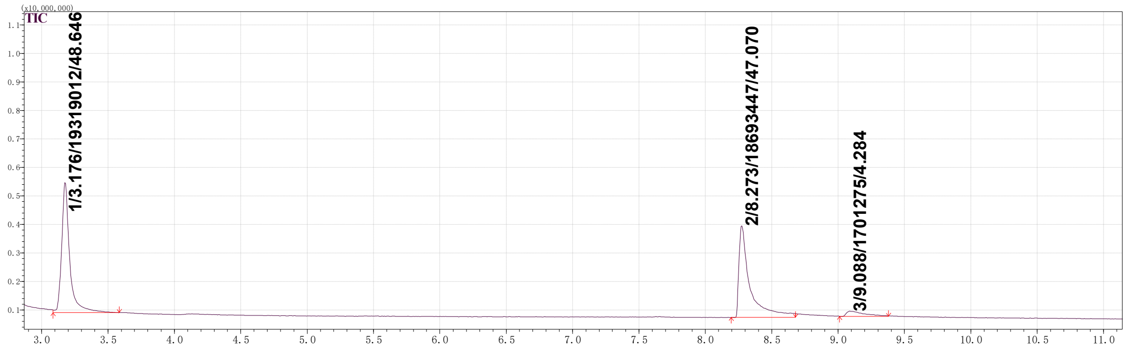

Figure S49. GC part of GC-MS for Entry 4, Table 2.

The peak 1 for t_R = 3.176 min with 48.646% yield is unreacted fluorobenzene. The peak 2 for t_R = 8.273 min with 47.070% yield is 4-chlorofluorobenzene. The peak 3 for t_R = 9.088 min with 4.284 % yield is 2-chlorofluorobenzene.

(5) Entry 5, Table 2

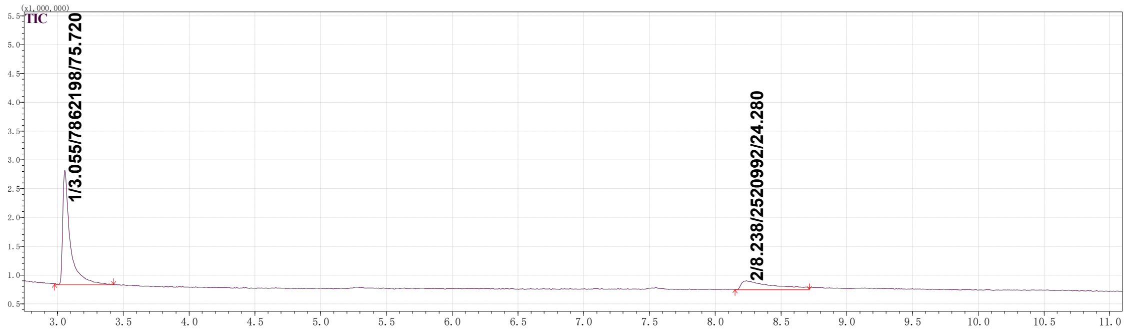

Figure S50. GC part of GC-MS for Entry 5, Table 2.

The peak 1 for t_R = 3.055 min with 75.720 % yield is unreacted fluorobenzene. The peak 2 for t_R = 8.238 min with 24.280 % yield is 4-chlorofluorobenzene.

(6) Entry 6, Table 2

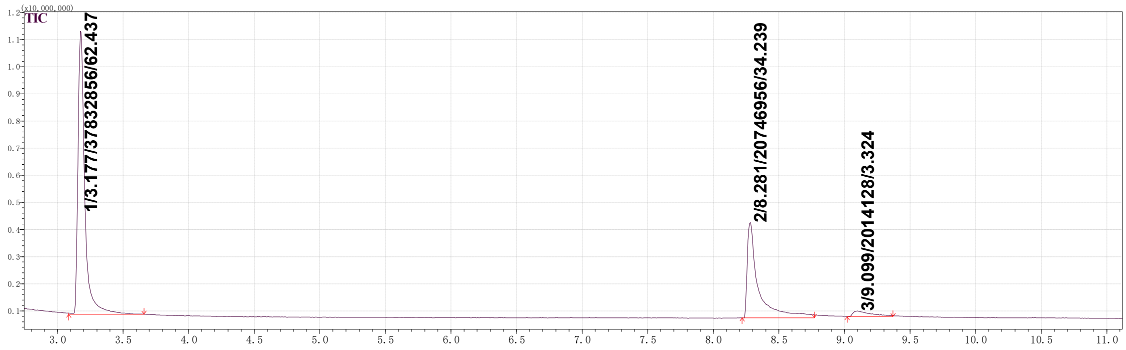

Figure S51. GC part of GC-MS for Entry 6, Table 2.

The peak 1 for t_R = 3.177min with 62.437 % yield is unreacted fluorobenzene. The peak 2 for t_R = 8.281 min with 34.239 % yield is 4-chlorofluorobenzene. The peak 3 for t_R = 9.099 min with 3.324 % yield is 2-chlorofluorobenzene.

(7) Entry 7, Table 2

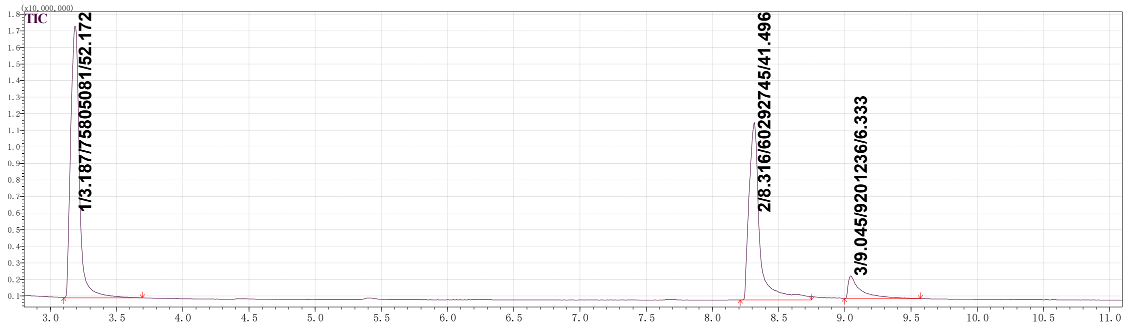

Figure S52. GC part of GC-MS for Entry 7, Table 2.

The peak 1 for t_R = 3.187 min with 52.172 % yield is unreacted fluorobenzene. The peak 2 for t_R = 8.316 min with 41.496 % yield is 4-chlorofluorobenzene. The peak 3 for t_R = 9.045 min with 6.333 % yield is 2-chlorofluorobenzene.

(8) Entry 8, Table 2

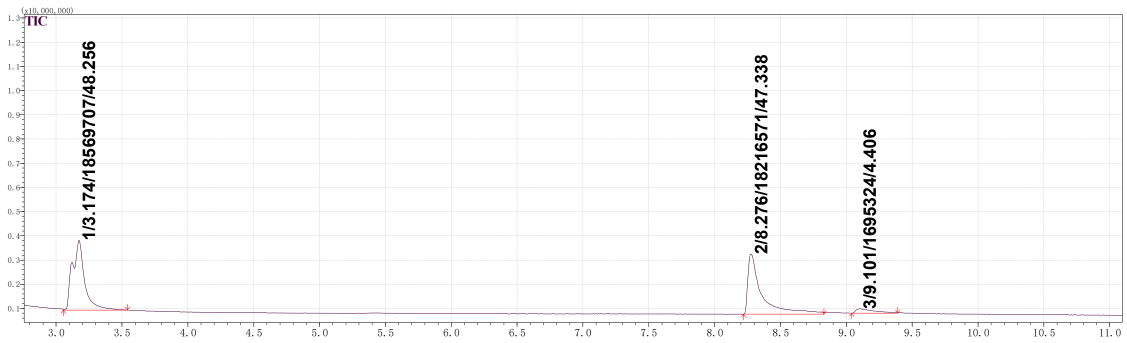

Figure S53. GC part of GC-MS for Entry 8, Table 2.

The peak 1 for t_R = 3.174 min with 48.256% yield is unreacted fluorobenzene. The peak 2 for t_R = 8.276 min with 47.338% yield is 4-chlorofluorobenzene. The peak 3 for t_R = 9.101 min with 4.406 % yield is 2-chlorofluorobenzene.

(9) Entry 9, Table 2

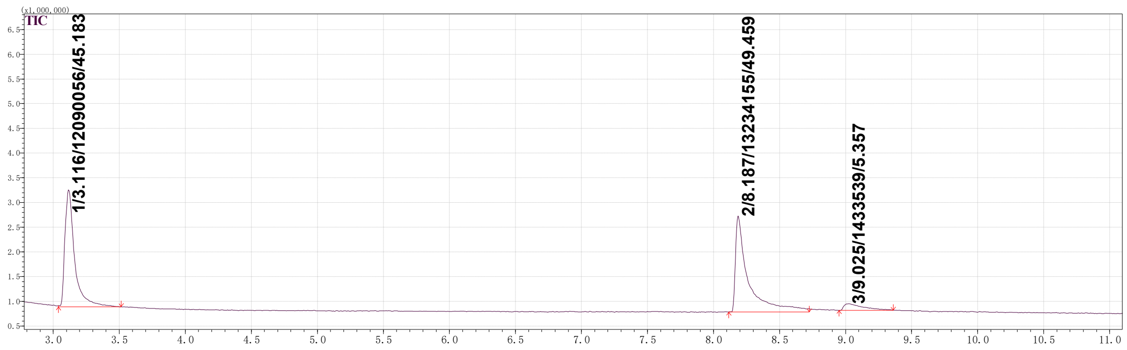

Figure S54. GC part of GC-MS for entry 9, table 2.

The peak 1 for t_R = 3.116 min with 45.183 % yield is unreacted fluorobenzene. The peak 2 for t_R = 8.187 min with 49.459 % yield is 4-chlorofluorobenzene. The peak 3 for t_R = 9.025 min with 5.357 % yield is 2-chlorofluorobenzene.

(10) Entry 10, Table 2

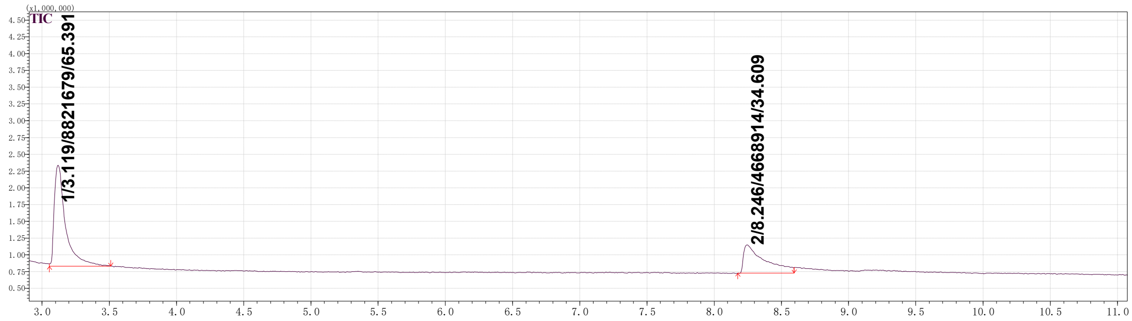

Figure S55. GC part of GC-MS for Entry 10, Table 2.

The peak 1 for t_R = 3.119 min with 65.391 % yield is unreacted fluorobenzene. The peak 2 for t_R = 8.246 min with 34.609 % yield is 4-chlorofluorobenzene.

22. Proposed Mechanism for the Chlorination of Bromobenzen using H_2O_2 as an Oxidant

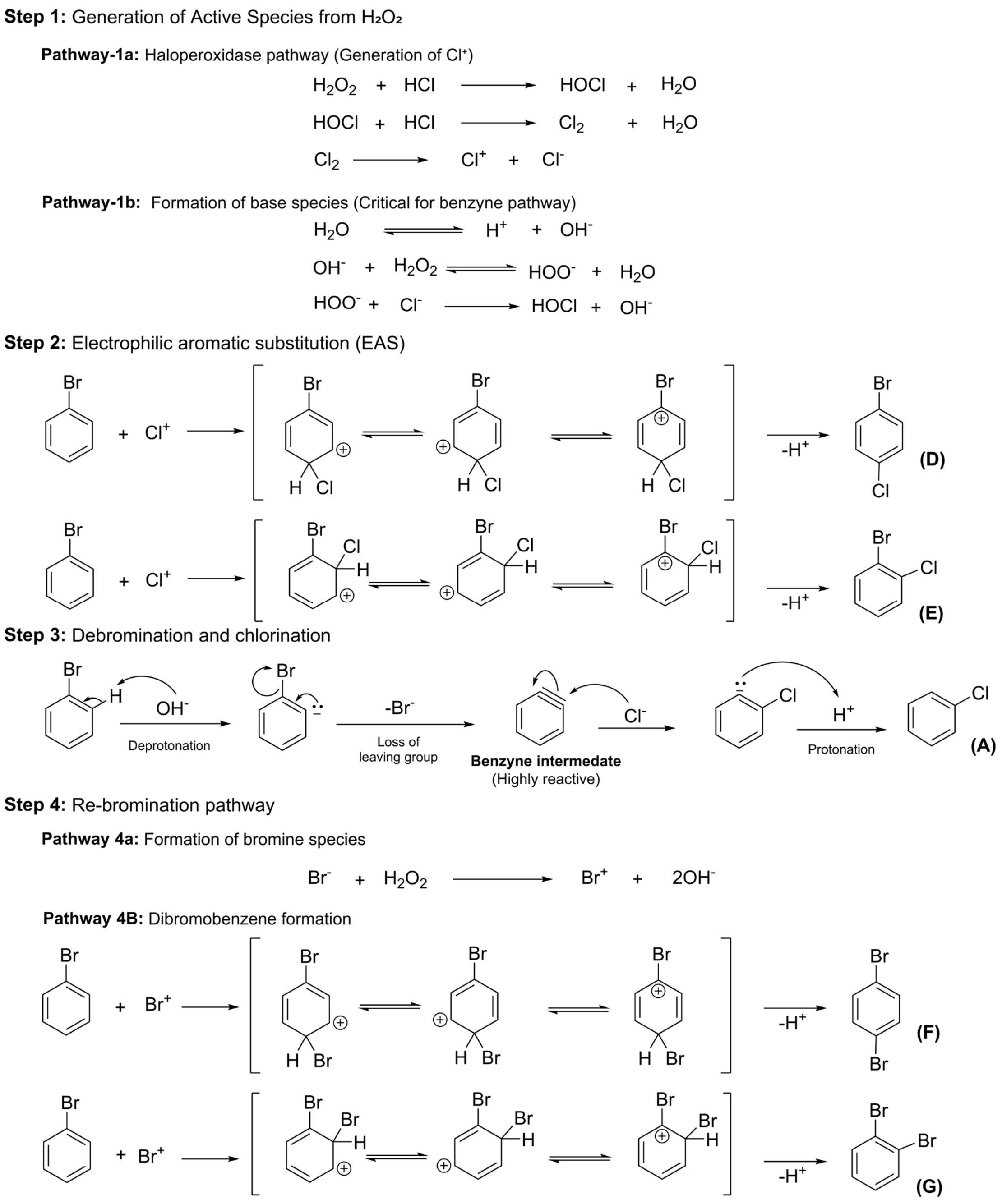

Figure S56. Proposed mechanism for the chlorination of bromobenzene using H_2O_2 as an oxidant.

23. GC-MS Examples for Table 3

(1) Entry 1, Table 3

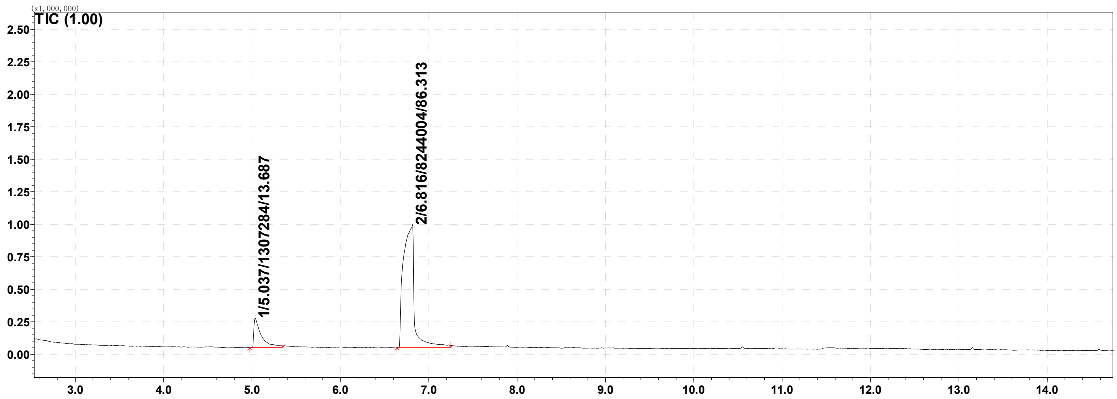

Figure S57. GC part of GC-MS for Entry 1, Table 3.

The peak 1 for t_R = 5.037 min with 13.687 % yield is:

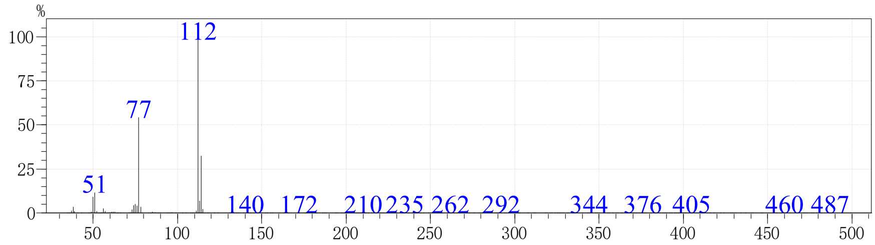

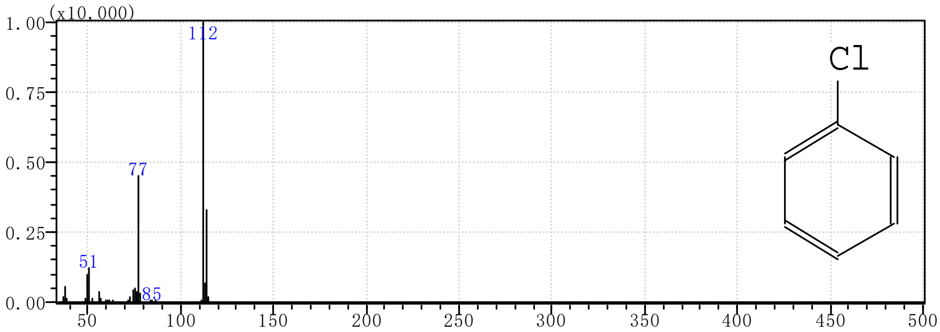

GC-MS: calcd. for C_6H_5Cl 112, found 112 (C_6H_5Cl). Table 3.

The peak 2 for t_R = 6.816 min with 86.313 % yield is:

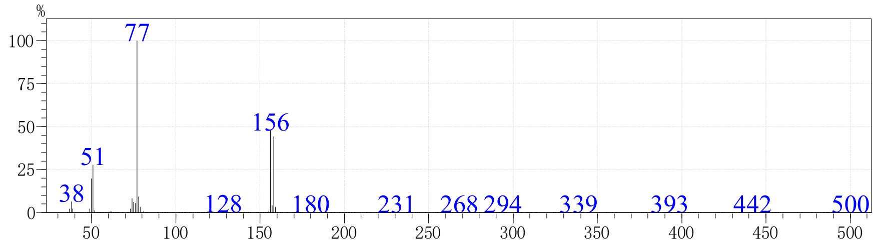

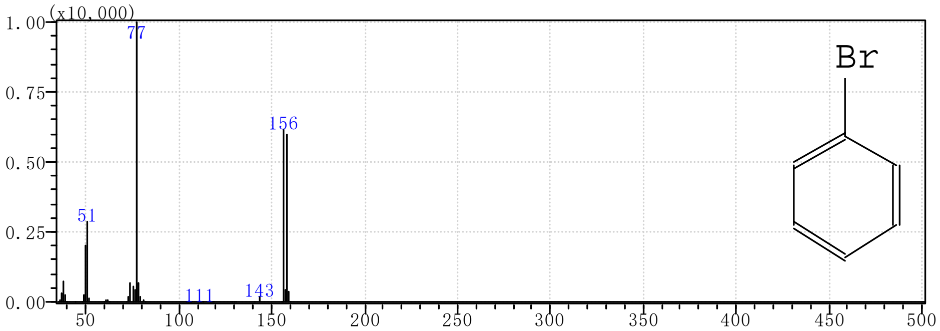

GC-MS: calcd. for C_6H_5Br 156, found 156 (C_6H_5Br). Table 3.

The peak 1 for t_R = 5.037 min with 13.687 % yield is chlorobenzene. The peak 2 for t_R = 6.816 min with 86.313 % yield is unreacted bromobenzene.

(2) Entry 2, Table 3

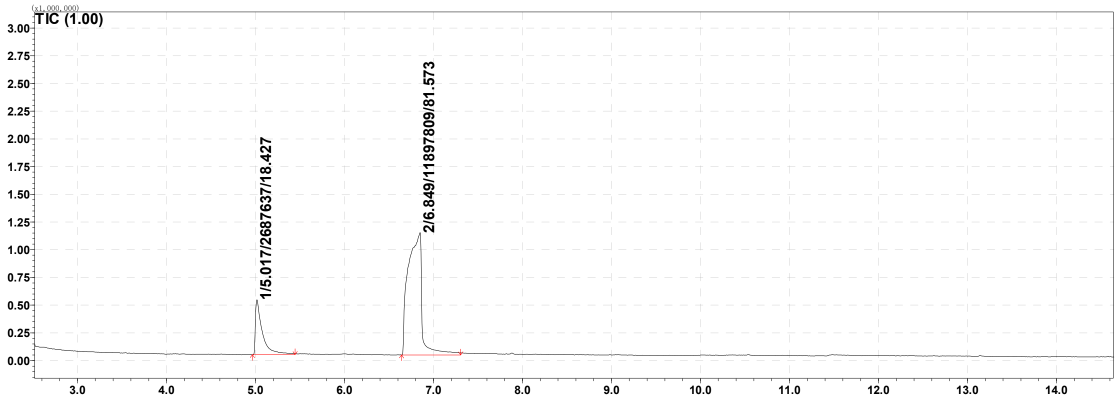

Figure S58. GC part of GC-MS for Entry 2, Table 3.

The peak 1 for t_R = 5.017 min with 18.427 % yield is chlorobenzene. The peak 2 for t_R = 6.849 min with 81.573 % yield is unreacted bromobenzene.

(3) Entry 3, Table 3

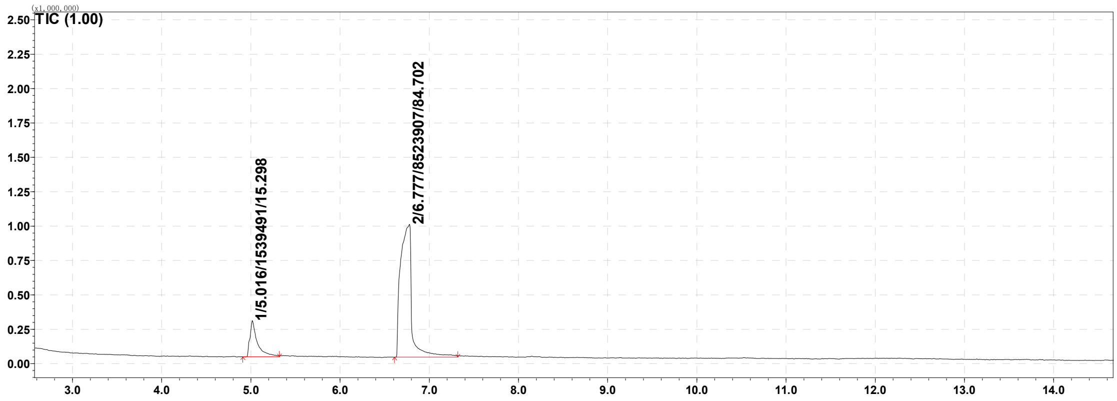

Figure S59. GC part of GC-MS for Entry 3, Table 3.

The peak 1 for t_R = 5.016 min with 15.298 % yield is chlorobenzene. The peak 2 for t_R = 6.777 min with 84.702 % yield is unreacted bromobenzene.

(4) Entry 4, Table 3

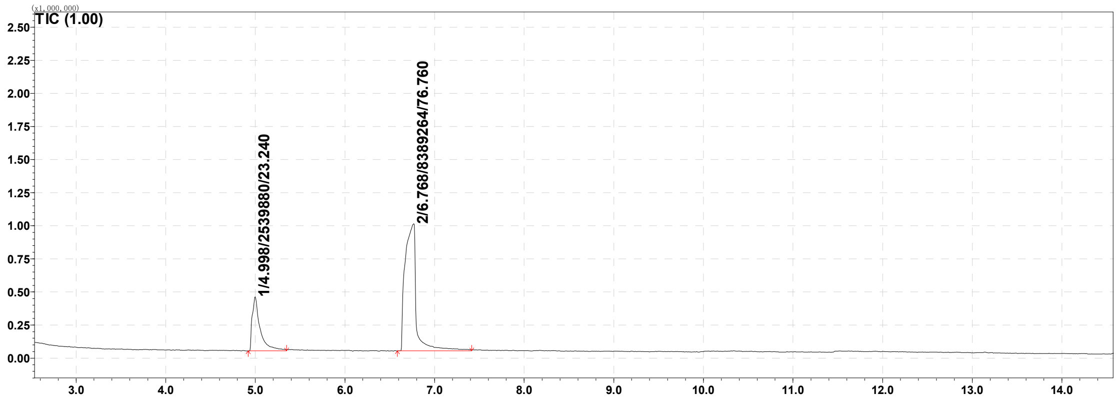

Figure S60. GC part of GC-MS for Entry 4, Table 3.

The peak 1 for t_R = 4.998 min with 23.240 % yield is chlorobenzene. The peak 2 for t_R = 6.768 min with 76.760 % yield is unreacted bromobenzene.

(5) Entry 5, Table 3

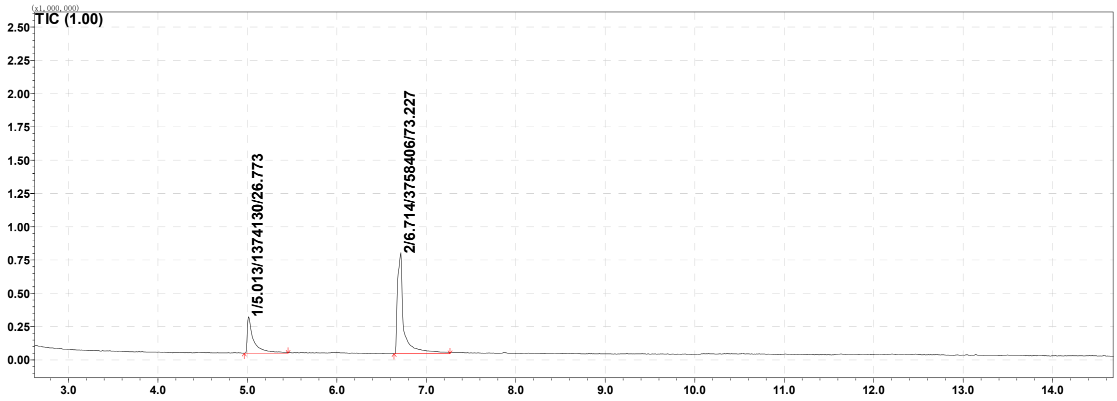

Figure S61. GC part of GC-MS for Entry 5, Table 3.

The peak 1 for t_R = 5.013 min with 26.773 % yield is chlorobenzene. The peak 2 for t_R = 6.714 min with 73.227 % yield is unreacted bromobenzene.

(6) Entry 6, Table 3

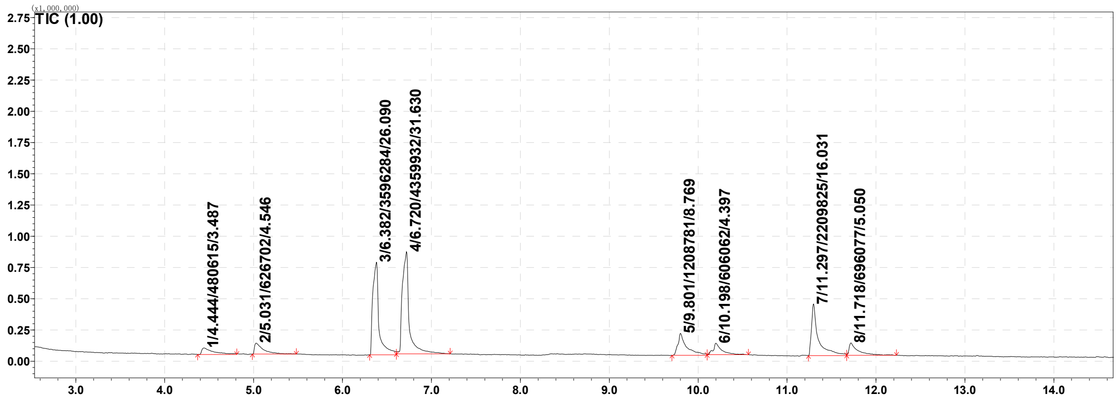

Figure S62. GC part of GC-MS for Entry 6, Table 3.

The peak 5 for t_R = 9.801 min with 8.769 % yield is:

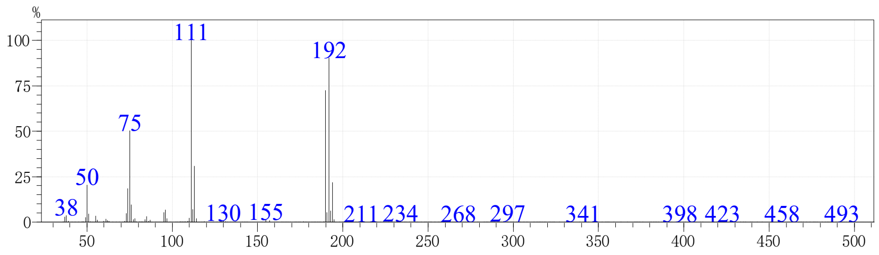

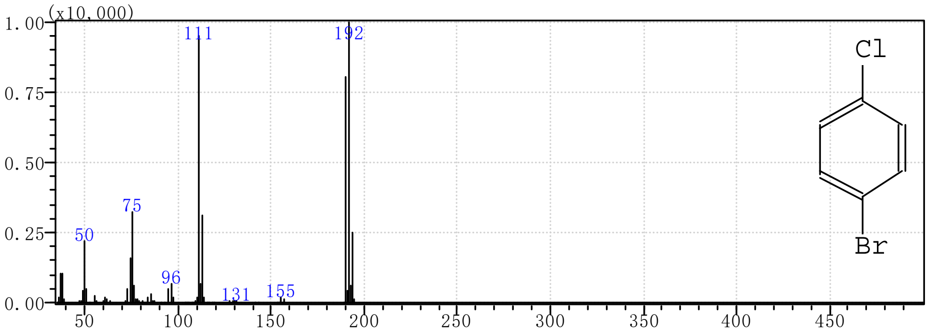

GC-MS: calcd. for C_6H_4BrCl 192, found 192 (C_6H_4BrCl). Table 3.

The peak 6 for t_R = 10.198 min with 4.397 % yield is:

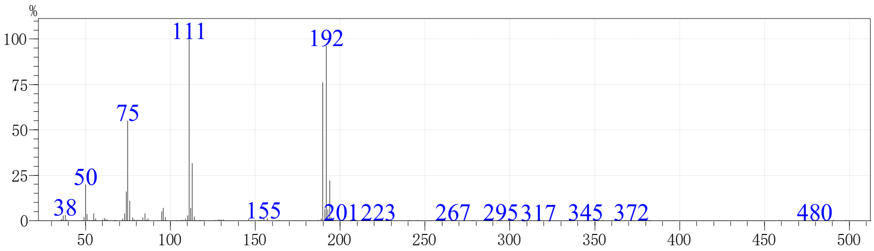

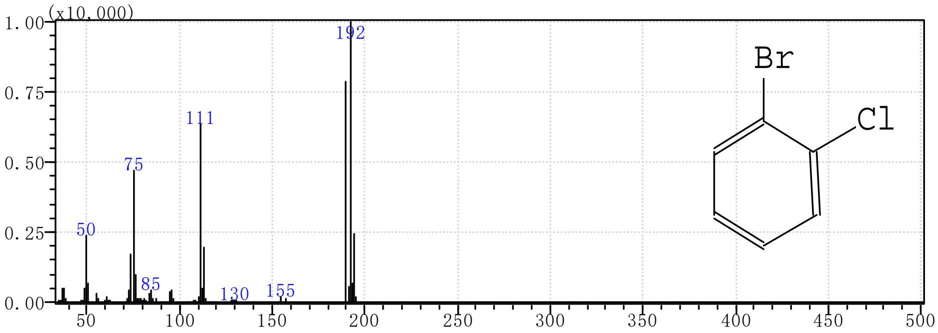

GC-MS: calcd. for C_6H_4BrCl 192, found 192 (C_6H_4BrCl). Table 3.

The peak 7 for t_R = 11.297 min with 16.031 % yield is:

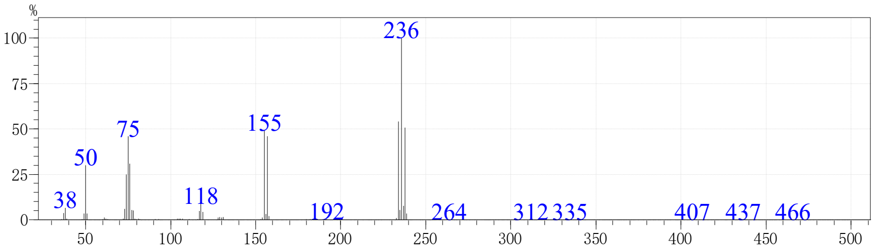

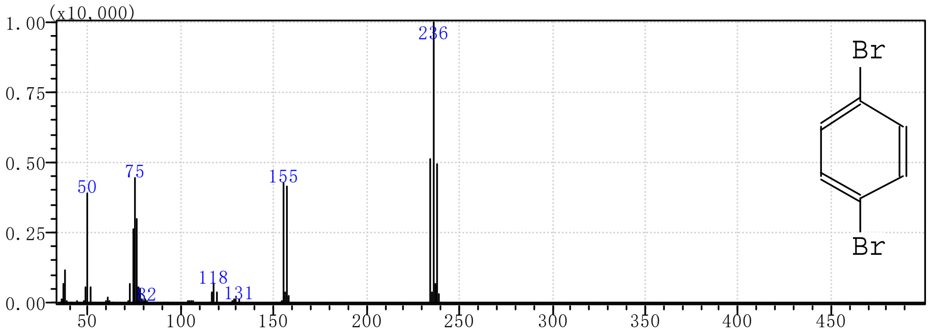

GC-MS: calcd. for $C_6H_4Br_2$ 236, found 236 ($C_6H_4Br_2$). Table 3.

The peak 8 for t_R = 11.718 min with 5.050 % yield is:

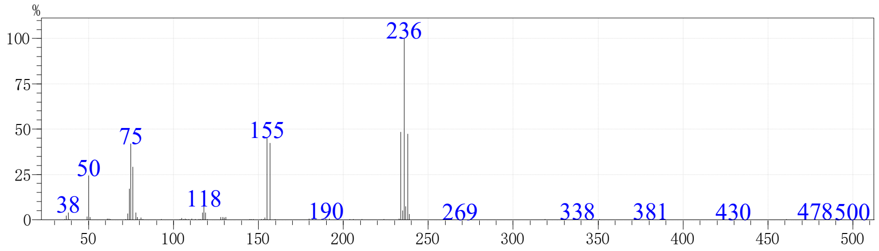

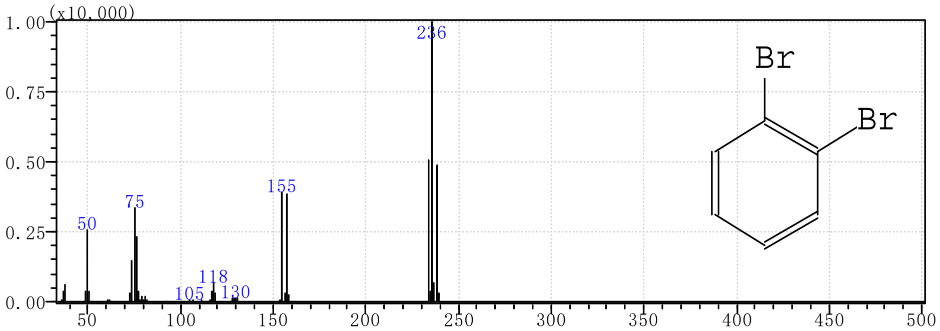

GC-MS: calcd. for $C_6H_4Br_2$ 236, found 236 ($C_6H_4Br_2$). Table 3.

The peak 1 for t_R = 4.444 min with 3.487 % yield and the peak 2 for t_R = 5.031 min with 4.546 % yield, both are chlorobenzene. The peak 3 for t_R = 6.382 min with 26.090 % yield and the peak 4 for t_R = 6.720 min with 31.630 % yield, both are unreacted bromobenzene. The peak 5 for t_R = 9.801 min with 8.769 % yield is 1-bromo-4-chlorobenzene. The peak 6 for t_R = 10.198 min with 4.397 % yield is 1-bromo-2-chlorobenzene. The peak 7 for t_R = 11.297 min with 16.031 % yield is 1,4-

dibromobenzene. The peak 8 for t_R = 11.718 min with 5.050 % yield is 1,2-dibromobenzene.

(7) Entry 7, Table 3

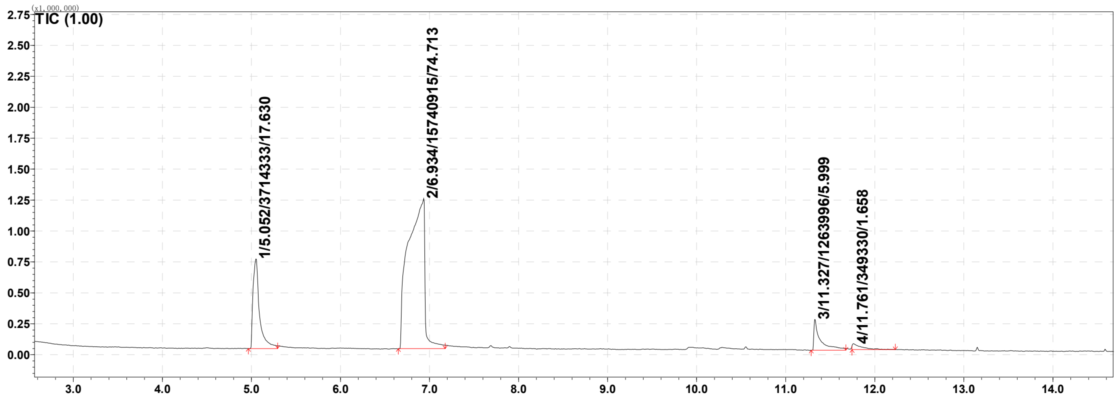

Figure S63. GC part of GC-MS for Entry 7, Table 3.

The peak 1 for t_R = 5.052 min with 17.630 % yield is chlorobenzene. The peak 2 for t_R = 6.934 min with 74.713 % yield is unreacted bromobenzene. The peak 3 for t_R = 11.327 min with 5.999 % yield is 1,4-dibromobenzene. The peak 4 for t_R = 11.761 min with 1.658 % yield is 1,2-dibromobenzene.

(8) Entry 8, Table 8

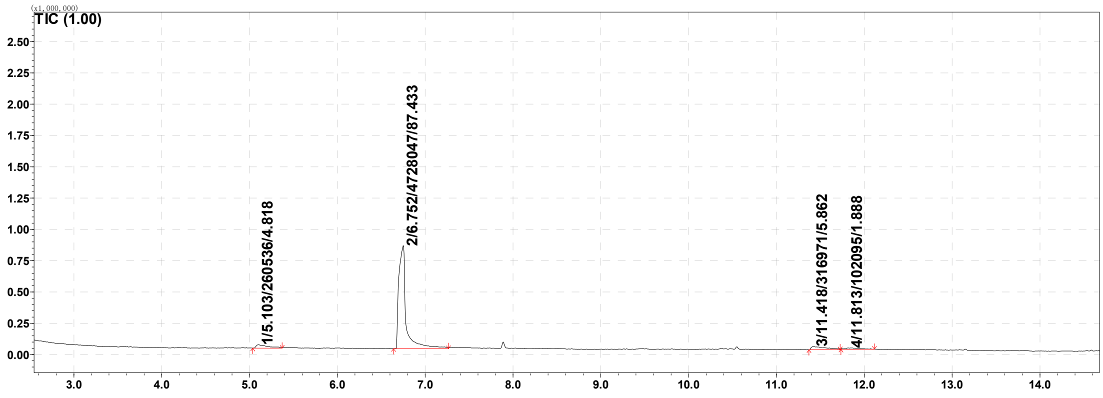

Figure S64. GC part of GC-MS for Entry 8, Table 3.

The peak 1 for t_R = 5.103 min with 4.818 % yield is chlorobenzene. The peak 2 for t_R = 6.752 min with 87.433 % yield is unreacted bromobenzene. The peak 3 for t_R = 11.418 min with 5.862 % yield is1,4-dibromobenzene. The peak 4 for t_R = 11.813 min with 1.888 % yield is1,2-dibromobenzene.

(9) Entry 9, Table 3

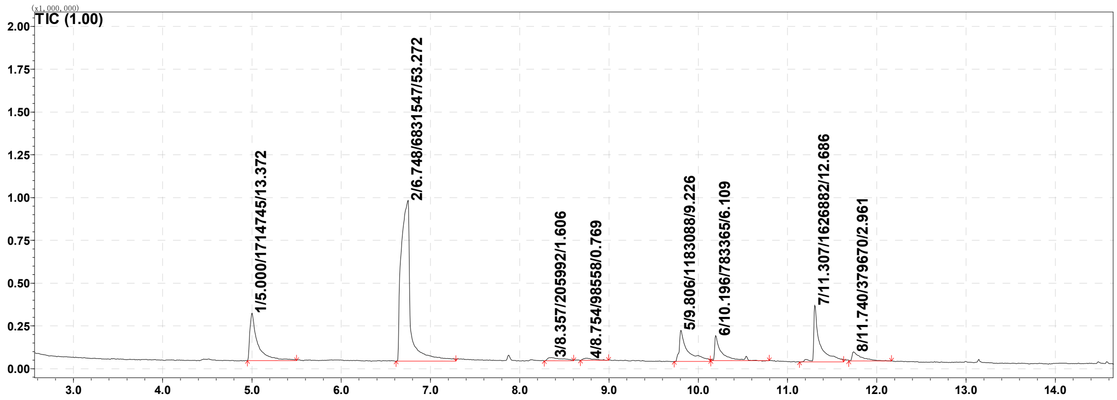

Figure S65. GC part of GC-MS for Entry 9, Table 3.

The peak 3 for t_R = 8.357 min with 1.606 % yield is:

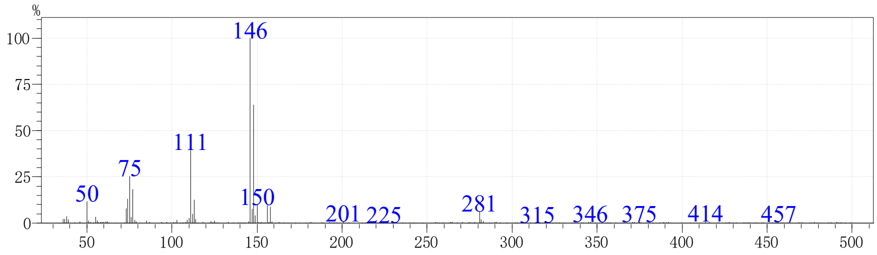

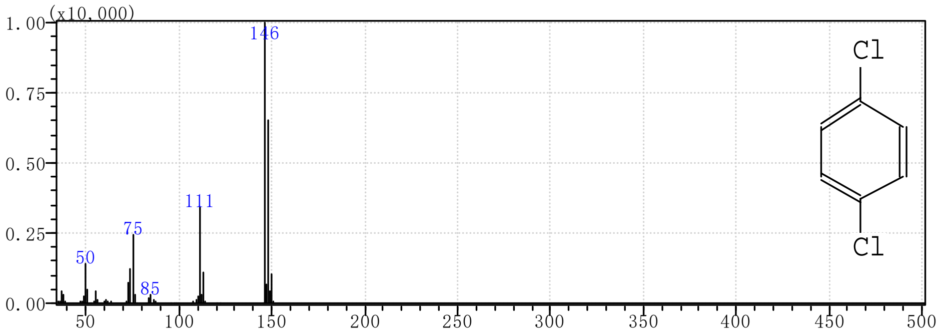

GC-MS: calcd. for $C_6H_4Br_2$ 146, found 146 ($C_6H_4Cl_2$). Table 3.

The peak 4 for t_R = 8.754 min with 0.769 % yield is:

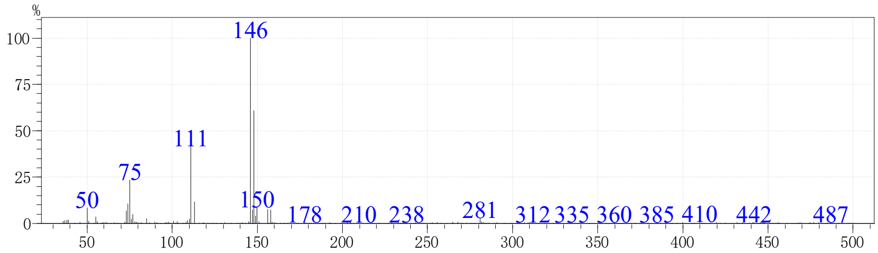

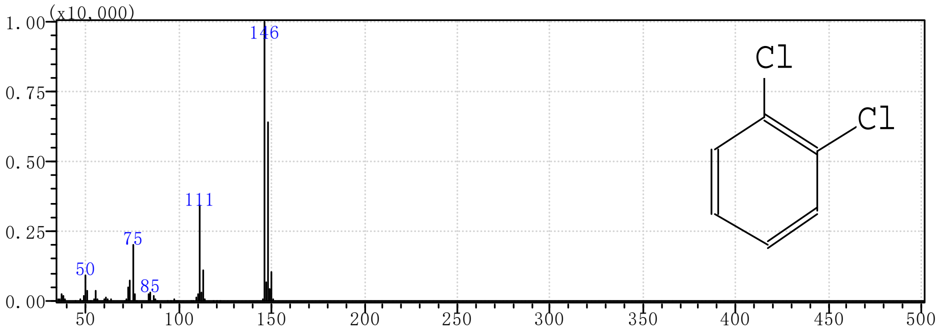

GC-MS: calcd. for $C_6H_4Br_2$ 146, found 146 ($C_6H_4Cl_2$). Table 3.

The peak 1 for t_R = 5.000 min with 13.372 % yield is chlorobenzene. The peak 2 for t_R = 6.748 min with 53.272 % yield is unreacted bromobenzene. The peak 3 for t_R = 8.357 min with 1.606 % yield is 1,4-dichlorobenzene. The peak 4 for t_R = 8.754 min with 0.769 % yield is 1,2-dichlorobenzene. The peak 5 for t_R = 9.806 min with 9.226 % yield is 1-bromo-4-chlorobenzene. The peak 6 for t_R = 10.196 min with 6.109 % yield is 1-bromo-2-chlorobenzene. The peak 7 for t_R = 11.307 min with 12.686 % yield is 1,4-dibromobenzene. The peak 8 for t_R = 11.740 min with 2.961 % yield is 1,2-dibromobenzene.

(10) Entry 10, Table 3

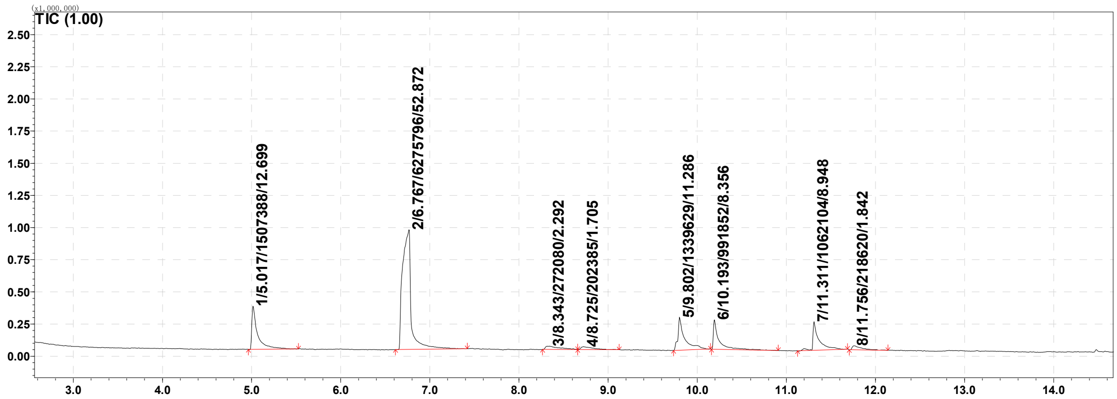

Figure S66. GC part of GC-MS for Entry 10, Table 3.

The peak 1 for t_R = 5.017 min with 12.699 % yield is chlorobenzene. The peak 2 for t_R = 6.767 min with 52.872 % yield is unreacted bromobenzene. The peak 3 for t_R = 8.343 min with 2.292 % yield is 1,4-dichlorobenzene. The peak 4 for t_R = 8.725 min with 1.705 % yield is 1,2-dichlorobenzene. The peak 5 for t_R = 9.802 min with 11.286 % yield is 1-bromo-4-chlorobenzene. The peak 6 for t_R = 10.193 min with 8.356 % yield is 1-bromo-2-chlorobenzene. The peak 7 for t_R = 11.311 min with 8.948 % yield is 1,4-dibromobenzene. The peak 8 for t_R = 11.756 min with 1.842 % yield is 1,2-dibromobenzene.

(11) Entry 11, Table 3

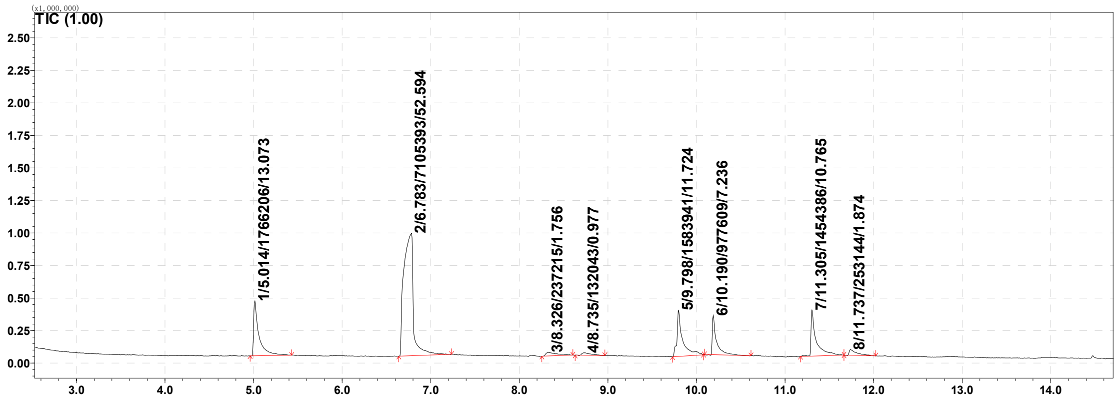

Figure S67. GC part of GC-MS for Entry 11, Table 3.

The peak 1 for t_R = 5.014 min with 13.073 % yield is chlorobenzene. The peak 2 for t_R = 6.783 min with 52.594 % yield is unreacted bromobenzene. The peak 3 for t_R = 8.326 min with 1.756 % yield is 1,4-dichlorobenzene. The peak 4 for t_R = 8.735 min with 0.977 % yield is 1,2-dichlorobenzene. The peak 5 for t_R = 9.798 min with 11.724 % yield is 1-bromo-4-chlorobenzene. The peak 6 for t_R = 10.190 min with 7.236 % yield is 1-bromo-2-chlorobenzene. The peak 7 for t_R = 11.305 min with 10.765 % yield is 1,4-dibromobenzene. The peak 8 for t_R = 11.737 min with 1.874 % yield is 1,2-dibromobenzene.

(12) Entry 12, Table 3

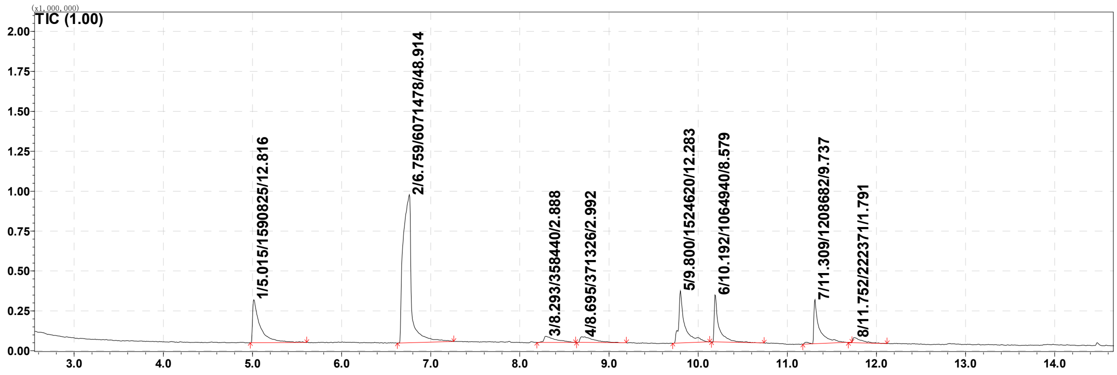

Figure S68. GC part of GC-MS for Entry 12, Table 3.

The peak 1 for t_R = 5.015 with 12.816 % yield is chlorobenzene. The peak 2 for t_R = 6.759 min with 48.914 % yield is unreacted bromobenzene. The peak 3 for t_R = 8.293 min with 2.888 % yield is 1,4-dichlorobenzene. The peak 4 for t_R = 8.695 min with 2.992 % yield is 1,2-dichlorobenzene. The peak 5 for t_R = 9.800 with 12.283 % yield is 1-bromo-4-chlorobenzene. The peak 6 for t_R = 10.192 min with 8.579 % yield is 1-

bromo-2-chlorobenzene. The peak 7 for t_R = 11.309 min with 9.737 % yield is 1,4-dibromobenzene. The peak 8 for t_R = 11.752 min with 1.791 % yield is 1,2-dibromobenzene.

(13) Entry 13, Table 3

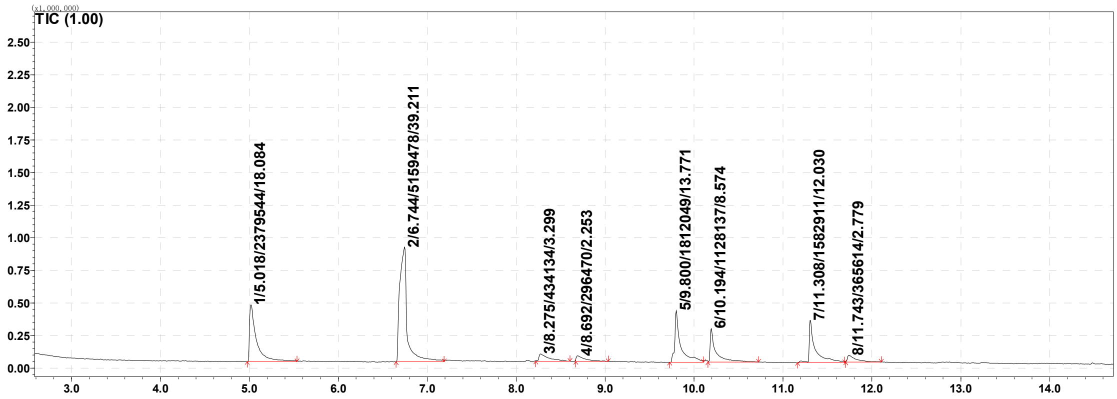

Figure S69. GC part of GC-MS for Entry 13, Table 3.

The peak 1 for t_R = 5.018 with 18.084% yield is chlorobenzene. The peak 2 for t_R = 6.744 min with 39.21 % yield is unreacted bromobenzene. The peak 3 for t_R = 8.275 min with 3.299 % yield is 1,4-dichlorobenzene. The peak 4 for t_R = 8.692 min with 2.253 % yield is 1,2-dichlorobenzene. The peak 5 for t_R = 9.800 min with 13.771 % yield is 1-bromo-4-chlorobenzene. The peak 6 for t_R = 10.194 min with 8.574 % yield is 1-bromo-2-chlorobenzene. The peak 7 for t_R = 11.308 min with 12.030 % yield is 1,4-dibromobenzene. The peak 8 for t_R = 11.743min with 2.779% yield is 1,2-dibromobenzene.

(14) Entry 14, Table 3

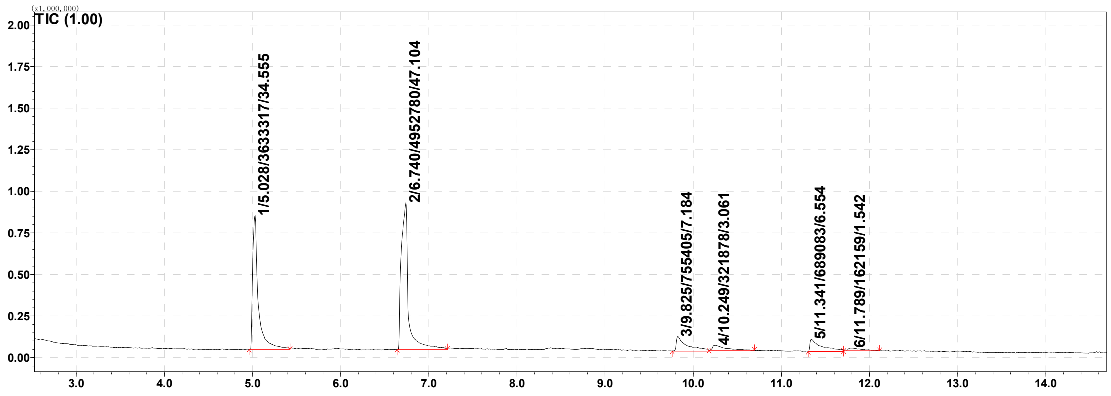

Figure S70. GC part of GC-MS for Entry 14, Table 3.

The peak 1 for t_R = 5.028 min with 34.55 % yield is chlorobenzene. The peak 2 for t_R = 6.740 min with 47.104 % yield is unreacted bromobenzene. The peak 3 for t_R = 9.825 min with 7.185 % yield is 1-bromo-4-chlorobenzene. The peak 4 for t_R = 10.249 min with 3.061 % yield is 1-bromo-2-chlorobenzene. The peak 5 for t_R = 11.341 with 6.554 % yield is 1,4-dibromobenzene. The peak 6 for t_R = 11.789 min with 1.542 % yield is 1,2-dibromobenzene.

(15) Entry 15, Table 3

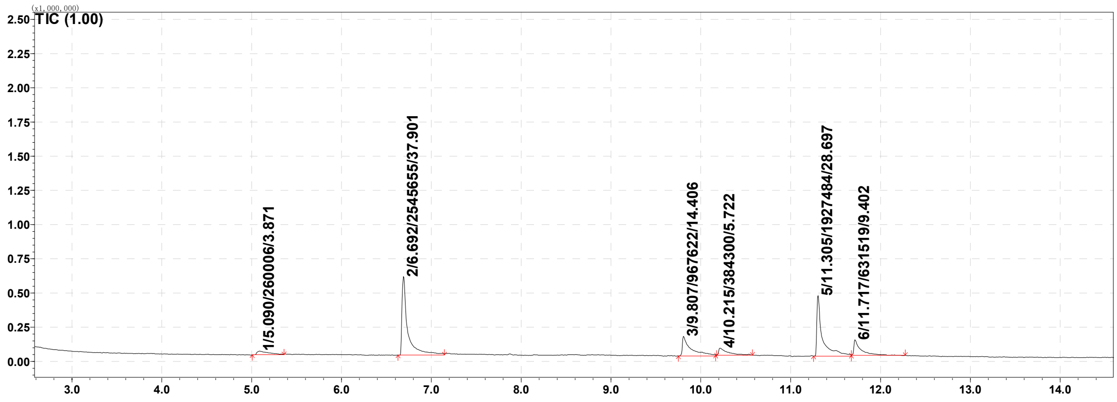

Figure S71. GC part of GC-MS for Entry 15, Table 3.

The peak 1 for t_R = 5.090 min with 3.871 % yield is chlorobenzene. The peak 2 for t_R = 6.692 min with 7.901 % yield is unreacted bromobenzene. The peak 3 for t_R = 9.807

min with 14.406 % yield is 1-bromo-4-chlorobenzene. The peak 4 for t_R = 10.215 min with 5.722 % yield is 1-bromo-2-chlorobenzene. The peak 5 for t_R = 11.305 min with 28.697 % yield is 1,4-dibromobenzene. The peak 6 for t_R = 11.717 min with 9.402 % yield is 1,2-dibromobenzene.

(16) Entry 16, Table 3

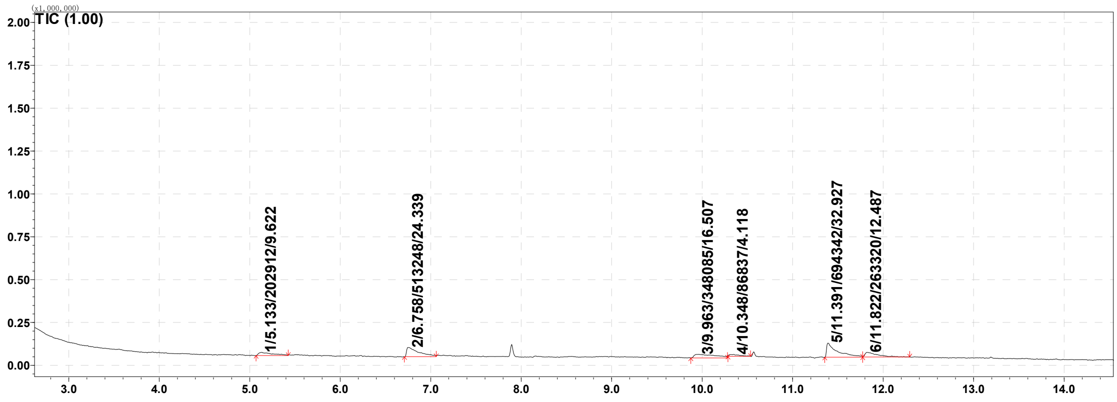

Figure S72. GC part of GC-MS for Entry 16, Table 3.

The peak 1 for t_R = 5.133 min with 9.622 % yield is chlorobenzene. The peak 2 for t_R = 6.758 min with 24.339 % yield is unreacted bromobenzene. The peak 3 for t_R = 9.963 min with 16.507 % yield is 1-bromo-4-chlorobenzene. The peak 4 for t_R = 10.348 min with 4.118 % yield is 1-bromo-2-chlorobenzene. The peak 5 for t_R = 11.391 min with 32.927 % yield is 1,4-dibromobenzene. The peak 6 for t_R = 11.822 min with 12.487 % yield is 1,2-dibromobenzene.

24. Proposed Mechanism for the Chlorination of Iodobenzene using H_2O_2 and $(NH_4)_2S_2O_8$ as Oxidants

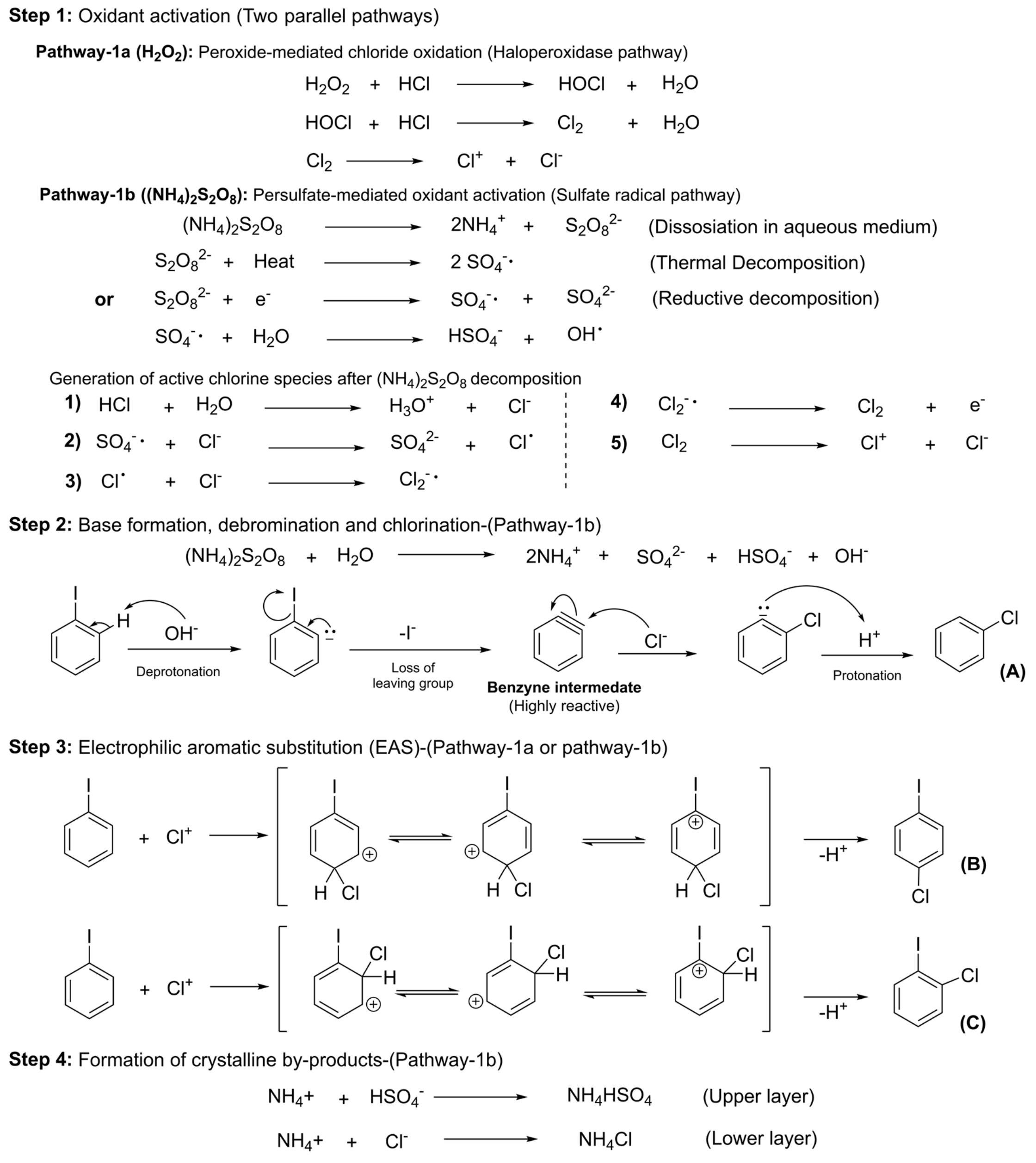

Figure S73. Proposed mechanism for the chlorination of iodobenzene using H_2O_2 and $(NH_4)_2S_2O_8$ as oxidants.

25. GC-MS Examples for Table 4

(1) Entry 1, Table 4

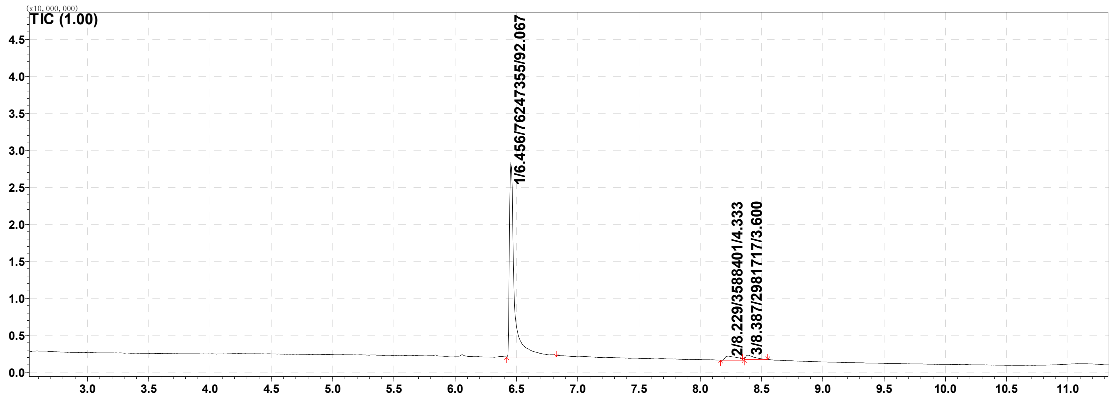

Figure S74. GC part of GC-MS for Entry 1, Table 4.

The peak 1 for t_R = 6.456 min with 92.067 % yield is:

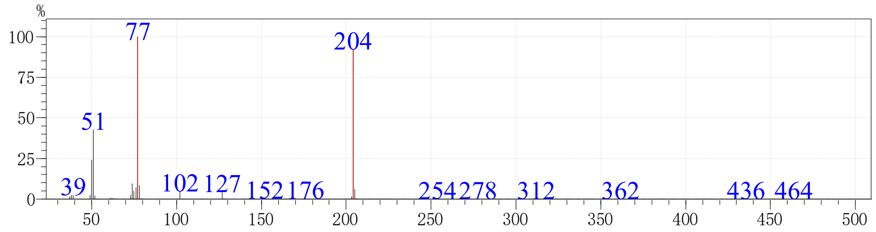

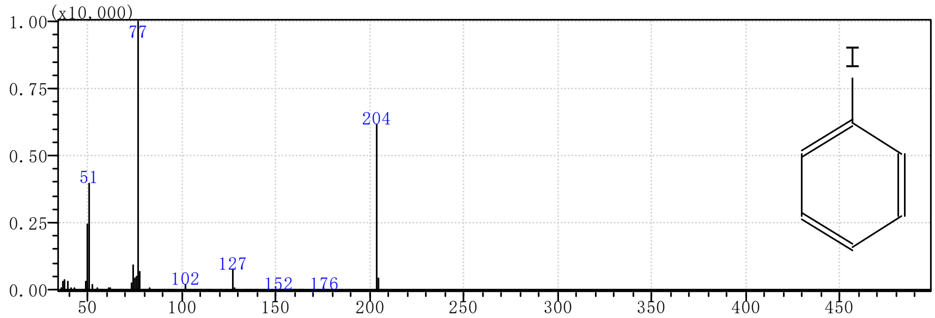

GC-MS: calcd. for C_6H_5I 204, found 204 (C_6H_5I). Table 4.

The peak 2 for t_R = 8.229 min with 4.333 % yield is:

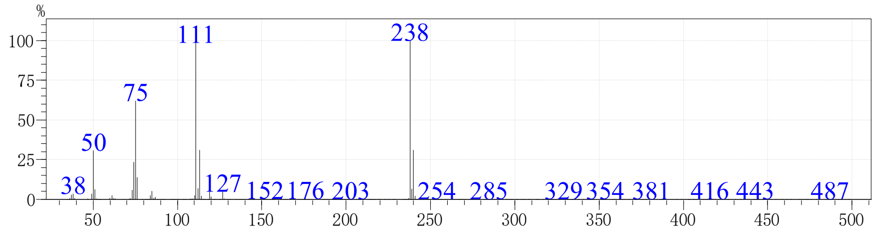

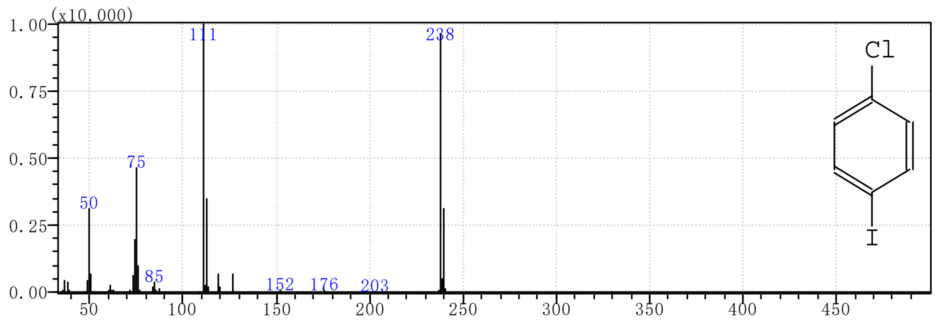

GC-MS: calcd. for C_6H_4ICl 238, found 238 (C_6H_4ICl). Table 4.

The peak 3 for t_R = 8.387 min with 3.600 % yield is:

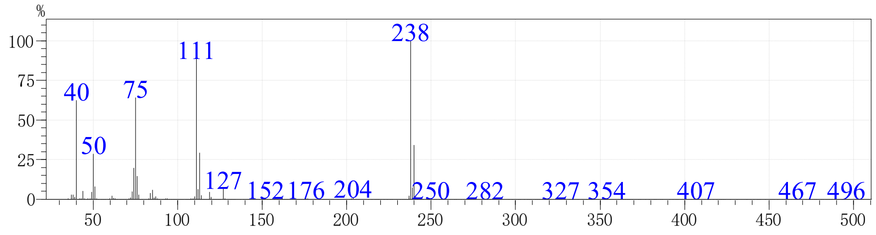

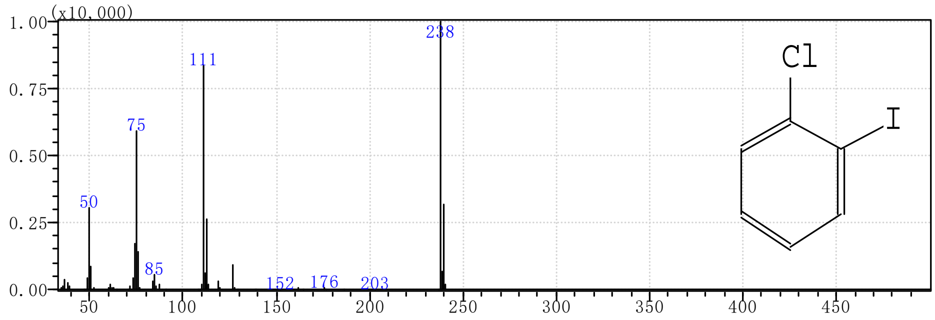

GC-MS: calcd. for C_6H_4ICl 238, found 238 (C_6H_4ICl). Table 4.

The peak 1 for t_R = 6.456 min with 92.067 % yield is unreacted iodobenzene. The peak 2 for t_R = 8.229 min with 4.333 % yield is 4-chloroiodobenzene. The peak 3 for t_R = 8.387 min with 3.600 % yield is 2-chloroiodobenzene.

(2) Entry 2, Table 4

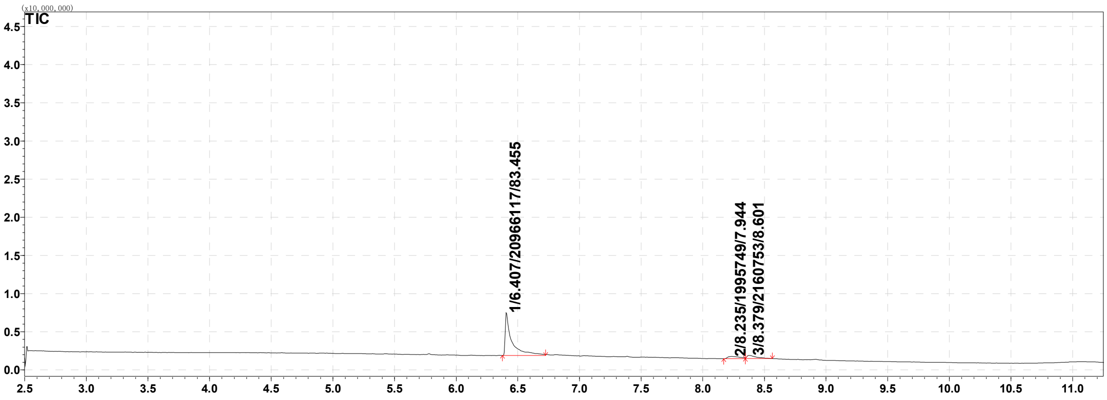

Figure S75. GC part of GC-MS for Entry 2, Table 4.

The peak 1 for t_R = 6.407 min with 83.455 % yield is unreacted iodobenzene. The peak 2 for t_R = 8.235 min with 7.944 % yield is 4-chloroiodobenzene. The peak 3 for t_R = 8.379 min with 8.601 % yield is 2-chloroiodobenzene.

(3) Entry 3, Table 4

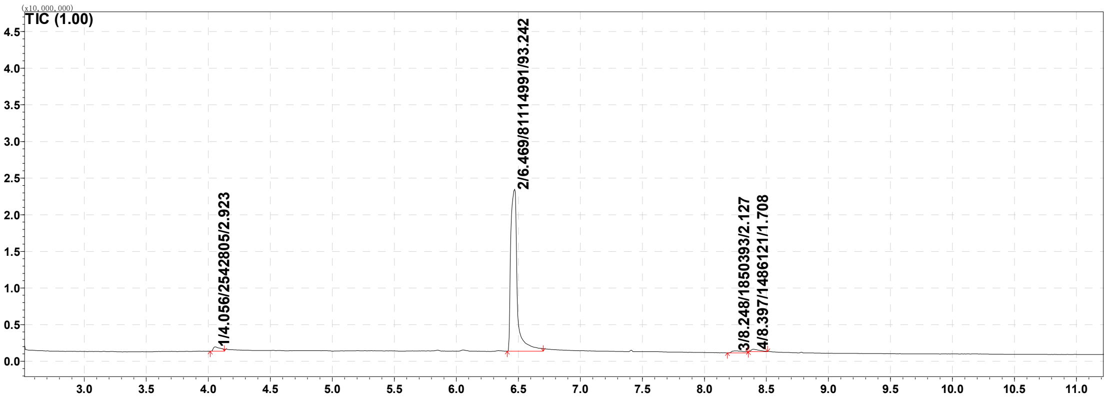

Figure S76. GC part of GC-MS for Entry 3, Table 4.

The peak 1 for t_R = 4.056 min with 2.923 % yield is:

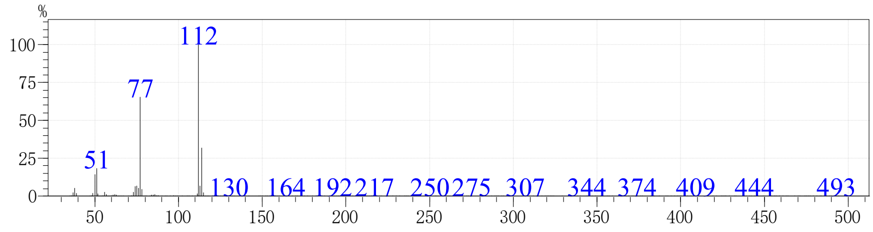

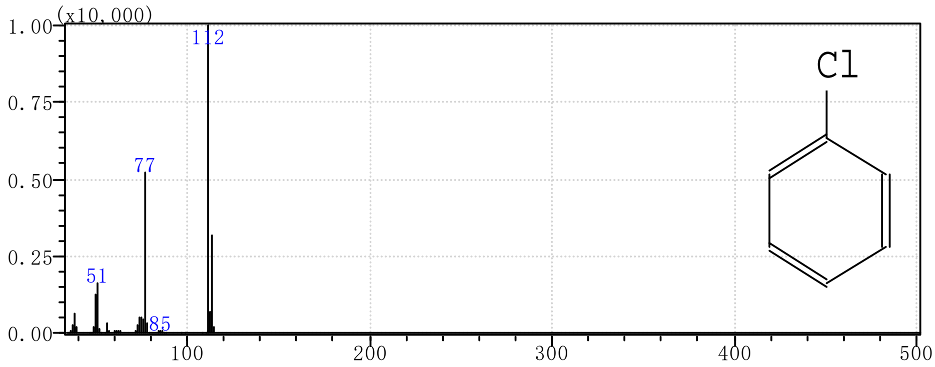

GC-MS: calcd. for C_6H_5Cl 112, found 112 (C_6H_5Cl). Table 4.

The peak 1 for t_R = 4.056 min with 2.923 % yield is chlorobenzene. The peak 2 for t_R = 6.469 min with 93.242 % yield is unreacted iodobenzene. The peak 3 for t_R = 8.248 min with 2.127 % yield is 4-chloroiodobenzene. The peak 4 for t_R = 8.397 min with 1.708 % yield is 2-chloroiodobenzene.

(4) Entry 4, Table 4

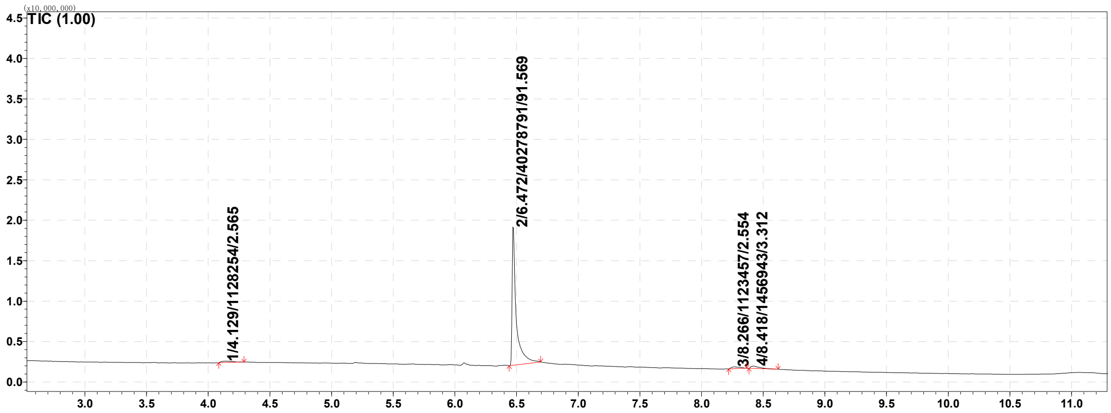

Figure S77. GC part of GC-MS for Entry 4, Table 4.

The peak 1 for t_R = 4.129 min with 2.565 % yield is chlorobenzene. The peak 2 for t_R = 6.472 min with 91.569 % yield is unreacted iodobenzene. The peak 3 for t_R = 8.266 min with 2.554 % yield is 4-chloroiodobenzene. The peak 4 for t_R = 8.418 min with 3.312 % yield is 2-chloroiodobenzene.

(5) Entry 5, Table 4

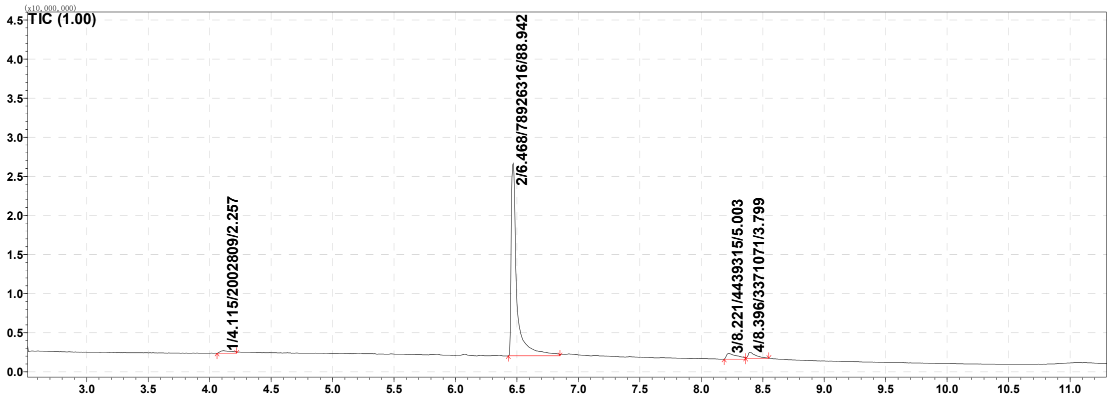

Figure S78. GC part of GC-MS for Entry 5, Table 4.

The peak 1 for t_R = 4.115 min with 2.257 % yield is chlorobenzene. The peak 2 for t_R = 6.468min with 88.942% yield is unreacted iodobenzene. The peak 3 for t_R = 8.221 min with 5.003 % yield is 4-chloroiodobenzene. The peak 4 for t_R = 8.396 min with 3.799 % yield is 2-chloroiodobenzene.

(6) Entry 6, Table 4

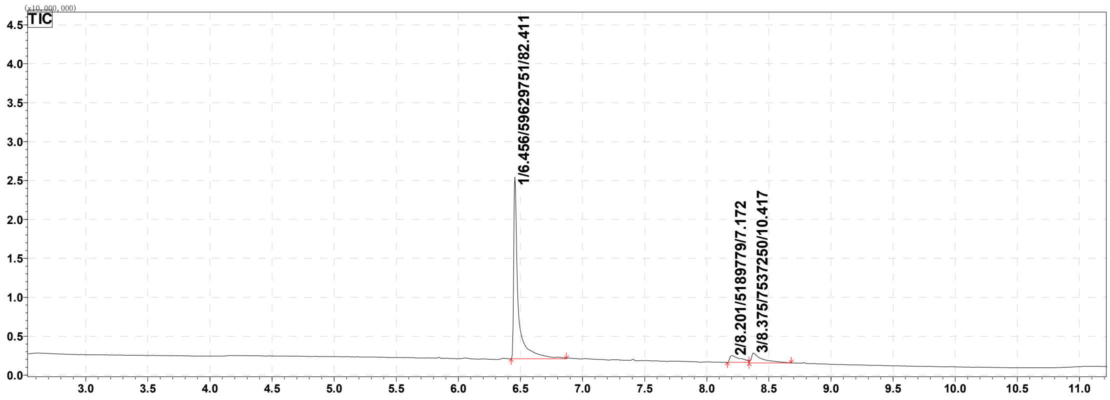

Figure S79. GC part of GC-MS for Entry 6, Table 4.

The peak 1 for t_R = 6.456 min with 82.411 % yield is unreacted iodobenzene. The peak 2 for t_R = 8.201 min with 7.172 % yield is 4-chloroiodobenzene. The peak 3 for t_R = 8.375 min with 10.417 % yield is 2-chloroiodobenzene.

(7) Entry 7, Table 4

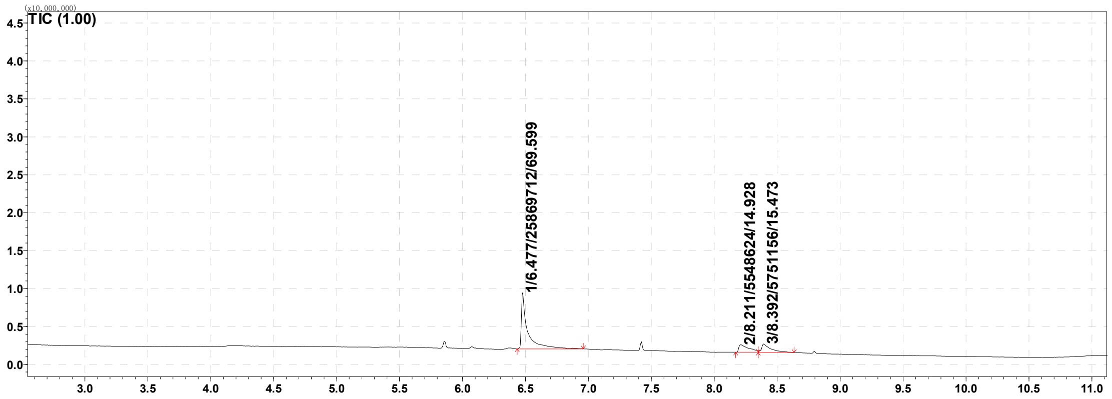

Figure S80. GC part of GC-MS for Entry 7, Table 4.

The peak 1 for t_R = 6.477 min with 69.599 % yield is unreacted iodobenzene. The peak 2 for t_R = 8.211 min with 14.928 % yield is 4-chloroiodobenzene. The peak 3 for t_R = 8.392 min with 15.473 % yield is 2-chloroiodobenzene.

(8) Entry 8, Table 4

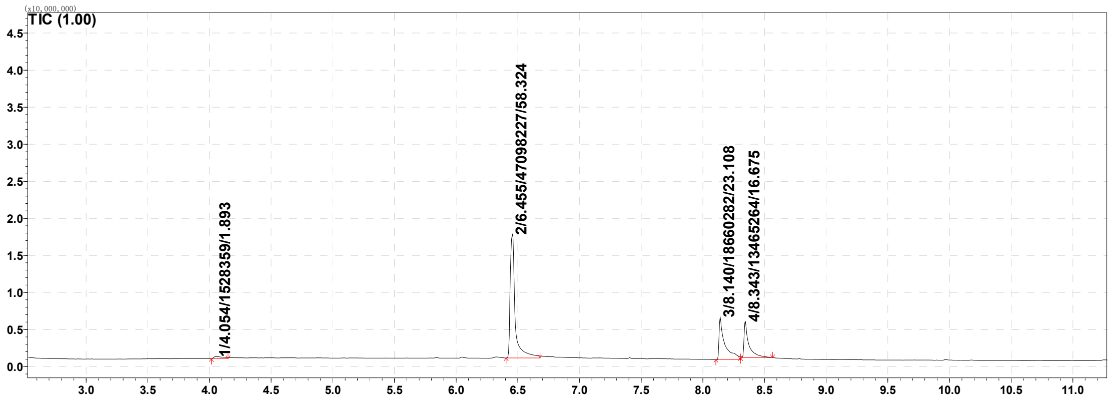

Figure S81. GC part of GC-MS for Entry 8, Table 4.

The peak 1 for t_R = 4.054 min with 1.893 % yield is chlorobenzene. The peak 2 for t_R = 6.455 min with 58.324 % yield is unreacted iodobenzene. The peak 3 for t_R = 8.140 min with 23.108 % yield is 4-chloroiodobenzene. The peak 4 for t_R = 8.343 min with 16.675 % yield is 2-chloroiodobenzene.

(9) Entry 9, Table 4

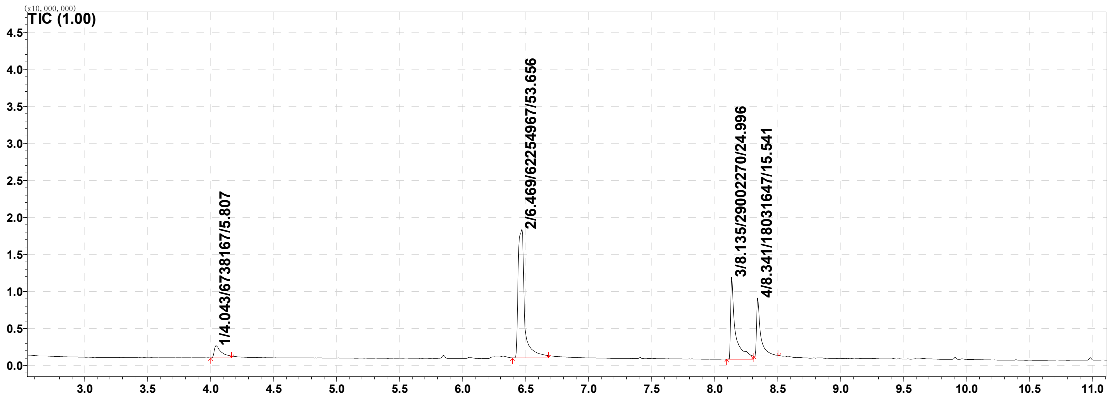

Figure S82. GC part of GC-MS for Entry 9, Table 4.

The peak 1 for t_R = 4.043 min with 5.807 % yield is chlorobenzene. The peak 2 for t_R = 6.469 min with 53.656 % yield is unreacted iodobenzene. The peak 3 for t_R = 8.135 min with 24.996 % yield is 4-chloroiodobenzene. The peak 4 for t_R = 8.341 min with 15.541 % yield is 2-chloroiodobenzene.
